# Presymptomatic, asymptomatic and post-symptomatic transmission of SARS-CoV-2: joint British Infection Association (BIA), Healthcare Infection Society (HIS), Infection Prevention Society (IPS) and Royal College of Pathologists (RCPath) guidance

**DOI:** 10.1186/s12879-022-07440-0

**Published:** 2022-05-12

**Authors:** Moira A. Mugglestone, Natasha V. Ratnaraja, Aggie Bak, Jasmin Islam, Jennie A. Wilson, Jennifer Bostock, Samuel E. Moses, James R. Price, Michael Weinbren, Heather P. Loveday, Lucy Rivett, Simon M. Stoneham, A. Peter R. Wilson

**Affiliations:** 1 Healthcare Infection Society, London, UK; 2 British Infection Association, Preston, UK; 3 University Hospitals Coventry & Warwickshire NHS Trust, Warwickshire, UK; 4 Warwick Medical School, Warwick, UK; 5 King’s College Hospital NHS Foundation Trust, London, UK; 6 Infection Prevention Society, Seafield, UK; 7 Richard Wells Research Centre, University of West London, London, UK; 8 Lay Member, London, UK; 9 East Kent Hospitals University NHS Foundation Trust, Kent, UK; 10 Royal College of Pathologists, London, UK; 11 Imperial College Healthcare NHS Trust, London, UK; 12 Imperial College London, London, UK; 13 Sherwood Forest Hospitals NHS Foundation Trust, Nottinghamshire, UK; 14 Cambridge University Hospitals NHS Foundation Trust, Cambridge, UK; 15 University College London Hospitals NHS Foundation Trust, London, UK

**Keywords:** SARS-CoV-2, COVID-19, Infection, Transmission, Presymptomatic, Asymptomatic, Post-symptomatic, Guideline

## Executive summary

This is the second of two guidance articles produced by the British Infection Association (BIA), the Healthcare Infection Society (HIS), the Infection Prevention Society (IPS) and the Royal College of Pathologists (RCPath). Both articles refer to the pandemic of coronavirus disease 2019 (COVID-19) caused by severe acute respiratory syndrome coronavirus 2 (SARS-CoV-2). Using evidence that emerged during the first wave of the pandemic, the articles summarise aspects of the transmission dynamics of SARS-CoV-2 and provide guidance on how to reduce the risk of transmission. This article focuses on the risks of presymptomatic, asymptomatic and post-symptomatic SARS-CoV-2 transmission, allowing healthcare workers and the public to understand how transmission occurs and to take action to protect themselves and others. The guidance recognises further waves of the pandemic, the possibility of reinfection, the emergence of new variants of the virus and ongoing immunisation programmes.

Having considered the evidence, the COVID-19 Rapid Guidance Working Party concluded that:

- presymptomatic transmission (meaning that an index case has no symptoms during the exposure period of their close contacts, but later develops symptoms) is **confirmed**
- asymptomatic transmission (meaning that an index case never develops symptoms or signs of infection) is **probable**.

The Working Party was unable to assess the likelihood of post-symptomatic transmission (meaning that an index case has no symptoms during the exposure period of their close contacts, but previously had symptoms) because of an absence of evidence.

The Working Party formulated recommendations for practice taking account of the evidence reviewed. The recommendations were developed for acute healthcare settings (with particular reference to clinical staff and infection prevention and control teams), but they might be useful in other health and care settings such as dental practices and care homes. The Working Party also identified areas for future research.

### Recommendations

Be aware that:

- people without noticeable symptoms may transmit the SARS-CoV-2 virus to other people
- transmission of SARS-CoV-2 from people without symptoms may occur in all settings in which people are in close proximity
- however, it is likely that the risk of transmission of SARS-CoV-2 is greater from people who have symptoms compared with those who do not.

Even in the absence of symptoms, adhere to legislation and guidance regarding measures to reduce the risk of transmission of SARS-CoV-2 (such as social distancing, hand hygiene, use of personal protective equipment and ventilation of enclosed spaces).

Be aware that the future transmissibility of SARS-CoV-2 from people carrying the virus without symptoms might depend on the:

- nature of further waves or outbreaks of COVID-19
- emergence and circulation of SARS-CoV-2 variants of concern
- potential for people who have had COVID-19 previously to be reinfected
- effectiveness of available vaccines, including the longevity of immunity they confer.

Be aware that it is not yet known to what extent or for how long people recovering from acute infection can transmit the SARS-CoV-2 virus to other people.

## Lay summary

Covid-19 is a worldwide problem, and we are learning not just how to treat and vaccinate (immunise) people, but also how and when the infection is spread from person to person. Unlike some infections, you cannot necessarily see who is likely to infect another person; this is because sometimes the infection is transmitted before (pre) someone develops symptoms. It is also the case that some people have the infection and can transmit it but never develop symptoms themselves; this we call asymptomatic transmission.

This guidance document is one of a pair which have reviewed the scientific evidence on how Covid-19 is spread. This part of the guide provides recommendations on how to help stop the spread of infection before someone becomes obviously ill (presymptomatic) and for those who never become ill themselves (asymptomatic). We did not find evidence for post symptomatic transmission (someone transmitting Covid-19 after they have recovered).

The recommendations based on the evidence we have reviewed give confidence that the things we are all doing such as social distancing, hand washing, wearing face coverings and keeping rooms well ventilated by opening windows are the things that we should be doing to prevent people getting infected with Covid-19. We hope that this guide will help everyone try and prevent spreading Covid-19.

## Introduction

Coronavirus disease 2019 (COVID-19) was first detected in Wuhan, Hubei province, China; it spread around the world as a pandemic and by November 2021 had affected more than 260 million people [1]. COVID-19 is caused by a beta-coronavirus, severe acute respiratory syndrome coronavirus 2 (SARS-CoV-2); other beta-coronaviruses associated with respiratory syndromes are severe acute respiratory syndrome coronavirus (SARS-CoV) and Middle East respiratory syndrome coronavirus (MERS-CoV).

As an emerging and pandemic disease, COVID-19 attracted worldwide attention and interest in understanding the dynamics of SARS-CoV-2 transmission and treatment options for COVID-19 patients. This Working Party Report is the second of two guidance articles developed using evidence published during the first wave of the pandemic to summarise aspects of the transmission dynamics of SARS-CoV-2 and advise on measures to reduce the risk of transmission in health and care settings. The article examines the risks of presymptomatic, asymptomatic and post-symptomatic SARS-CoV-2 transmission. Understanding the risk of transmission according to the index case’s symptom status at the time of exposure of (and potential transmission to) their close contacts is important to allow healthcare workers and the public to take action to protect themselves and others. The guidance acknowledges the possibility of reinfection, the emergence of new variants of the virus (particularly variants of concern), and ongoing immunisation programmes.

Key technical terms used in this guidance article are explained in the accompanying glossary (see Additional file 1: Appendix A).

## Guideline Development Team

### Acknowledgements

The authors would like to acknowledge the support of their employing institutions, which allowed time required for producing this guidance. We thank the National Institute for Health Research Biomedical Research Centre at University College London Hospitals, which partly supported APRW’s involvement in this guidance. We would also like to thank the Healthcare Infection Society (HIS) Guidelines Committee for reviewing this document.

### Source of funding

The authors received no specific funding for this work. Financial support for time required to identify and synthesise the evidence and to write the manuscript was provided by the authors’ respective employing institutions.

### Disclosure of potential conflicts of interest

No authors reported any conflicts of interest (see Additional file 1: Appendix B).

### Relationship of authors with sponsor

The British Infection Association (BIA), HIS, the Infection Prevention Society (IPS) and the Royal College of Pathologists (RCPath) commissioned the authors to develop the Working Party Report. The authors are members of the participating organisations and together comprise the COVID-19 Rapid Guidance Working Party convened to develop the guidance. MAM and AB are employed by HIS as guideline developers. Further information is provided in Additional file 1: Appendix B.

### Responsibility for the guidance

The views expressed in this publication are those of the authors and have been endorsed by BIA, HIS, IPS and RCPath following rapid consultation.

## Working party report

### What is the working party report?

This report is the second in a pair of guidance documents covering key aspects in the prevention of SARS-CoV-2 transmission in health and care settings. The guidance also reviews the evidence for SARS-CoV-2 transmission dynamics in broader settings. The diagnosis and management of COVID-19 in general is outside the remit of this guidance.

The Working Party recommendations have been developed systematically through multidisciplinary discussions based on currently available evidence from published, preprint and grey literature sources. They should be used in the development of local protocols for relevant health and care settings such as hospitals, nursing/care homes, primary care and dental practices.

### Why do we need a Working Party Report for this topic?

The first wave of the COVID-19 pandemic occurred amid uncertainty as to how it could be prevented and controlled. Concern still exists about further waves and new outbreaks occurring. Evidence that emerged during the first wave provides an opportunity to develop evidence-based guidance for preventing and controlling future waves/outbreaks, acknowledging the possibility of reinfection, the context of newly emerging variants of SARS-CoV-2, and ongoing immunisation programmes.

### What is the purpose of the Working Party Report’s recommendations?

The main purpose of the recommendations is to inform clinicians, managers and policy makers about SARS-CoV-2 transmission dynamics and to provide evidence-based guidance to prevent and control the spread of SARS-CoV-2 in health and care settings. The report highlights current gaps in knowledge, which will help to direct future areas of research.

### What is the scope of the guidance?

The scope of the guidance is to provide advice for the optimal provision of effective and safe health and care services during the period in which COVID-19 remains a health threat. The guidance was developed for acute healthcare settings, but it might be useful in other health and care settings such as dental practices and care homes.

### What is the evidence for the guidance?

Topics for this guidance were derived from initial discussions of the Working Party and specific review questions were developed in accordance with the population–exposure–comparator–outcome (PECO) framework for investigating the likelihood of developing a certain condition after an exposure event. To prepare the recommendations, the Working Party collectively reviewed relevant evidence from published, preprint and grey literature sources. The processes and methods used were in accordance with the National Institute for Health and Care Excellence (NICE) manual for developing guidelines (hereafter the NICE guidelines manual) [2]. The processes and methods were moreover aligned with those described in the first Working Party Report [3]. See below for further details.

### Who developed the guidance?

The Working Party included infectious diseases, microbiology and virology clinicians, academic infection prevention and control experts, systematic reviewers, and a lay representative.

### Who is the guidance for?

Any healthcare practitioner, manager or policy maker may use this guidance and adapt it for their use. It is anticipated that most users will be clinical staff and infection prevention and control teams. Some aspects of this guidance might also be beneficial to patients, their families/carers, and the public.

### How is the guidance structured?

To provide advice rapidly, the guidance is being produced as two separate articles, each addressing a different review question. Each article will comprise an introduction, a summary of the evidence, and recommendations graded according to the available evidence.

### How frequently is the guidance reviewed and updated?

The guidance will be considered for update within 1 year of publication to determine whether new evidence exists that would require a change in the recommendations.

### Aim

The aim of the guidance is to evaluate evidence for presymptomatic, asymptomatic and post-symptomatic transmission of SARS-CoV-2 with the intention of preventing transmission in hospitals and other health and care settings.

## Methodology

### Evidence search and appraisal

As noted above, the processes and methods used to produce this Working Party Report were aligned with those described in the first Working Party Report [3]. Topics for the COVID-19 rapid guidance were derived from initial discussions of the Working Party. An e-newsletter was sent to HIS members inviting further suggestions for topics to be considered. To develop their recommendations, the Working Party collectively reviewed evidence gathered from published, preprint and grey literature sources. The processes and methods used were based on the NICE guidelines manual [2]. Some modifications were made to allow a rapid review process to be followed. For example, the number of bibliographic databases searched was limited to two, the Working Party was smaller than usual (with only one lay member), and quality assessment was conducted by one reviewer (with 10% of records being checked by a second reviewer).

### Data sources and search strategy

Two electronic databases (MEDLINE and Embase) were searched for articles published between 1 January and 29 May 2020. Search terms were constructed using medical subject headings (MeSH) and free-text terms (see Additional file 1: Appendix C). Additional hand searching was conducted in several online databases (WHO Chinese database, CNKI, China Biomedical Literature Service, Epistemonikos COVID-19 L·OVE platform, EPPI-Centre living systematic map of the evidence, CORD-19, COVID-END, and HIS COVID-19 resources) to identify preprints, articles in press and grey literature. Reference lists from included studies and reviews identified through the literature searches were scanned for additional studies. Searches were restricted to person-to-person transmission of SARS-CoV-2 and no language restrictions were applied. Due to the large number of papers being published daily during the first and second waves of the pandemic, a decision was made not to rerun the searches before publication as this would significantly delay the guidance being made available to readers. Further details of the searches are presented in Additional file 1: Appendix C.

### Study eligibility and selection criteria

The members of the Working Party determined study inclusion criteria. Any article presenting primary data on presymptomatic, asymptomatic or post-symptomatic transmission of SARS-CoV-2 was eligible for inclusion. Search results were screened for relevance, with one reviewer examining titles, abstracts and full texts of all records identified through the searches. A second reviewer checked at least 10% of records earmarked for exclusion at each stage of screening. Disagreements were first discussed between the two reviewers and, if consensus was not reached, a third reviewer was consulted. The results are presented in the study selection flowchart in Additional file 1: Appendix D. A list of studies excluded after full-text screening is presented in Additional file 1: Appendix E.

### Data extraction, analysis and quality assessment

The characteristics of included studies are summarised in Additional file 1: Appendix F. For each included study, data were extracted into an evidence table by one reviewer while a second reviewer checked the data extraction for 10% of studies. Evidence was stratified (organised) according to the type of study (cluster/outbreak investigations, comparative epidemiological studies, and mathematical modelling of epidemic spread). The resulting evidence tables are presented in Additional file 1: Appendix G.

Further stratification of the evidence, for example, according to whether a cluster/outbreak investigation explored the possibility of presymptomatic transmission (in which the index case had no symptoms during the exposure period of their close contacts, but later developed symptoms) or asymptomatic transmission (in which the index case never developed symptoms or signs of infection) was undertaken to aid presentation and interpretation of the evidence.

Many of the cluster/outbreak investigations permitted only a categorical (non-numerical or nominal) assessment of the credibility of transmission by presymptomatic or asymptomatic people (with the categories assigned in the evidence review being ‘yes’, ‘no’ or ‘uncertain’). Other cluster/outbreak investigations allowed calculation of an attack rate (the number of contacts of the index case who tested positive for SARS-CoV-2 divided by the total number of contacts) and an associated confidence interval (CI). Stratification of the evidence from cluster/outbreak studies according to the time at which contacts were exposed to SARS-CoV-2 relative to the index case acquiring the virus (categorised as < 7 days, 7 to 10 days, 11 to 14 days or not calculable, with day 0 representing the day on which the index case acquired the virus) was also undertaken.

Where cluster/outbreak studies reported the use of personal protective equipment (PPE) this was noted to aid interpretation of the evidence.

The possibility of identifying comparative epidemiological studies relevant to the review question had not been anticipated because the pandemic was associated with a novel disease and was still in its early stages when the evidence review was initiated. However, several such studies were identified and included as noted above. For these epidemiological studies (and the mathematical modelling studies included in the review—see below) that reported (or allowed calculation of) a measure of transmission risk according to the index case’s symptom status at the time of exposure of their close contacts, the convention of expressing risks based on exposure to people with fewer symptoms compared to risks based on exposure to people with more symptoms was applied where possible.

Mathematical modelling studies were included in the review only where they distinguished between transmission risks according to the index case’s symptom status during exposure of their close contacts.

Included epidemiological studies were appraised for quality using checklists recommended in the NICE guidelines manual [2]. Critical appraisal was conducted by one reviewer, and appraisal outcomes for at least 10% of studies were checked by a second reviewer. The results of study-level quality appraisal are included in the evidence tables in Appendix G. Mathematical modelling studies were not appraised for quality at individual study level.

### Rating of evidence and recommendations

Evidence was assessed for quality at outcome level using the approach known as Grading of Recommendations Assessment, Development and Evaluation (GRADE; see https://www.gradeworkinggroup.org/ for details). The resulting GRADE tables are presented in Additional file (1) (stratified by type of study and, in the case of cluster/outbreak investigations, exploration of presymptomatic or asymptomatic transmission and time at which contacts were exposed to SARS-CoV-2 relative to the index case acquiring the virus, as outlined above). Using GRADE, the overall quality of the evidence for a particular outcome was classified as very low, low, moderate, or high.

No overall assessment of the quality of evidence from mathematical modelling studies was conducted using GRADE because there is no validated approach for applying GRADE to such studies. However, some domains in the GRADE framework are applicable in the case of mathematical modelling studies, for example, inconsistency and indirectness. All the evidence from the mathematical modelling studies was downgraded for indirectness by at least one level because such studies provided indirect estimates of transmission risks compared to epidemiological studies. Further downgrading for indirectness was assessed on a case-by-case basis (see Additional file 1: Appendix H for details).

Evidence statements were constructed by combining the outcome-level classification of evidence quality determined using GRADE and the following terms reflecting the Working Party’s overall confidence in using the evidence to formulate recommendations:

- strong evidence—further research is unlikely to alter confidence in the estimated effect
- moderate evidence—further research might alter the estimated effect and its strength
- weak evidence—further research is very likely to alter the estimated effect and its strength
- inconsistent evidence—current studies report conflicting evidence and further research is very likely to alter the estimated effect.

The Working Party further classified the evidence as indicating whether presymptomatic, asymptomatic and post-symptomatic transmission was confirmed, probable, possible, unlikely, or confirmed as not occurring. This mirrored the approach taken in the first article in the pair of guidance documents, which examined routes of transmission of SARS-CoV-2 [3].

Finally, in accordance with the GRADE approach, the Working Party’s recommendations were phrased to reflect the strength of the evidence and their confidence in using it as the basis for developing recommendations.

Where there was little or no evidence to guide recommendations, the Working Party used informal consensus to formulate ‘good practice recommendations’ based on their collective experience and expertise.

Videoconferences were held regularly throughout the guideline development process to discuss and interpret the evidence and translate it into recommendations for practice (and, where gaps in the evidence were identified, recommendations for further research).

### Consultation process

Feedback on the draft guidance was received from the HIS Guidelines Committee and through rapid consultation with relevant stakeholders. The draft report was placed on the HIS website for 10 working days along with the HIS standard response form, including a conflict-of-interest disclosure form. The availability of the draft guidance was communicated via email and social media. Stakeholders were invited to comment on format, content, local applicability, patient acceptability and recommendations. The Working Party reviewed stakeholder comments, and collectively agreed revisions in response to the comments (see Additional file 1: Appendix I). Comments received from individuals who disclosed conflicts of interest, or who did not submit a conflict-of-interest disclosure form, were excluded.

## Results

### Overview of the evidence

Fifty-five articles were included in the evidence review (see Additional file 1: Table SF.1) [4–58]. Of these, 44 reported cluster/outbreak investigations (presented in chronological order in Additional file 1: Table SG.1) [4–7, 9, 10, 14, 15, 17, 18, 20–28, 30–34, 36–44, 46–51, 53–55, 57, 58], six reported comparative epidemiological studies that allowed calculation of relative risks of transmission based on the index case’s symptom status during exposure of their close contacts (for example, transmission associated with presymptomatic exposure versus transmission associated with symptomatic exposure) [11, 12, 19, 35, 52, 56], and five reported mathematical modelling of epidemic spread [8, 13, 16, 29, 45]. More than half of the included studies referred to investigations of SARS-CoV-2 transmission in mainland China, reflecting the emergence and initial investigation of COVID-19 there; the remainder reported evidence from Germany, Hong Kong, Italy, Japan, Malaysia, Singapore, South Korea, Switzerland, Taiwan, USA and Vietnam, reflecting the pandemic spread as time progressed (see Additional file 1: Table SF.1 for further details).

### Cluster/outbreak investigations

In several instances, the same cluster/outbreak was reported independently in more than one article (for example, three separate articles reported or commented on a single cluster/outbreak in Germany) [7, 26, 41] or the same data were analysed differently across multiple articles (for example, three articles reported different analyses of relative risks of transmission based on the index case’s symptom status during an outbreak in China) [11, 19, 52]. Similarly, there were several instances in which a single article reported multiple clusters/outbreaks (for example, one article summarised evidence from several clusters in Singapore that were likely to be associated with presymptomatic transmission) [46]. Accounting for such overlaps by presenting a combined summary of each distinct cluster/outbreak or other epidemiological analysis resulted in a total of 45 distinct clusters/outbreaks and four sets of comparative epidemiological analyses of transmission risks based on symptom status (see Additional file 1: Tables SG.1 and G.2 for further details).

The reported cluster/outbreak investigations focused on potential transmission of SARS-CoV-2 in both community and nosocomial settings (see Additional file 1: Tables SF.1 and G.1). The possibility of presymptomatic transmission was explored in more studies (36 clusters/outbreaks) [4, 5, 7, 9, 10, 15, 17, 18, 20, 21, 23–28, 30–33, 36, 39–41, 43, 44, 46–51, 53, 54, 57, 58] than was the possibility of asymptomatic transmission (seven clusters/outbreaks) [6, 14, 22, 34, 38, 42, 55]; two further clusters/outbreaks were reported in sufficient detail to determine that presymptomatic or asymptomatic (rather than symptomatic) exposure had occurred, but not to distinguish between the two (see Additional file 1: Table SG.1) [36, 37]. There were no reports of investigations exploring the possibility of post-symptomatic transmission.

Stratification of the evidence from cluster/outbreak investigations according to the time at which contacts were exposed to SARS-CoV-2 relative to the index case acquiring the virus (< 7 days, 7 to 10 days, 11 to 14 days or not calculable) is reflected in the evidence tables for the cluster/outbreak studies (see Additional file 1: Table SG.1) and the corresponding GRADE tables (see Additional file 1: Table SH.1, H.2 and H.3).

### Comparative epidemiological studies

Relative risks of transmission associated with presymptomatic exposure versus transmission associated with symptomatic exposure (two studies) [12, 35], and transmission associated with asymptomatic exposure compared to either presymptomatic or symptomatic exposure (four studies reported across six articles) [11, 12, 19, 35, 52, 56] are presented in the evidence tables for the comparative epidemiological studies (see Additional file 1: Table SG.2) and the corresponding GRADE table (Additional file 1: Table SH.4).

### Mathematical modelling studies

Three of the mathematical modelling studies included in the review used adaptations of the susceptible–exposed–infected–recovered (SEIR) compartmental modelling framework to model transmission dynamics in hypothetical populations [16, 29, 45]. Other approaches reflected in the included studies involved application of a renewal equation framework (one study) [13] and modelling of viral emissions resulting from respiratory and physical activity in indoor commercial environments (such as a supermarket or restaurant) allowing for different ventilation characteristics (one study) [8]. Further details are presented in the evidence tables for the mathematical modelling studies (see Additional file 1: Table SG.3) and the corresponding GRADE tables (see Additional file 1: Table SH.5 and H.6).

### Quality of the evidence

For each type of study for which it was possible to produce an overall GRADE rating of the quality of the evidence the rating applied was very low (see Additional file 1: Appendix H). This was partly due to observational studies being assigned an initial rating of low quality, which would be downgraded to very low if even one serious limitation were identified with the evidence.

Frequently occurring reasons for downgrading the quality of evidence from cluster/outbreak investigations were risk of bias associated with a lack of clarity regarding complete inclusion (for example, because it was not clear whether all contacts of an index case had been accounted for) and imprecision associated with no CIs or other measures of precision being reported (or calculable). Among those cluster/outbreak investigations that evaluated the risk of asymptomatic transmission, several had evidence downgraded for indirectness because the definition of an asymptomatic infection included having mild symptoms (such as a pre-existing cough that might or might not have been associated with or exacerbated by SARS-CoV-2 infection), or signs of infection on a computerised tomography (CT) scan of the chest. See Additional file 1: Table SH.1, H.2 and H.3 for further details.

Another aspect of the evidence from the cluster/outbreak investigations was the use of PPE as recorded in the evidence tables for these studies (see Additional file 1: Table SG.1) and the corresponding GRADE tables (see Additional file 1: Table SH.1, H.2 and H.3). One investigation exploring the possibility of presymptomatic transmission reported that the index case (a transplant surgeon) and their clinical colleagues used PPE during the index case’s presymptomatic phase (the index case used hand hygiene and wore a surgical mask and gloves for preoperative visits and standard surgical procedures, while clinical colleagues wore surgical masks at distances of less than 1 m and gloves during all contact) [40]. One investigation exploring the possibility of asymptomatic transmission reported that during hospital quarantine of the index case, the index case and other patients and visitors wore masks except when eating or drinking, while hospital staff wore N95 respirators, isolation gowns and goggles [14]. Another investigation exploring the possibility of asymptomatic transmission reported that the index case wore a mask while travelling to a health clinic, during the clinic visit, and while in the same room as their housemates after returning home [42].

Among the comparative epidemiological studies that reported (or allowed calculation of) relative measures of transmissibility according to the index case’s symptom status during exposure of their close contacts, a frequently occurring reason for downgrading the quality of the evidence was risk of bias associated with potential confounding factors (for example, age or a pre-existing condition that might affect susceptibility to infection) not being accounted for in the design or analysis of the study. Another common reason for downgrading the quality of evidence from such studies was that CIs for estimated effects crossed default thresholds for defining imprecision according to the GRADE approach. See Additional file 1: Table SH.4 for further details.

The quality of the evidence from the mathematical modelling studies included in the review was downgraded for indirectness in several cases because relative measures of transmissibility according to the index case’s symptom status during exposure of their close contacts were not wholly aligned with the symptom statuses of interest to the Working Party (that is, presymptomatic and asymptomatic infections). In one such study, asymptomatic infections and mildly symptomatic infections were grouped together [16]. Another study characterised infections as being ‘undocumented’ (defined as lacking symptoms severe enough to be confirmed/observed) or ‘documented’ (defined as having symptoms severe enough to be confirmed/observed) [29]. A third study incorporated asymptomatic viral load estimates that might be more representative of presymptomatic or symptomatic viral loads; this study distinguished between asymptomatic and symptomatic infections only in terms of respiratory and physical activity levels modelled [8]. See Additional file 1: Table SH.5 and H.6 for further details.

## Evidence statements

### Absolute transmissibility of presymptomatic and asymptomatic infections

There was strong evidence from 36 cluster/outbreak investigations (some of which were reported across multiple articles, as noted above) [4, 5, 7, 9, 10, 15, 17, 18, 20, 21, 23–28, 30–33, 36, 39–41, 43, 44, 46–51, 53, 54, 57, 58] regarding the possibility of SARS-CoV-2 being transmitted by presymptomatic people. Conclusive evidence of presymptomatic transmission was provided for seven clusters/outbreaks [21, 23, 28, 31, 33, 36, 46, 51, 53, 54]. For another 27 clusters/outbreaks it was uncertain whether presymptomatic transmission had occurred [5, 7, 9, 10, 15, 17, 18, 20, 24–28, 30, 32, 39, 41, 43, 44, 46–50, 57, 58]. In the two remaining clusters/outbreaks presymptomatic transmission did not occur: one of these related to potential community transmission associated with tourism in which the index case was assumed to have acquired SARS-CoV-2 in China before travelling to South Korea on holiday, but the timing of acquisition of the virus by the index case was uncertain [4]; the other related to potential nosocomial transmission associated with a transplant surgery department in which the index case (a transplant surgeon) used hand hygiene and wore a surgical mask and gloves for preoperative visits and standard surgical procedures, while clinical colleagues wore surgical masks at distances of less than 1 m and gloves during all contact [40]. Among the seven clusters/outbreaks for which presymptomatic transmission was demonstrated, in one instance the index case had acquired the virus less than 7 days previously [21] and in another less than 13 days previously [23]; the contacts’ exposure period relative to the index case acquiring the virus was not calculable for the remaining clusters/outbreaks [31, 33, 36, 46, 51, 53, 54]. Attack rates were calculable for only three of the seven clusters/outbreaks for which presymptomatic transmission was demonstrated (attack rate 40% based on 22 close contacts of the index case [23], 85% based on 13 close contacts [21] and 100% based on one close contact) [31]. The settings in which presymptomatic transmission was demonstrated to occur related to community transmission (via households, gatherings of family and friends, a work meeting, being in a restaurant, attending church, or sharing transport).

There was moderate evidence from seven cluster/outbreak investigations [6, 14, 22, 34, 38, 42, 55] regarding the possibility of SARS-CoV-2 being transmitted by asymptomatic people. Conclusive evidence of asymptomatic transmission was provided for one cluster/outbreak [22]. For another four clusters/outbreaks it was uncertain whether asymptomatic transmission had occurred [6, 34, 38, 55]. In the two remaining clusters/outbreaks asymptomatic transmission did not occur: one of these related to potential community and nosocomial transmission associated with exposure of the index case’s household, rideshare partners and healthcare workers at a clinic attended by the index case – the index case wore a mask while travelling to the clinic, during the clinic visit and while in the same room as members of their household after returning home; the other related to potential nosocomial transmission associated with hospital quarantine of the index case after presenting at the emergency department – the index case, other patients and visitors all wore masks except when eating or drinking, while hospital staff wore N95 respirators, isolation gowns and goggles [14]. In both instances, the index case had respiratory symptoms attributable to causes other than COVID-19. In the cluster/outbreak for which asymptomatic transmission was demonstrated, the index case had acquired the virus less than 7 days previously [22]. The attack rate for this cluster/outbreak was 100% (based on 3 close contacts of the index case) and the setting was related to community transmission (via the index case’s household). Although the index case was asymptomatic, they had signs typical of viral infection on a CT scan of the chest.

There was weak evidence from two further cluster/outbreak investigations [36, 37] regarding the possibility of SARS-CoV-2 being transmitted by presymptomatic or asymptomatic people. For these clusters/outbreaks it was not possible to determine whether the index case ever developed symptoms and it was uncertain whether transmission occurred.

### Relative transmissibility of presymptomatic and asymptomatic infections

There was moderate evidence from four epidemiological studies reported across six articles [11, 12, 19, 35, 52, 56] regarding relative transmissibility of presymptomatic, asymptomatic and symptomatic people. No differences in transmission according to symptom status of the index case during the exposure period of their close contacts were detected, although there was a trend towards fewer symptoms in the index case being associated with a lower risk of transmission: presymptomatic versus symptomatic exposure, odds ratio (OR) 0.22 (95% CI 0.01 to 3.86) [35] and OR 0.79 (95% CI 0.18 to 3.40) [12]; asymptomatic versus symptomatic exposure, OR 0.57 (95% CI 0.03 to 10.80) [35], OR 0.63 (95% CI 0.04 to 10.44) [12], OR 0.64 (95% CI 0.28 to 1.47) [11, 19, 52] and OR 0.83 (95% CI 0.36 to 1.92) [11, 19, 52]; and asymptomatic versus presymptomatic exposure, OR 0.17 (95% CI 0.02 to 1.34) [56]. Conclusive evidence of presymptomatic transmission was provided by two of the epidemiological studies [12, 56]; conclusive evidence of asymptomatic transmission was provided by two of the studies reported across four articles [11, 19, 52, 56], although the definition of an asymptomatic infection was not always reported. Mass testing might have played a role in preventing asymptomatic transmission in two of the studies [12, 35] because asymptomatic people might have self-isolated from household members when informed about their possible infection.

There was inconsistent evidence from four mathematical modelling studies [13, 16, 29, 45] regarding relative transmissibility according to symptom status of the index case during the exposure period of their close contacts. Fewer symptoms in the index case during exposure of close contacts was associated with a lower risk of transmission in one study: undocumented infections (assumed to be associated with fewer symptoms) versus documented infections (assumed to be associated with more symptoms), risk ratio (RR) 0.42 (95% credible interval (CrI) 0.34 to 0.61) and RR 0.47 (95% CrI 0.36 to 0.64) with containment measures such as travel restrictions and contact precautions, and RR 0.55 (95% CrI 0.49 to 0.60) without containment measures [29]. Another study reported a lower risk of transmission by people who were infectious but asymptomatic compared to those who were infectious with symptoms, RR 0.81 (95% CrI not reported) [45]. Another study reported a higher risk of transmission by infected people with severe symptoms compared to people who were asymptomatic or had mild symptoms, RR 1.03 (95% CrI 0.79 to 1.38) [16]. The same study reported a lower risk of transmission by people who were asymptomatic or had mild symptoms compared to those who were presymptomatic, RR 0.033 (95% CrI 0.027 to 0.036) [16]. The remaining study reported percentages of the total reproduction number accounted for presymptomatic, asymptomatic and symptomatic transmission (presymptomatic transmission, 47% (95% CrI 11% to 58%), asymptomatic transmission, 6% (95% CrI 0% to 57%), and symptomatic transmission, 28% (95% CrI 9% to 49%)) [13].

There was weak evidence from one mathematical modelling study [8] regarding the relative transmissibility of asymptomatic infections according to ventilation characteristics in indoor commercial environments. Asymptomatic transmission reproduction numbers with mechanical ventilation were lower than those with natural ventilation (supermarket, 0.12 with mechanical ventilation versus 0.17 with natural ventilation; post office, 0.17 with mechanical ventilation versus 0.41 with natural ventilation; pharmacy, 0.22 with mechanical ventilation versus 0.49 with natural ventilation; bank, 0.34 with mechanical ventilation versus 0.81 with natural ventilation; estimates refer to modelling of lockdown in which restaurants were required to close and additional voluntary measures included fewer staff on duty, customers queueing outside, and ventilation increased by keeping external doors open; estimates for restaurant without lockdown, 5.35 with mechanical ventilation versus 47.3 with natural ventilation; no CIs or other measures of precision reported).

### Transmissibility of post-symptomatic infections

No evidence was identified regarding the possibility of SARS-CoV-2 being transmitted by post-symptomatic people.

## Rationale for recommendations

### Outcomes that matter most

The Working Party’s interest focused on whether transmission occurs as a result of presymptomatic, asymptomatic or post-symptomatic SARS-CoV-2 infection. For the most part, this was evaluated through consideration of absolute risks of transmission. At the start of the evidence review process, it was not anticipated that relative risks of transmission based on the symptom status of an index case would have been examined (because the pandemic was in its early stages and research was just starting to be published). However, it became evident when sifting the results of the systematic literature searches that some studies had investigated relative risks of transmission and this evidence was eligible for inclusion according to the review protocol.

### Quality of the evidence

The evidence from the cluster/outbreak investigations and epidemiological studies providing estimates of relative risks of transmission based on an index case’s symptom status during exposure of their close contacts was assessed for quality using the GRADE framework. All of the evidence from these studies was classified as being of very low quality. Recurring reasons for downgrading the evidence included: risk of bias (for example, due to lack of clarity regarding complete inclusion of an index case’s close contacts in the case of cluster/outbreak investigations, and potential confounding factors (such as pre-existing conditions and strength of the immune system) not being accounted for in the case of epidemiological studies providing relative risks of transmission based on the index case’s symptom status during exposure of close contacts); imprecision due to CIs for effect estimates crossing predefined thresholds or being unavailable; and indirectness (for example, in studies investigating potential asymptomatic transmission the definition of an asymptomatic infection sometimes included having mild symptoms or signs of infection). The overall assessment of the evidence as being of very low quality did not, however, prevent the Working Party reaching conclusions about characteristics of SARS-CoV-2 transmission and making recommendations for practice (see below).

The evidence from the mathematical modelling studies included in the review could not be fully assessed using the GRADE framework, but some GRADE domains were applicable, for example, inconsistency and indirectness. A recurring reason for downgrading the evidence from these studies was indirectness due to relative measures of transmissibility according to an index case’s symptom status during exposure of close contacts not being fully aligned with symptom statuses of interest to the Working Party (in particular, presymptomatic and asymptomatic infections).

### Benefits and harms

Having considered the evidence, the Working Party concluded that:

- presymptomatic transmission (meaning that an index case has no symptoms during the exposure period of their close contacts, but later develops symptoms) is **confirmed**
- asymptomatic transmission (meaning that an index case never develops symptoms or signs of infection) is **probable**.

There was uncertainty regarding the evidence related to asymptomatic transmission, with the Working Party noting that a lack of awareness of symptoms or suppressed symptoms (for example, due to taking medication) could not be distinguished from a complete absence of symptoms in the reported investigations. The Working Party recognised the potential for subclinical or pauci-symptomatic infection while emphasising that truly asymptomatic infection or carriage of SARS-CoV-2 occurs and transmission is to be expected [59].

The Working Party recognised that the list of symptoms suggesting COVID-19 had expanded during the pandemic, reflecting growing knowledge of the condition. The evidence review and synthesis involved extracting any information about symptoms reported by the study investigators, although it was acknowledged that people’s perceptions of symptoms differ and this could influence the types of symptoms reported. The Working Party emphasised the importance of clarity in defining and reporting symptoms in future research related to COVID-19.

The settings in which presymptomatic or asymptomatic transmission was demonstrated mirrored those reported in the first of the pair of guidance articles in which routes of transmission, regardless of the symptom status of the index case, were explored [3]. In particular, presymptomatic transmission was demonstrated to occur in community settings that included households, gatherings of family and friends, a work meeting, being in a restaurant, attending church, or sharing transport. The Working Party agreed that transmission in the absence of noticeable symptoms could similarly occur in health and care settings that involve people being in close proximity.

The Working Party agreed that from the perspective of preventing transmission by people without symptoms, it is immaterial whether they later develop symptoms. The recommendations were therefore phrased in terms of people without symptoms rather than using the terms presymptomatic and asymptomatic. The Working Party anticipated that this phrasing would also make the recommendations more meaningful to the public.

The benefits of preventing transmission of SARS-CoV-2 by people without symptoms include the prevention of ill health due to COVID-19 among their close contacts and the prevention of onward transmission to ever greater numbers of people. Possible harms associated with actions intended to prevent transmission of SARS-CoV-2 (such as social distancing, hand hygiene and the use of PPE) arise through restriction of personal freedoms and a need to modify behaviours with potential adverse consequences in terms of, for example, mental health and wellbeing. These benefits and harms apply to healthcare workers, patients and their families/carers, and the public. On balance, the Working Party recognised that since anyone might carry the virus without knowing it, or be infected without having noticeable symptoms, the recommendations should reinforce the importance of adhering to existing legislation and guidance intended to reduce the risk of transmission of SARS-CoV-2 in the general population.

The Working Party noted that the evidence regarding relative risks of transmission according to symptom status suggested that presymptomatic infections are less transmissible than are symptomatic infections, and that asymptomatic infections are less transmissible than are presymptomatic infections. The Working Party was aware that the viral load associated with asymptomatic and pauci-symptomatic infections is typically lower than that associated with symptomatic infection [59], lending plausibility to a lower rate of transmission. Based on the available evidence, the Working Party therefore agreed that the recommendations should highlight the likelihood of greater transmissibility from people with symptoms than from those without symptoms. Due to some uncertainty remaining, the Working Party also prioritised relative risks of transmission, including the correlation between transmission and quantification of viral shedding, as an area for future research.

Although the evidence from the mathematical modelling studies was regarded as indirect, the Working Party noted the reported differences in asymptomatic transmission rates in indoor environments under different ventilation scenarios. This prompted the Working Party to emphasise the importance of ventilation in enclosed spaces in the recommendations.

The Working Party was acutely aware that the development of the guidance was occurring during an evolving pandemic. When formulating the recommendations, the Working Party recognised the possibility of reinfection in people who previously had COVID-19 [60], the emergence of variants of concern, and ongoing immunisation programmes. As such, the Working Party highlighted in the recommendations that the characteristics and implications of transmission of SARS-CoV-2 by people without symptoms might change in the future.

The likelihood of post-symptomatic transmission (meaning that an index case has no symptoms during the exposure period of their close contacts, but previously had symptoms) could not be assessed because of an absence of evidence. The Working Party agreed that post-symptomatic transmission should be prioritised as an area for further research.

### Cost effectiveness and resource use

The Working Party did not undertake a detailed economic analysis because the recommendations focused on raising awareness of the possibility of presymptomatic and asymptomatic transmission of SARS-CoV-2 and reinforcing existing legislation and guidance aimed at preventing transmission. However, the Working Party considered costs and resource use from the perspective of health and care systems and identified that costs associated with transmission that is not prevented include the costs of managing COVID-19 in infected patients and the costs of needing additional resources such as PPE. Considerations related to the value of time as a resource included the time taken to don and doff PPE and time away from work for healthcare workers who are unwell or required to self-isolate. Taken together, these considerations emphasise increased pressure on healthcare systems when COVID-19 is prevalent. The Working Party recognised potential inconvenience and possible adverse consequences (in terms of mental health and wellbeing of healthcare workers, patients and their families/carers) of implementing measures such as social distancing and using PPE. The Working Party also recognised that the cost effectiveness of preventing transmission would be greater in aspects of healthcare focusing on people more vulnerable to COVID-19.

### Other considerations

As outlined above, the Working Party highlighted several areas for future research. These included consideration of:

- when a person who has acquired SARS-CoV-2 becomes infectious and
- how long infectivity lasts in the absence of symptoms.

While the evidence available to the Working Party demonstrated presymptomatic transmission within 7 days of an index case acquiring the virus, later transmission could not be ruled out. Moreover, the available evidence did not permit a detailed analysis of infectivity during the first 7 days since acquiring the virus, which was of interest to the Working Party and could form part of future research. The Working Party also highlighted potential seasonality in transmission rates, and indoor versus outdoor transmission, as areas to explore in future research.

The Working Party discussed the relevance and possible consequences of lung damage revealed by CT scans in people who did not report symptoms. The Working Party questioned whether such features might have longer-term consequences for a person who although infected has no noticeable symptoms and recommended this as an area for future research.

The Working Party made several observations regarding the quality of the evidence identified in the review. While the importance of rapid evaluation during a pandemic caused by a novel disease such as COVID-19 was appreciated, the value in ensuring robust and efficient research activity was also recognised. The Working Party agreed that this value could be promoted by avoiding duplication and repetition in data collection, analysis, and reporting, and acknowledged the time needed to ensure high quality research outputs. The Working Party highlighted the desirability of concerted global action to coordinate research activity and formalised data gathering and sharing in the event of future pandemics caused by novel diseases. The Working Party acknowledged that some of the areas recommended for future research might already have been addressed in primary studies or systematic reviews published after the searches for the evidence review had been completed. Although the Working Party had considered updating the review to take account of more recently published evidence, the rate at which additional evidence was being published prohibited such an approach. For example, rerunning the MEDLINE and Embase searches in April 2021 indicated that approximately 20,000 further articles would need to be considered; it was, therefore, not feasible to undertake a timely and systematic update of the review using the original search terms. The Working Party emphasised that the research recommendations were intended to build on the evidence review and allow the guidance to be refined or extended, preferably with reference to evidence of higher quality and allowing more focused or nuanced consideration of SARS-CoV-2 transmission dynamics. By November 2021, rerunning the MEDLINE and Embase searches resulted in an additional 30,000 articles, which when filtered to select records containing the phrase ‘systematic review’ in the title, abstract or keywords identified nearly 600 articles. Among these systematic reviews, a handful investigated relative transmissibility of presymptomatic, asymptomatic and symptomatic infections [61–69]; however, none evaluated the impact of new variants of SARS-CoV-2 or the implementation of immunisation programmes. Indeed, most relied on literature searches conducted in a similar timescale to those of the Working Party. None of the published systematic reviews evaluated transmissibility of SARS-CoV-2 in the post-symptomatic period. The Working Party therefore concluded that no published evidence syntheses were available at the time to prompt reconsideration of the recommendations that had been formulated previously.

The Working Party noted that evidence included in the review suggested that using PPE (such as face masks or coverings) reduced the risk of transmission of SARS-CoV-2 by people with presymptomatic or asymptomatic infection. The current evidence review was not designed to explore this systematically, whereas the first of the pair of guidance articles [3] includes recommendations regarding appropriate PPE in various circumstances. The Working Party also noted that in an investigation exploring the possibility of asymptomatic transmission, hospital quarantine of the index case involved the index case and other patients and visitors wearing masks except when eating or drinking [14]. The Working Party recognised the removal of masks to allow eating and drinking as being increasingly important in nosocomial outbreaks of COVID-19, and this could have implications for activities in the community such as visiting restaurants.

#### Recommendations

Be aware that:

- people without noticeable symptoms may transmit the SARS-CoV-2 virus to other people
- transmission of SARS-CoV-2 from people without symptoms may occur in all settings in which people are in close proximity
- however, it is likely that the risk of transmission of SARS-CoV-2 is greater from people who have symptoms compared with those who do not.
  Even in the absence of symptoms, adhere to legislation and guidance regarding measures to reduce the risk of transmission of SARS-CoV-2 (such as social distancing, hand hygiene, use of personal protective equipment and ventilation of enclosed spaces).
  Be aware that the future transmissibility of SARS-CoV-2 from people carrying the virus without symptoms might depend on the:
- nature of further waves or outbreaks of COVID-19
- emergence and circulation of SARS-CoV-2 variants of concern
- potential for people who have had COVID-19 previously to be reinfected
- effectiveness of available vaccines, including the longevity of immunity they confer.

Be aware that it is not yet known to what extent or for how long people recovering from acute infection can transmit the SARS-CoV-2 virus to other people.

## Conclusions

Based on the evidence review, which included research published to the end of May 2020, the Working Party considered presymptomatic transmission of SARS-CoV-2 to be confirmed, and asymptomatic transmission to be probable. The evidence for these forms of transmission was sufficient for the Working Party to formulate several strong recommendations with the intention of raising awareness in health and care settings of the potential for transmission in the absence of symptoms. The recommendations were intended to reinforce existing legislation and guidance specifying measures for reducing the risk of transmission from people who have no noticeable symptoms. The Working Party formulated recommendations for future research to address areas of uncertainty, such as the relative transmissibility of presymptomatic, asymptomatic and symptomatic infections, the period of infectivity in people without symptoms, and the possibility of transmission in the post-symptomatic period. The Working Party emphasised the importance of good quality design, analysis and reporting of research studies even in pandemic situations. The Working Party also highlighted the desirability of concerted action to coordinate research activity and share outputs effectively.

## Further research

The rationale for the following research recommendations is presented in "Rationale for recommendations" section.

## Research recommendations

What is the relative transmissibility of SARS-CoV-2 from people with presymptomatic, asymptomatic and symptomatic infection, and how does transmission correlate with quantification of viral shedding?

How long after acquiring SARS-CoV-2 do people without symptoms become infectious and how long does infectivity last?

To what extent or for how long can people who have acquired SARS-CoV-2 and are post-symptomatic transmit the virus to other people?

What are the long-term consequences of lung damage associated with SARS-CoV-2 infection in people who do not report symptoms?

What impact do reinfection, variants of concern, and immunisation programmes have on transmission of SARS-CoV-2?

## Supplementary Information

**Additional file 1.** Working Party Report appendices.

## Data Availability

All data generated or analysed during this study are included in this published article and its supplementary information files.
