# Supplementary material for: Presymptomatic, asymptomatic and post-symptomatic transmission of SARS-CoV-2: joint British Infection Association (BIA), Healthcare Infection Society (HIS), Infection Prevention Society (IPS) and Royal College of Pathologists (RCPath) guidance
Source: BMC Infect Dis. 2022 May 12;22:453. doi: 10.1186/s12879-022-07440-0 (PMC9096060; doi:10.1186/s12879-022-07440-0)
Supplement: Supplementary file 1 — Additional file 1. Working Party Report appendices. [file 12879_2022_7440_MOESM1_ESM.docx]

## **Appendix A: Glossary**

**Asymptomatic:** never having (noticeable) symptoms.

**Carriage:** having acquired a microorganism such as a virus without becoming unwell, but with potential to transmit the microorganism to another person.

**Cluster/outbreak investigation:** a study in which a cluster (group of people in close proximity and having a condition of interest) or an outbreak of disease is investigated.

**Comparative epidemiological study:** a study in which health outcomes are compared between different groups of people, for example, a group exposed to people with presymptomatic SARS-CoV-2 infection, and another group exposed to people with symptomatic SARS-CoV-2 infection.

**Confidence interval (CI)** or **credible interval (CrI):** a statistical measure of precision associated with a reported effect.

**Coronavirus disease 2019 (COVID-19):** a respiratory disease caused by infection with SARS-CoV-2, which was first identified in December 2019.

**Critical appraisal:** a structured assessment of the strengths and limitations of a research study.

**Economic analysis:** an assessment of the cost effectiveness (value for money) of a particular aspect of health care that involves comparisons of costs and consequences of different approaches to care.

**Grading of Recommendations Assessment, Development and Evaluation (GRADE):** a structured assessment of the quality of research evidence taking account of factors such as study design, risk of bias, inconsistency and indirectness, and an associated framework for developing recommendations for practice; the quality of the evidence may be downgraded to take account of identified limitations.

**Index case:** the first person to have acquired a microorganism of interest, or developed an associated disease, and a potential source of transmission to other people.

**Mathematical modelling study:** a study in which mathematical concepts and methods are used to explain or predict the course of a disease, typically at population (rather than individual) level and often in terms of a sequence of health states, such as susceptible, exposed, infected, and recovered; may be used to explore outcomes under different assumptions or scenarios.

**Nosocomial:** associated with health care.

**Pandemic:** an epidemic that occurs over a very large geographical area, typically involving more than one country and potentially worldwide.

**Pauci-symptomatic:** having few (or mild) symptoms.

**Polymerase chain reaction (PCR):** a laboratory technique used to identify microorganisms that cause disease; reverse transcription PCR (RT-PCR) can be used to detect SARS-CoV-2 ribonucleic acid (RNA).

**Population–exposure–comparator–outcome (PECO) framework:** an approach used to classify key characteristics of epidemiological research questions and studies.

**Post-symptomatic:** no longer having symptoms but having had them in the past.

**Presymptomatic:** not yet having symptoms but having them in the future.

**Relative effect:** a statistical measure comparing health outcomes in different groups of people, for example the likelihood of SARS-CoV-2 infection in a group exposed to people with presymptomatic SARS-CoV-2 infection and another group exposed to people with symptomatic SARS-CoV-2 infection; typically expressed as an odds ratio (OR) or risk ratio (RR), with a value of 1 meaning that the outcome is equally likely in both groups and a value greater (less than) 1 meaning that the outcome is more (less) likely in the first group than in the second group.

**Ribonucleic acid (RNA):** a molecule containing a small amount of genetic material; in the case of SARS-CoV-2 the entire genetic information needed for the virus to replicate is contained within its RNA.

**Serological testing:** antibody testing, for example, using enzyme-linked immunosorbent assay (ELISA) or virus neutralisation test (VNT).

**Severe acute respiratory syndrome coronavirus 2 (SARS-CoV-2):** the virus that causes COVID-19.

**Symptom status:** whether an index case is presymptomatic, asymptomatic, post-symptomatic or symptomatic during the exposure period of their close contacts.

**Transmission dynamics:** the characteristics involved in transmission of a disease, including changes in transmission over time; for example, the relative transmissibility of presymptomatic and symptomatic SARS-Cov-2 infections.

**Variant:** a version of a virus arising from replication and containing one or more mutations compared to the original virus; variants may be classified as being of interest or concern depending on the extent to which they pose challenges for healthcare systems.

**Viral load:** the number of viral particles in a sample obtained from a person or environment; the amount of virus present determines the likelihood that another person will acquire the virus.

## **Appendix B: Guideline development and conflicts of interest**

The need for guidance in this area was agreed between BIA, HIS, IPS, RCPath and the British Society for Antimicrobial Chemotherapy (BSAC) at the beginning of the first wave of COVID-19 affecting the UK in March 2020. The need arose from the concern of healthcare workers reporting a lack of evidence on which to base practice. Further meetings between the participating organisations confirmed the need to establish a COVID-19 Rapid Guidance Working Party. Members of the Working Party were chosen to reflect the range of stakeholders. Feedback from members of the participating organisations was used to direct the development of review questions. The final structure of the review questions (in PECO format) was agreed collectively during subsequent videoconference meetings. After initial questions had been agreed, if the need for additional questions arose, these were considered for inclusion at subsequent meetings. No payment was made to anyone involved in developing the guidance.

All Working Party members were required to complete conflict of interest forms during the development of the guidance and until the point of completion. In the event of a potential conflict being identified, the Working Party agreed that the member should not contribute to the section affected. No material conflicts of interest were identified during the development of this guidance.

## **Appendix C: Review question and literature searches**

Review question: *What is the evidence that presymptomatic, asymptomatic and post-symptomatic people with beta-coronavirus can transmit the virus to other people?*

This review was registered on the PROSPERO international prospective register of systematic reviews (registration identifier CRD42020188880).

PECO framework:

- population – any person at risk of exposure to beta-coronavirus in a community or healthcare setting, excluding vertical transmission (that is, direct transmission from a woman to her baby before, during or shortly after the birth) and transmission as a result of blood transfusion based on the first article in the pair of guidance documents concluding that these routes of transmission are unlikely;^[3]^ extrapolation from SARS-CoV or MERS-CoV to SARS-CoV-2 to be considered if needed
- exposure – exposure to beta-coronavirus via a person with presymptomatic, asymptomatic or post-symptomatic infection
- comparison – no comparison group needed (but comparative studies eligible for inclusion, for example, those comparing transmission rates according to the index case’s symptom status during exposure of their close contacts)
- outcomes – evidence of transmission to another person; the main outcome of interest was incidence of transmission to another person of disease caused by beta-coronavirus with cases either being confirmed by laboratory or radiological findings or displaying symptoms suggesting disease.

For SARS-C0V-2 evidence, no restrictions were placed on study designs to be included (thus mathematical modelling studies and letters were eligible for inclusion provided the publication contained primary data related to presymptomatic, asymptomatic or post-symptomatic transmission). No restrictions were placed on language of publication; articles published in languages other than English were translated using Google Translate (available at <https://translate.google.co.uk/>).

For other beta-coronaviruses, study designs were to be limited to peer-reviewed reports of controlled trials, interrupted time series, controlled before-and-after studies, cohort studies and outbreak reports, and mathematical modelling studies that contained primary data. As noted above, the intention had been to consider evidence for SARS-CoV and MERS-CoV if needed, but the volume of evidence identified for SARS-CoV-2 was sufficient to negate the need for additional searches for other beta-coronaviruses.

**Table C.1** Search strategy for SARS-CoV-2 evidence, MEDLINE and Embase

Database: Embase <1974 to 2020 May 29>, Ovid MEDLINE(R) ALL <1946 to May 29, 2020>

Search Strategy:

--------------------------------------------------------------------------------

1 coronavirus.mp. or exp Coronavirinae/ (68155)

2 exp SARS coronavirus/ or coronovirus.mp. or exp Coronavirus infection/ (35880)

3 severe acute respiratory syndrome.mp. or severe acute respiratory syndrome/ or respiratory distress syndrome/ (41899)

4 Severe acute respiratory syndrome coronavirus 2.mp. (8507)

5 SARS-CoV-2.mp. (9133)

6 SARSCoV-2.mp. (59)

7 SARSCov2.mp. (3)

8 SARS-Cov2.mp. (460)

9 SARS-Cov-19.mp. (11)

10 COVID19.mp. (409)

11 nCoV-2019.mp. or SARS-related coronavirus/ (3537)

12 COVID-19.mp. (30054)

13 2019-nCoV.mp. (1439)

14 2019nCoV.mp. or Betacoronavirus/ (4978)

15 HCoV-19.mp. (14)

16 novel coronavirus.mp. (5192)

17 wuhan virus.mp. (10)

18 wuhan coronavirus.mp. (32)

19 hubei virus.mp. (0)

20 hubei coronavirus.mp. (0)

21 huanan virus.mp. (0)

22 huanan coronavirus.mp. (0)

23 wuhan pneumonia.mp. (20)

24 hubei pneumonia.mp. (0)

25 huanan pneumonia.mp. (0)

26 CoV.mp. (23003)

27 2019 novel.mp. (1438)

28 Ncov.mp. (1523)

29 n-cov.mp. (7)

30 Seafood market pneumonia.mp. (5)

31 1 or 2 or 3 or 4 or 5 or 6 or 7 or 8 or 9 or 10 or 11 or 12 or 13 or 14 or 15 or 16 or 17 or 18 or 19 or 20 or 21 or 22 or 23 or 24 or 25 or 26 or 27 or 28 or 29 or 30 (103896)

32 Asymptomatic Infections/ or Asymptomatic Diseases/ or asymptom$.mp. (385437)

33 pre-symptom$.mp. (3686)

34 presymptom$.mp. (9640)

35 post-symptom$.mp. (407)

36 postsymptom$.mp. (115)

37 pauci-symptom$.mp. (245)

38 paucisymptom$.mp. (577)

39 symptom$.mp. (2959134)

40 sub-clinical.mp. (7247)

41 subclinical.mp. (104806)

42 silent carrier.mp. (105)

43 "Signs and Symptoms"/ (11809)

44 "Signs and Symptoms, Respiratory"/ (11402)

45 "Pathological Conditions, Signs and Symptoms"/ (11402)

46 sign?.mp. (925579)

47 (indicat$ or presentation).mp. (8172942)

48 ((clinical or physical or presenting) adj3 (feature? or finding? or factor? or manifest$ or aspect? or character?)).mp. (1497887)

49 unrecogni?ed.mp. (68558)

50 32 or 33 or 34 or 35 or 36 or 37 or 38 or 39 or 40 or 41 or 42 or 43 or 44 or 45 or 46 or 47 or 48 or 49 (12329286)

51 transmission.mp. or virus transmission/ (1126930)

52 transmissibility.mp. (7407)

53 spread.mp. (360416)

54 *basic reproduction number/ (441)

55 route.mp. (328545)

56 mode.mp. (626428)

57 cross infection/ or cross-infection.mp. or crossinfection.mp. (78473)

58 expos$.mp. (2878955)

59 viral load.mp. or virus load/ (131041)

60 infectivity.mp. (60378)

61 infectiousness.mp. (3008)

62 51 or 52 or 53 or 54 or 55 or 56 or 57 or 58 or 59 or 60 or 61 (5240183)

63 infection control.mp. or Infection Control/ (134346)

64 Disease Transmission, Infectious/ (107205)

65 Infectious Disease Transmission, Patient-to-Professional/ (101926)

66 Infectious Disease Transmission, Professional-to-Patient/ (99715)

67 Community-Acquired Infections/tm [Transmission] (419)

68 patient-patient.mp. (1812)

69 patient-to-patient.mp. (21944)

70 professional-professional.mp. (60)

71 professional-to-professional.mp. (693)

72 human-human.mp. (1080)

73 human-to-human.mp. (11543)

74 63 or 64 or 65 or 66 or 67 or 68 or 69 or 70 or 71 or 72 or 73 (268327)

75 62 or 74 (5345224)

76 31 and 50 and 75 (8066)

77 limit 76 to yr="2020 -Current" (2742)

78 limit 77 to (animals and animal studies) [Limit not valid in Ovid MEDLINE(R),Ovid MEDLINE(R) Daily Update,Ovid MEDLINE(R) In-Process,Ovid MEDLINE(R) Publisher; records were retained] (55)

79 *in vitro study/ (34528)

80 77 not 78 (2687)

81 80 not 79 (2686)

82 remove duplicates from 81 (1650)

## **Appendix D: Study selection flowchart**

**Figure D.1** Study selection flowchart

## **Appendix E: Excluded studies table**

**Table E.1** List of excluded studies

| **Citation** | **Reason for exclusion** |
| --- | --- |
| Abbott S; Hellewell J; Munday J; CMMID nCoV working group; Funk S. The transmissibility of novel Coronavirus in the early stages of the 2019-20 outbreak in Wuhan: Exploring initial point-source exposure sizes and durations using scenario analysis. Wellcome Open Research. 5:17, 2020. | No primary data related to person-to-person transmission during asymptomatic, pre-symptomatic or post-symptomatic phases; this was a modelling study that did not incorporate asymptomatic/presymptomatic transmission |
| Abduljalil JM; Abduljalil BM. Epidemiology, genome, and clinical features of the pandemic SARS-CoV-2: a recent view. [Review] New Microbes & New Infections. 35:100672, 2020 May. | No primary data related to person-to-person transmission during asymptomatic, pre-symptomatic or post-symptomatic phases |
| Adam D. Special report: The simulations driving the world's response to COVID-19. Nature. 580(7803):316-318, 2020 04. | No primary data related to person-to-person transmission during asymptomatic, pre-symptomatic or post-symptomatic phases |
| Adegboye OA; Adekunle AI; Gayawan E. Early Transmission Dynamics of Novel Coronavirus (COVID-19) in Nigeria. International Journal of Environmental Research & Public Health [Electronic Resource]. 17(9), 2020 04 28. | No primary data related to person-to-person transmission during asymptomatic, pre-symptomatic or post-symptomatic phases; this was a modelling study that did not distinguish between asymptomatic/presymptomatic and symptomatic transmission |
| Advani SD; Smith BA; Lewis SS; Anderson DJ; Sexton DJ. Universal masking in hospitals in the COVID-19 era: Is it time to consider shielding?. Infection Control & Hospital Epidemiology. 1-2, 2020 Apr 29. | No primary data related to person-to-person transmission during asymptomatic, pre-symptomatic or post-symptomatic phases |
| Aguilar JB, Faust JS, Westafer, LM, Gutierrez JB Investigating the impact of asymptomatic carriers on COVID-19 transmission medRxiv, https://doi.org/10.1101/2020.03.18.20037994 | No primary data related to person-to-person transmission during asymptomatic, pre-symptomatic or post-symptomatic phases; this was a modelling study that incorporated asymptomatic/presymptomatic transmission but only through scenarios based on assumed transmissibility and the impact on e.g. the reproduction number |
| Aleta A; Moreno Y. Evaluation of the potential incidence of COVID-19 and effectiveness of containment measures in Spain: a data-driven approach. BMC Medicine. 18(1):157, 2020 05 27. | No primary data related to person-to-person transmission during asymptomatic, pre-symptomatic or post-symptomatic phases; this was a modelling study that acknowledged the possibility of asymptomatic/pre-symptomatic transmission but did not distinguish between these and symptomatic transmission |
| Alonso Diaz C; Lopez Maestro M; Moral Pumarega MT; Flores Anton B; Pallas Alonso CR. [First case of neonatal infection due to SARS-CoV-2 in Spain]. [Spanish] Primer caso de infeccion neonatal por SARS-CoV-2 en Espana. <Primer caso de infeccion neonatal por SARS-CoV-2 en Espana.> Anales de Pediatria. 92(4):237-238, 2020 Apr. | Incomplete exposure history; authors discuss the possibility of horizontal rather than vertical transmission; baby and woman were together when the woman developed symptoms; the baby became symptomatic later, but had been separated from the woman immediately after the birth and again when the woman developed symptoms |
| Al-Tawfiq J.A. Asymptomatic coronavirus infection: MERS-CoV and SARS-CoV-2 (COVID-19). Travel Medicine and Infectious Disease. (no pagination), 2020. Article Number: 101608. Date of Publication: 2020. | No primary data related to person-to-person transmission during asymptomatic, pre-symptomatic or post-symptomatic phases |
| an der Heiden M, Buchholz U [Modeling example scenarios of the SARS-CoV-2 epidemic 2020 in Germany]. [German] Modellierung von Beispielszenarien der SARS-CoV-2-Epidemie 2020 in Deutschland. <Modellierung von Beispielszenarien der SARS-CoV-2-Epidemie 2020 in Deutschland.> Publication server of Robert Koch Institute, https://edoc.rki.de/ | No primary data related to person-to-person transmission during asymptomatic, pre-symptomatic or post-symptomatic phases; this was a modelling study that did not incorporate asymptomatic/presymptomatic transmission |
| Anastassopoulou C; Russo L; Tsakris A; Siettos C. Data-based analysis, modelling and forecasting of the COVID-19 outbreak. PLoS ONE [Electronic Resource]. 15(3):e0230405, 2020. | No primary data related to person-to-person transmission during asymptomatic, pre-symptomatic or post-symptomatic phases; this was a modelling study that incorporated asymptomatic/presymptomatic states but only through scenarios based on assumed proportions of asymptomatic/presymptomatic infections and the impact on e.g. the basic reproduction number |
| Anderson EL; Turnham P; Griffin JR; Clarke CC. Consideration of the Aerosol Transmission for COVID-19 and Public Health. Risk Analysis. 40(5):902-907, 2020 05. | No primary data related to person-to-person transmission during asymptomatic, pre-symptomatic or post-symptomatic phases |
| Anelli F., Leoni G., Monaco R., Nume C., Rossi R.C., Marinoni G., Spata G., De Giorgi D., Peccarisi L., Miani A., Burgio E., Gentile I., Colao A., Triassi M., Piscitelli P. Italian doctors call for protecting healthcare workers and boosting community surveillance during covid-19 outbreak. The BMJ. 368 (no pagination), 2020. Article Number: m1254. Date of Publication: 26 Mar 2020. | No primary data related to person-to-person transmission during asymptomatic, pre-symptomatic or post-symptomatic phases |
| Anonymous Correction to Feasibility of controlling COVID-19 outbreaks by isolation of cases and contacts (The Lancet Global Health (2020) 8(4) (e488-e496), (S2214109X20300747), (10.1016/S2214-109X(20)30074-7)). The Lancet Global Health. (no pagination), 2020. Date of Publication: 2020. | This is a correction to Hellewell 2020^[70]^ which was excluded after considering full text |
| Anonymous Correction to Lancet Infect Dis 2020; published online March 23. https://doi.org/10.1016/S1473-3099(20)30162 (The Lancet Infectious Diseases, (S1473309920301626), (10.1016/S1473-3099(20)30162-6)). The Lancet Infectious Diseases. 20 (5) (pp e79), 2020. Date of Publication: May 2020. | This is a correction to Koo 2020^[71]^ which was excluded after considering full text |
| Anonymous Corrigendum: Epidemiologic and clinical characteristics of 26 cases of COVID-19 arising from patient-to-patient transmission in Liaocheng, China (Clin Epidemiol., (2020) 12, (387-391), 10.2147/CLEP.S249903). Clinical Epidemiology. 12 (pp 403), 2020. Date of Publication: 2020. | Full text not retrieved because this is a correction to Wang 2020^[72]^ which was excluded after considering full text |
| Anonymous Erratum to "Initial Investigation of Transmission of COVID-19 Among Crew Members During Quarantine of a Cruise Ship - Yokohama, Japan, February 2020." MMWR. Morbidity and mortality weekly report. | This is a correction to Kakimoto 2020^[73]^ which was excluded after considering full text |
| Arino J; Portet S. A simple model for COVID-19. Infectious Disease Modelling. 5:309-315, 2020. | No primary data related to person-to-person transmission during asymptomatic, pre-symptomatic or post-symptomatic phases; this was a modelling study that incorporated asymptomatic/presymptomatic transmission |
| Arons MM; Hatfield KM; Reddy SC; Kimball A; James A; Jacobs JR; Taylor J; Spicer K; Bardossy AC; Oakley LP; Tanwar S; Dyal JW; Harney J; Chisty Z; Bell JM; Methner M; Paul P; Carlson CM; McLaughlin HP; Thornburg N; Tong S; Tamin A; Tao Y; Uehara A; Harcourt J; Clark S; Brostrom-Smith C; Page LC; Kay M; Lewis J; Montgomery P; Stone ND; Clark TA; Honein MA; Duchin JS; Jernigan JA; Public Health-Seattle and King County and CDC COVID-19 Investigation Team. Presymptomatic SARS-CoV-2 Infections and Transmission in a Skilled Nursing Facility. New England Journal of Medicine. 382(22):2081-2090, 2020 05 28. | No primary data related to person-to-person transmission during asymptomatic, pre-symptomatic or post-symptomatic phases; same study/setting as Kimball 2020^[74]^ |
| Ash C. Tracing infection from mobility data. Science. 368 (6490) (pp 484-485), 2020. Date of Publication: 01 May 2020. | No primary data related to person-to-person transmission during asymptomatic, pre-symptomatic or post-symptomatic phases |
| Bae JM. A Chinese Case of COVID-19 Did Not Show Infectivity During the Incubation Period: Based on an Epidemiological Survey. Journal of Preventive Medicine & Public Health / Yebang Uihakhoe Chi. 2020 Mar 02. | Duplicate of Bae 2020^[4]^ |
| Bai S, Wang J, Zhou Y, Yu D, Gao X, Li L, Yang F [Analysis of the first family-associated epidemic situation of new coronavirus pneumonia in Gansu Province]. [Chinese] Chinese Journal of Preventive Medicine, 2020,54: Online pre-publishing . DOI: 10.3760/cma.j.cn112150-20200204-00065 | Duplicate of Bai 2020a^[75]^ |
| Bai SL; Wang JY; Zhou YQ; Yu DS; Gao XM; Li LL; Yang F. [Analysis of the first cluster of cases in a family of novel coronavirus pneumonia in Gansu Province]. [Chinese] Chung-Hua Yu Fang i Hsueh Tsa Chih [Chinese Journal of Preventive Medicine]. 54(0):E005, 2020 Feb 17. | No primary data related to person-to-person transmission during asymptomatic, pre-symptomatic or post-symptomatic phases; the index patient had some symptomatic contact with both of the other patients and exposure histories were not reported fully |
| Baraniuk C. What the Diamond Princess taught the world about covid-19. The BMJ. 369 (no pagination), 2020. Article Number: m1632. Date of Publication: 27 Apr 2020. | No primary data related to person-to-person transmission during asymptomatic, pre-symptomatic or post-symptomatic phases |
| Bernard Stoecklin S; Rolland P; Silue Y; Mailles A; Campese C; Simondon A; Mechain M; Meurice L; Nguyen M; Bassi C; Yamani E; Behillil S; Ismael S; Nguyen D; Malvy D; Lescure FX; Georges S; Lazarus C; Tabai A; Stempfelet M; Enouf V; Coignard B; Levy-Bruhl D; Investigation Team. First cases of coronavirus disease 2019 (COVID-19) in France: surveillance, investigations and control measures, January 2020. Euro Surveillance: Bulletin Europeen sur les Maladies Transmissibles = European Communicable Disease Bulletin. 25(6), 2020 02. | No primary data related to person-to-person transmission during asymptomatic, pre-symptomatic or post-symptomatic phases; one index patient had symptomatic contact with at least some people traced as contacts; another index patient had symptomatic contact with a least some people traced as contacts and possible co-exposure with his wife at the time the infection was acquired; exposure histories were not reported fully |
| Bi Q; Wu Y; Mei S; Ye C; Zou X; Zhang Z; Liu X; Wei L; Truelove SA; Zhang T; Gao W; Cheng C; Tang X; Wu X; Wu Y; Sun B; Huang S; Sun Y; Zhang J; Ma T; Lessler J; Feng T. Epidemiology and transmission of COVID-19 in 391 cases and 1286 of their close contacts in Shenzhen, China: a retrospective cohort study. The Lancet Infectious Diseases. 2020 Apr 27. | No primary data related to person-to-person transmission during asymptomatic, pre-symptomatic or post-symptomatic phases |
| Brandstetter S; Roth S; Harner S; Buntrock-Dopke H; Toncheva A; Borchers N; Gruber R; Ambrosch A; Kabesch M. Symptoms and immunoglobulin development in hospital staff exposed to a SARS-CoV-2 outbreak. Pediatric Allergy & Immunology. 2020 May 15. | No primary data related to person-to-person transmission during asymptomatic, pre-symptomatic or post-symptomatic phases |
| Breslin N; Baptiste C; Gyamfi-Bannerman C; Miller R; Martinez R; Bernstein K; Ring L; Landau R; Purisch S; Friedman AM; Fuchs K; Sutton D; Andrikopoulou M; Rupley D; Sheen JJ; Aubey J; Zork N; Moroz L; Mourad M; Wapner R; Simpson LL; D'Alton ME; Goffman D. COVID-19 infection among asymptomatic and symptomatic pregnant women: Two weeks of confirmed presentations to an affiliated pair of New York City hospitals. American Journal of Obstetrics & Gynecology MFM. 100118, 2020 Apr 09. | No primary data related to person-to-person transmission during asymptomatic, pre-symptomatic or post-symptomatic phases; neonatal outcomes reported but exposure history not reported fully |
| Burke R.M., Midgley C.M., Dratch A., Fenstersheib M., Haupt T., Holshue M., Ghinai I., Jarashow M.C., Lo J., McPherson T.D., Rudman S., Scott S., Hall A.J., Fry A.M., Rolfes M.A. Active Monitoring of Persons Exposed to Patients with Confirmed COVID-19 - United States, January-February 2020. MMWR. Morbidity and mortality weekly report. 69 (9) (pp 245-246), 2020. Date of Publication: 06 Mar 2020. | No primary data related to person-to-person transmission during asymptomatic, pre-symptomatic or post-symptomatic phases |
| Cabore JW; Karamagi HC; Kipruto H; Asamani JA; Droti B; Seydi ABW; Titi-Ofei R; Impouma B; Yao M; Yoti Z; Zawaira F; Tumusiime P; Talisuna A; Kasolo FC; Moeti MR. The potential effects of widespread community transmission of SARS-CoV-2 infection in the World Health Organization African Region: a predictive model. BMJ Global Health. 5(5), 2020 May. | No primary data related to person-to-person transmission during asymptomatic, pre-symptomatic or post-symptomatic phases; this was a modelling study that incorporated asymptomatic/presymptomatic states but did not distinguish between asymptomatic/presymptomatic and symptomatic transmission |
| Calafiore GC, Novara C, Possieri C A modified SIR model for the COVID-19 contagion in Italy arxiv, https://arxiv.org/abs/2003.14391 | No primary data related to person-to-person transmission during asymptomatic, pre-symptomatic or post-symptomatic phases; this was a modelling study that acknowledged the possibility of asymptomatic/presymptomatic infection but it did not incorporate asymptomatic/presymptomatic transmission |
| Canova V; Lederer Schlapfer H; Piso RJ; Droll A; Fenner L; Hoffmann T; Hoffmann M. Transmission risk of SARS-CoV-2 to healthcare workers -observational results of a primary care hospital contact tracing. Swiss Medical Weekly. 150:w20257, 2020 04 20. | No primary data related to person-to-person transmission during asymptomatic, pre-symptomatic or post-symptomatic phases; transmission to health care workers occurred during index patient's symptomatic phase |
| Cao Q., Chen Y.-C., Chen C.-L., Chiu C.-H. SARS-CoV-2 infection in children: Transmission dynamics and clinical characteristics. Journal of the Formosan Medical Association. 119 (3) (pp 670-673), 2020. Date of Publication: March 2020. | No primary data related to person-to-person transmission during asymptomatic, pre-symptomatic or post-symptomatic phases |
| CDC COVID-19 Response Team. Characteristics of Health Care Personnel with COVID-19 - United States, February 12-April 9, 2020. MMWR - Morbidity & Mortality Weekly Report. 69(15):477-481, 2020 Apr 17. | No primary data related to person-to-person transmission during asymptomatic, pre-symptomatic or post-symptomatic phases |
| Chang D., Lin M., Wei L., Xie L., Zhu G., Dela Cruz C.S., Sharma L. Epidemiologic and Clinical Characteristics of Novel Coronavirus Infections Involving 13 Patients Outside Wuhan, China. JAMA - Journal of the American Medical Association. 323 (11) (pp 1092-1093), 2020. Date of Publication: 17 Mar 2020. | No primary data related to person-to-person transmission during asymptomatic, pre-symptomatic or post-symptomatic phases |
| Chang D., Xu H., Rebaza A., Sharma L., Dela Cruz C.S. Protecting health-care workers from subclinical coronavirus infection. The Lancet Respiratory Medicine. 8 (3) (pp e13), 2020. Date of Publication: March 2020. | No primary data related to person-to-person transmission during asymptomatic, pre-symptomatic or post-symptomatic phases |
| Chang SL, Harding N, Zachreson C, Cliff OM, Prokopenko M Modelling transmission and control of the COVID-19 pandemic in Australia arxiv, https://arxiv.org/abs/2003.10218 | No primary data related to person-to-person transmission during asymptomatic, pre-symptomatic or post-symptomatic phases; this was a modelling study that incorporated asymptomatic/presymptomatic transmission but only through scenarios based on assumed transmissibility and the impact on e.g. disease incidence |
| Chen CM; Jyan HW; Chien SC; Jen HH; Hsu CY; Lee PC; Lee CF; Yang YT; Chen MY; Chen LS; Chen HH; Chan CC. Containing COVID-19 Among 627,386 Persons in Contact With the Diamond Princess Cruise Ship Passengers Who Disembarked in Taiwan: Big Data Analytics. Journal of Medical Internet Research. 22(5):e19540, 2020 05 05. | No primary data related to person-to-person transmission during asymptomatic, pre-symptomatic or post-symptomatic phases; this study used automated contact tracing and notification to undertake quarantine but the symptom status of potential index patients was unknown |
| Chen J. Pathogenicity and transmissibility of 2019-nCoV A quick overview and comparison with other emerging viruses Microbes and Infection 22 (2020) 69e7170 | No primary data related to person-to-person transmission during asymptomatic, pre-symptomatic or post-symptomatic phases; this was a modelling study that incorporated asymptomatic/presymptomatic transmission but only through scenarios based on assumed transmissibility and the impact on e.g. the number of new cases |
| Chen P; Zhang Y; Wen Y; Guo J; Jia J; Ma Y; Xu Y. Epidemiological and clinical characteristics of 136 cases of COVID-19 in main district of Chongqing. Journal of the Formosan Medical Association. 2020 Apr 29. | No primary data related to person-to-person transmission during asymptomatic, pre-symptomatic or post-symptomatic phases |
| Chen S., Yang J., Yang W., Wang C., Barnighausen T. COVID-19 control in China during mass population movements at New Year. The Lancet. 395 (10226) (pp 764-766), 2020. Date of Publication: 7 - 13 March 2020. | No primary data related to person-to-person transmission during asymptomatic, pre-symptomatic or post-symptomatic phases |
| Chen S; Huang B; Luo DJ; Li X; Yang F; Zhao Y; Nie X; Huang BX. [Pregnancy with new coronavirus infection: clinical characteristics and placental pathological analysis of three cases]. [Chinese] Chung-Hua Ping Li Hsueh Tsa Chih - Chinese Journal of Pathology. 49(5):418-423, 2020 May 08. | No primary data related to person-to-person transmission during asymptomatic, pre-symptomatic or post-symptomatic phases |
| Chen T.-M., Rui J., Wang Q.-P., Zhao Z.-Y., Cui J.-A., Yin L. A mathematical model for simulating the phase-based transmissibility of a novel coronavirus. Infectious Diseases of Poverty. 9 (1) (no pagination), 2020. Article Number: 24. Date of Publication: 28 Feb 2020. | No primary data related to person-to-person transmission during asymptomatic, pre-symptomatic or post-symptomatic phases; this was a modelling study that incorporated asymptomatic/presymptomatic transmission but only through scenarios based on assumed transmissibility and the impact on e.g. the number of deaths |
| Cheng X; Liu J; Li N; Nisenbaum E; Sun Q; Chen B; Casiano R; Weed D; Telischi F; Denneny JC 3rd; Liu X; Shu Y. Otolaryngology Providers Must Be Alert for Patients with Mild and Asymptomatic COVID-19. Otolaryngology - Head & Neck Surgery. 194599820920649, 2020 Apr 14. | No primary data related to person-to-person transmission during asymptomatic, pre-symptomatic or post-symptomatic phases |
| Chia P.Y., Coleman K.K., Tan Y.K., Ong S.W.X., Gum M., Lau S.K., Lim X.F., Lim A.S., Sutjipto S., Lee P.H., Son T.T., Young B.E., Milton D.K., Gray G.C., Schuster S., Barkham T., De P.P., Vasoo S., Chan M., Ang B.S.P., Tan B.H., Leo Y.-S., Ng O.-T., Wong M.S.Y., Marimuthu K., Lye D.C., Lim P.L., Lee C.C., Ling L.M., Lee L., Lee T.H., Wong C.S., Sadarangani S., Lin R.J., Ng D.H.L., Sadasiv M., Yeo T.W., Choy C.Y., Tan G.S.E., Dimatatac F., Santos I.F., Go C.J., Chan Y.K., Tay J.Y., Tan J.Y.-L., Pandit N., Ho B.C.H., Mendis S., Chen Y.Y.C., Abdad M.Y., Moses D. Detection of air and surface contamination by SARS-CoV-2 in hospital rooms of infected patients. Nature Communications. 11 (1) (no pagination), 2020. Article Number: 2800. Date of Publication: 01 Dec 2020. | No primary data related to person-to-person transmission during asymptomatic, pre-symptomatic or post-symptomatic phases |
| Chinazzi M; Davis JT; Ajelli M; Gioannini C; Litvinova M; Merler S; Pastore Y Piontti A; Mu K; Rossi L; Sun K; Viboud C; Xiong X; Yu H; Halloran ME; Longini IM Jr; Vespignani A. The effect of travel restrictions on the spread of the 2019 novel coronavirus (COVID-19) outbreak. Science. 368(6489):395-400, 2020 04 24. | No primary data related to person-to-person transmission during asymptomatic, pre-symptomatic or post-symptomatic phases; this was a modelling study that did not incorporate asymptomatic/presymptomatic transmission |
| Chintalapudi N., Battineni G., Amenta F. COVID-19 virus outbreak forecasting of registered and recovered cases after sixty day lockdown in Italy: A data driven model approach. Journal of Microbiology, Immunology and Infection. (no pagination), 2020. Date of Publication: 2020. | No primary data related to person-to-person transmission during asymptomatic, pre-symptomatic or post-symptomatic phases; this was a modelling study that acknowledged the possibility of asymptomatic/pre-symptomatic transmission but did not distinguish between these and symptomatic transmission |
| Chintalapudi N; Battineni G; Sagaro GG; Amenta F. COVID-19 outbreak reproduction number estimations and forecasting in Marche, Italy. International Journal of Infectious Diseases. 96:327-333, 2020 May 11. | No primary data related to person-to-person transmission during asymptomatic, pre-symptomatic or post-symptomatic phases; this was a modelling study that did not incorporate asymptomatic/presymptomatic transmission |
| Cho HJ; Koo JW; Roh SK; Kim YK; Suh JS; Moon JH; Sohn SK; Baek DW. COVID-19 transmission and blood transfusion: A case report. Journal of Infection and Public Health. 2020 May 13. | No primary data related to person-to-person transmission during asymptomatic, pre-symptomatic or post-symptomatic phases |
| Choi S.-H., Kim H.W., Kang J.-M., Kim D.H., Cho E.Y. Epidemiology and clinical features of coronavirus disease 2019 in children. Pediatric Infection and Vaccine. 27 (1) (pp 11-23), 2020. Date of Publication: 2020. | No primary data related to person-to-person transmission during asymptomatic, pre-symptomatic or post-symptomatic phases |
| Choi S; Ki M. Estimating the reproductive number and the outbreak size of COVID-19 in Korea. Epidemiology and health. 42:e2020011, 2020. | No primary data related to person-to-person transmission during asymptomatic, pre-symptomatic or post-symptomatic phases; this was a modelling study that did not incorporate asymptomatic/presymptomatic transmission |
| Corman VM; Rabenau HF; Adams O; Oberle D; Funk MB; Keller-Stanislawski B; Timm J; Drosten C; Ciesek S. SARS-CoV-2 asymptomatic and symptomatic patients and risk for transfusion transmission. Transfusion. 2020 May 02. | No primary data related to person-to-person transmission during asymptomatic, pre-symptomatic or post-symptomatic phases |
| COVID-19 National Emergency Response Center, Epidemiology and Case Management Team, Korea Centers for Disease Control and Prevention. Early Epidemiological and Clinical Characteristics of 28 Cases of Coronavirus Disease in South Korea. Osong Public Health & Research Perspectives. 11(1):8-14, 2020 Feb. | No primary data related to person-to-person transmission during asymptomatic, pre-symptomatic or post-symptomatic phases; several clusters were reported but at least some of the index patients had symptomatic contact with other patients and exposure histories were not reported fully |
| Danis K; Epaulard O; Benet T; Gaymard A; Campoy S; Bothelo-Nevers E; Bouscambert-Duchamp M; Spaccaferri G; Ader F; Mailles A; Boudalaa Z; Tolsma V; Berra J; Vaux S; Forestier E; Landelle C; Fougere E; Thabuis A; Berthelot P; Veil R; Levy-Bruhl D; Chidiac C; Lina B; Coignard B; Saura C; Investigation Team. Cluster of coronavirus disease 2019 (Covid-19) in the French Alps, 2020. Clinical Infectious Diseases. 2020 Apr 11. | No primary data related to person-to-person transmission during asymptomatic, pre-symptomatic or post-symptomatic phases; the index patient had symptomatic contact with at least some of the other patients and subsequent exposure histories were not reported fully |
| Davenport MS; Bruno MA; Iyer RS; Johnson AM; Herrera R; Nicola GN; Ortiz D; Pedrosa I; Policeni B; Recht MP; Willis M; Zuley ML; Weinstein S. ACR Statement on Safe Resumption of Routine Radiology Care During the Coronavirus Disease 2019 (COVID-19) Pandemic. Journal of the American College of Radiology. 2020 May 06. | No primary data related to person-to-person transmission during asymptomatic, pre-symptomatic or post-symptomatic phases |
| de Lusignan S; Lopez Bernal J; Zambon M; Akinyemi O; Amirthalingam G; Andrews N; Borrow R; Byford R; Charlett A; Dabrera G; Ellis J; Elliot AJ; Feher M; Ferreira F; Krajenbrink E; Leach J; Linley E; Liyanage H; Okusi C; Ramsay M; Smith G; Sherlock J; Thomas N; Tripathy M; Williams J; Howsam G; Joy M; Hobbs R. Emergence of a Novel Coronavirus (COVID-19): Protocol for Extending Surveillance Used by the Royal College of General Practitioners Research and Surveillance Centre and Public Health England. JMIR Public Health and Surveillance. 6(2):e18606, 2020 04 02. | No primary data related to person-to-person transmission during asymptomatic, pre-symptomatic or post-symptomatic phases |
| Desai A.N., Patel P. Stopping the Spread of COVID-19. JAMA - Journal of the American Medical Association. 323 (15) (pp 1516), 2020. Date of Publication: 21 Apr 2020. | No primary data related to person-to-person transmission during asymptomatic, pre-symptomatic or post-symptomatic phases |
| Dexter F; Elhakim M; Loftus RW; Seering MS; Epstein RH. Strategies for daily operating room management of ambulatory surgery centers following resolution of the acute phase of the COVID-19 pandemic. Journal of Clinical Anesthesia. 64:109854, 2020 Apr 29. | No primary data related to person-to-person transmission during asymptomatic, pre-symptomatic or post-symptomatic phases |
| Diaz-Quijano FA; Rodriguez-Morales AJ; Waldman EA. Translating transmissibility measures into recommendations for coronavirus prevention. Revista de Saude Publica. 54:43, 2020. | No primary data related to person-to-person transmission during asymptomatic, pre-symptomatic or post-symptomatic phases |
| Dickens B.L., Koo J.R., Wilder-Smith A., Cook A.R. Institutional, not home-based, isolation could contain the COVID-19 outbreak. The Lancet. 395 (10236) (pp 1541-1542), 2020. Date of Publication: 16 - 22 May 2020. | No primary data related to person-to-person transmission during asymptomatic, pre-symptomatic or post-symptomatic phases; this was a modelling study that did not incorporate asymptomatic/presymptomatic transmission |
| Diop BZ; Ngom M; Pougue Biyong C; Pougue Biyong JN. The relatively young and rural population may limit the spread and severity of COVID-19 in Africa: a modelling study. BMJ Global Health. 5(5), 2020 05. | No primary data related to person-to-person transmission during asymptomatic, pre-symptomatic or post-symptomatic phases; this was a modelling study that incorporated asymptomatic/presymptomatic transmission but only through scenarios based on assumed transmissibility and the impact on e.g. the proportion of the population that would become infected |
| Dong XC; Li JM; Bai JY; Liu ZQ; Zhou PH; Gao L; Li XY; Zhang Y. [Epidemiological characteristics of confirmed COVID-19 cases in Tianjin]. [Chinese] Chung-Hua Liu Hsing Ping Hsueh Tsa Chih Chinese Journal of Epidemiology. 41(5):638-641, 2020 May 10. | No primary data related to person-to-person transmission during asymptomatic, pre-symptomatic or post-symptomatic phases |
| Donsimoni JR; Glawion R; Plachter B; Walde K. [Projecting the Spread of COVID-19 for Germany]. [German] Projektion der COVID-19-Epidemie in Deutschland. <Projektion der COVID-19-Epidemie in Deutschland.> Wirtschaftsdienst. 100(4):272-276, 2020. | No primary data related to person-to-person transmission during asymptomatic, pre-symptomatic or post-symptomatic phases; this was a modelling study that incorporated asymptomatic/presymptomatic and post-symptomatic transmission but only through scenarios based on assumed transmissibility from healthy (asymptomatic/presymptomatic or recovered cases) and the impact on e.g. the number of symptomatic infections |
| Du Z., Wang L., Cauchemez S., Xu X., Wang X., Cowling B.J., Meyers L.A. Risk for transportation of coronavirus disease from Wuhan to other cities in China. Emerging Infectious Diseases. 26 (5) (pp 1049-1052), 2020. Date of Publication: May 2020. | No primary data related to person-to-person transmission during asymptomatic, pre-symptomatic or post-symptomatic phases; this was a modelling study that did not incorporate asymptomatic/presymptomatic transmission |
| Du Z., Xu X., Wu Y., Wang L., Cowling B.J., Meyers L.A. Serial Interval of COVID-19 among Publicly Reported Confirmed Cases. Emerging infectious diseases. 26 (6) (pp 1341-1343), 2020. Date of Publication: 01 Jun 2020. | No primary data related to person-to-person transmission during asymptomatic, pre-symptomatic or post-symptomatic phases; negative serial intervals suggesting presymptomatic transmission were, however, discussed |
| Du Z; Xu X; Wu Y; Wang L; Cowling BJ; Meyers LA. Serial Interval of COVID-19 among Publicly Reported Confirmed Cases. Emerging Infectious Diseases. 26(6):1341-1343, 2020 06. | No primary data related to person-to-person transmission during asymptomatic, pre-symptomatic or post-symptomatic phases; negative serial intervals suggesting presymptomatic transmission were, however, discussed |
| Duanmu Y., Brown I.P., Gibb W.R., Singh J., Matheson L.W., Blomkalns A.L., Govindarajan P. Characteristics of Emergency Department Patients With COVID-19 at a Single Site in Northern California: Clinical Observations and Public Health Implications. Academic Emergency Medicine. (no pagination), 2020. Date of Publication: 2020. | No primary data related to person-to-person transmission during asymptomatic, pre-symptomatic or post-symptomatic phases |
| Duczmal LH; Almeida ACL; Duczmal DB; Alves CRL; Magalhaes FCO; Lima MS; Silva IR; Takahashi RHC. Vertical social distancing policy is ineffective to contain the COVID-19 pandemic. Cadernos de Saude Publica. 36(5):e00084420, 2020. | No primary data related to person-to-person transmission during asymptomatic, pre-symptomatic or post-symptomatic phases; this was a modelling study that acknowledged the possibility of asymptomatic/pre-symptomatic transmission but did not distinguish between these and symptomatic transmission in terms of results reported, e.g. numbers of people in different age groups who would become infected under different control measures |
| Ebrahim S.H., Memish Z.A. COVID-19: preparing for superspreader potential among Umrah pilgrims to Saudi Arabia. The Lancet. 395 (10227) (pp e48), 2020. Date of Publication: 14 - 20 March 2020. | No primary data related to person-to-person transmission during asymptomatic, pre-symptomatic or post-symptomatic phases |
| Eikenberry SE; Mancuso M; Iboi E; Phan T; Eikenberry K; Kuang Y; Kostelich E; Gumel AB. To mask or not to mask: Modeling the potential for face mask use by the general public to curtail the COVID-19 pandemic. Infectious Disease Modelling. 5:293-308, 2020. | No primary data related to person-to-person transmission during asymptomatic, pre-symptomatic or post-symptomatic phases; this was a modelling study that incorporated asymptomatic/presymptomatic transmission but only through scenarios based on assumed transmissibility and the impact on e.g. the total number of deaths that would result; relative infectivity of asymptomatic cases reported or assumed in other articles was, however, discussed |
| Epidemiology Working Group for NCIP Epidemic Response, Chinese Center for Disease Control and Prevention. [The epidemiological characteristics of an outbreak of 2019 novel coronavirus diseases (COVID-19) in China]. [Chinese] Chung-Hua Liu Hsing Ping Hsueh Tsa Chih Chinese Journal of Epidemiology. 41(2):145-151, 2020 Feb 10. | No primary data related to person-to-person transmission during asymptomatic, pre-symptomatic or post-symptomatic phases |
| Eubank S., Eckstrand I., Lewis B., Venkatramanan S., Marathe M., Barrett C.L. Commentary on Ferguson, et al., "Impact of Non-pharmaceutical Interventions (NPIs) to Reduce COVID-19 Mortality and Healthcare Demand". Bulletin of mathematical biology. 82 (4) (pp 52), 2020. Date of Publication: 08 Apr 2020. | No primary data related to person-to-person transmission during asymptomatic, pre-symptomatic or post-symptomatic phases; this is a commentary on Ferguson 2020^[76]^ |
| Fan J; Liu X; Pan W; Douglas MW; Bao S. Epidemiology of Coronavirus Disease in Gansu Province, China, 2020. Emerging Infectious Diseases. 26(6):1257-1265, 2020 06. | No primary data related to person-to-person transmission during asymptomatic, pre-symptomatic or post-symptomatic phases; several clusters were reported but the symptom status of the index patients at the time of contact with other patients was not reported |
| Fang Y., Nie Y., Penny M. Transmission dynamics of the COVID-19 outbreak and effectiveness of government interventions: A data-driven analysis. Journal of Medical Virology. 92 (6) (pp 645-659), 2020. Date of Publication: 01 Jun 2020. | No primary data related to person-to-person transmission during asymptomatic, pre-symptomatic or post-symptomatic phases; this was a modelling study that did not incorporate asymptomatic/presymptomatic transmission |
| Ferguson NM, Laydon D, Nedjati-Gilani G, Imai N, Ainslie K, Baguelin M, Bhatia S, Boonyasiri A, Cucunubá Z, Cuomo-Dannenburg G, Dighe A, Dorigatti I, Fu H, Gaythorpe K, Green W, Hamlet A, Hinsley W, Okell LC, van Elsland S, Thompson H, Verity R, Volz E, Wang H, Wang Y, Walker PGT, Walters C, Winskill P, Whittaker C, Donnelly CA, Riley S, Ghani AC Report 9: Impact of non-pharmaceutical interventions (NPIs) to reduce COVID-19 mortality and healthcare demand Imperial College COVID-19 reports, https://www.imperial.ac.uk/mrc-global-infectious-disease-analysis/covid-19/covid-19-reports/ | No primary data related to person-to-person transmission during asymptomatic, pre-symptomatic or post-symptomatic phases; this was a modelling study that incorporated asymptomatic/presymptomatic transmission but only through scenarios based on assumed transmissibility and the impact on e.g. the number of deaths |
| Ferreli F., Gaino F., Cecconi M., Costantini E., Spriano G., Mercante G. CORONA-steps for tracheotomy in COVID-19 patients: A staff-safe method for airway management. Oral Oncology. 105 (no pagination), 2020. Article Number: 104728. Date of Publication: June 2020. | No primary data related to person-to-person transmission during asymptomatic, pre-symptomatic or post-symptomatic phases |
| Frieden T.R., Lee C.T. Identifying and Interrupting Superspreading Events-Implications for Control of Severe Acute Respiratory Syndrome Coronavirus 2. Emerging infectious diseases. 26 (6) (pp 1059-1066), 2020. Date of Publication: 01 Jun 2020. | No primary data related to person-to-person transmission during asymptomatic, pre-symptomatic or post-symptomatic phases |
| Furukawa NW; Brooks JT; Sobel J. Evidence Supporting Transmission of Severe Acute Respiratory Syndrome Coronavirus 2 While Presymptomatic or Asymptomatic. Emerging Infectious Diseases. 26(7), 2020 May 04. | No primary data related to person-to-person transmission during asymptomatic, pre-symptomatic or post-symptomatic phases |
| Gan H; Zhang Y; Yuan M; Wu XY; Liu ZR; Liu M; Wu JB; Xu SJ; Gong L; Xu HL; Tao FB. [Epidemiological analysis on 1 052 cases of COVID-19 in epidemic clusters]. [Chinese] Chung-Hua Liu Hsing Ping Hsueh Tsa Chih Chinese Journal of Epidemiology. 41(5):E027, 2020 Mar 26. | No primary data related to person-to-person transmission during asymptomatic, pre-symptomatic or post-symptomatic phases |
| Gandhi M; Havlir D. The Time for Universal Masking of the Public for Coronavirus Disease 2019 Is Now. Open Forum Infectious Diseases. 7(4):ofaa131, 2020 Apr. | No primary data related to person-to-person transmission during asymptomatic, pre-symptomatic or post-symptomatic phases |
| Gandhi M; Yokoe DS; Havlir DV. Asymptomatic Transmission, the Achilles' Heel of Current Strategies to Control Covid-19. New England Journal of Medicine. 382(22):2158-2160, 2020 05 28. | No primary data related to person-to-person transmission during asymptomatic, pre-symptomatic or post-symptomatic phases |
| Ganyani T; Kremer C; Chen D; Torneri A; Faes C; Wallinga J; Hens N. Estimating the generation interval for coronavirus disease (COVID-19) based on symptom onset data, March 2020. Euro Surveillance: Bulletin Europeen sur les Maladies Transmissibles = European Communicable Disease Bulletin. 25(17), 2020 04. | No primary data related to person-to-person transmission during asymptomatic, pre-symptomatic or post-symptomatic phases; estimated proportion of presymptomatic transmissions was, however, discussed |
| Gao WJ; Li LM. [Advances on presymptomatic or asymptomatic carrier transmission of COVID-19]. [Chinese] Chung-Hua Liu Hsing Ping Hsueh Tsa Chih Chinese Journal of Epidemiology. 41(4):485-488, 2020 Apr 10. | No primary data related to person-to-person transmission during asymptomatic, pre-symptomatic or post-symptomatic phases |
| Ghinai I., Woods S., Ritger K.A., McPherson T.D., Black S.R., Sparrow L., Fricchione M.J., Kerins J.L., Pacilli M., Ruestow P.S., Arwady M.A., Beavers S.F., Payne D.C., Kirking H.L., Layden J.E. Community Transmission of SARS-CoV-2 at Two Family Gatherings - Chicago, Illinois, February-March 2020. MMWR. Morbidity and mortality weekly report. 69 (15) (pp 446-450), 2020. Date of Publication: 17 Apr 2020. | No primary data related to person-to-person transmission during asymptomatic, pre-symptomatic or post-symptomatic phases; the index patient had symptomatic contact with at least some of the other patients and exposure histories were not reported fully |
| Ghinai I; McPherson TD; Hunter JC; Kirking HL; Christiansen D; Joshi K; Rubin R; Morales-Estrada S; Black SR; Pacilli M; Fricchione MJ; Chugh RK; Walblay KA; Ahmed NS; Stoecker WC; Hasan NF; Burdsall DP; Reese HE; Wallace M; Wang C; Moeller D; Korpics J; Novosad SA; Benowitz I; Jacobs MW; Dasari VS; Patel MT; Kauerauf J; Charles EM; Ezike NO; Chu V; Midgley CM; Rolfes MA; Gerber SI; Lu X; Lindstrom S; Verani JR; Layden JE; Illinois COVID-19 Investigation Team. First known person-to-person transmission of severe acute respiratory syndrome coronavirus 2 (SARS-CoV-2) in the USA. Lancet. 395(10230):1137-1144, 2020 04 04. | No primary data related to person-to-person transmission during asymptomatic, pre-symptomatic or post-symptomatic phases; transmission occurred during symptomatic phase |
| Giordano G; Blanchini F; Bruno R; Colaneri P; Di Filippo A; Di Matteo A; Colaneri M. Modelling the COVID-19 epidemic and implementation of population-wide interventions in Italy. Nature Medicine. 2020 Apr 22. | No primary data related to person-to-person transmission during asymptomatic, pre-symptomatic or post-symptomatic phases; this was a modelling study that incorporated asymptomatic/presymptomatic transmission but only through scenarios based on assumed transmissibility and the impact on e.g. the proportion of the population that would become infected; serial intervals being short in relation to incubation periods and therefore suggesting presymptomatic transmission was, however, discussed |
| Gondauri D., Mikautadze E., Batiashvili M. Research on COVID-19 virus spreading statistics based on the examples of the cases from different counties. Electronic Journal of General Medicine. 17 (4) (no pagination), 2020. Article Number: em209. Date of Publication: 2020. | No primary data related to person-to-person transmission during asymptomatic, pre-symptomatic or post-symptomatic phases; this was a modelling study that did not incorporate asymptomatic/presymptomatic transmission |
| Gostic K; Gomez AC; Mummah RO; Kucharski AJ; Lloyd-Smith JO. Estimated effectiveness of symptom and risk screening to prevent the spread of COVID-19. eLife. 9, 2020 02 24. | No primary data related to person-to-person transmission during asymptomatic, pre-symptomatic or post-symptomatic phases; this was a modelling study that incorporated asymptomatic/presymptomatic states but focusing on detection of infected people through screening, rather than transmissibility of infection |
| Gou FX; Zhang XS; Yao JX; Yu DS; Wei KF; Zhang H; Yang XT; Yang JJ; Liu HX; Cheng Y; Jiang XJ; Zheng YH; Wu B; Liu XF; Li H. [Epidemiological characteristics of COVID-19 in Gansu province]. [Chinese] Chung-Hua Liu Hsing Ping Hsueh Tsa Chih Chinese Journal of Epidemiology. 41(0):E032, 2020 Apr 01. | No primary data related to person-to-person transmission during asymptomatic, pre-symptomatic or post-symptomatic phases |
| Gudbjartsson DF; Helgason A; Jonsson H; Magnusson OT; Melsted P; Norddahl GL; Saemundsdottir J; Sigurdsson A; Sulem P; Agustsdottir AB; Eiriksdottir B; Fridriksdottir R; Gardarsdottir EE; Georgsson G; Gretarsdottir OS; Gudmundsson KR; Gunnarsdottir TR; Gylfason A; Holm H; Jensson BO; Jonasdottir A; Jonsson F; Josefsdottir KS; Kristjansson T; Magnusdottir DN; le Roux L; Sigmundsdottir G; Sveinbjornsson G; Sveinsdottir KE; Sveinsdottir M; Thorarensen EA; Thorbjornsson B; Love A; Masson G; Jonsdottir I; Moller AD; Gudnason T; Kristinsson KG; Thorsteinsdottir U; Stefansson K. Spread of SARS-CoV-2 in the Icelandic Population. New England Journal of Medicine. 2020 Apr 14. | No primary data related to person-to-person transmission during asymptomatic, pre-symptomatic or post-symptomatic phases |
| Guo X; Wang J; Hu D; Wu L; Gu L; Wang Y; Zhao J; Zeng L; Zhang J; Wu Y. Survey of COVID-19 Disease Among Orthopaedic Surgeons in Wuhan, People's Republic of China. Journal of Bone & Joint Surgery - American Volume. 102(10):847-854, 2020 05 20. | No primary data related to person-to-person transmission during asymptomatic, pre-symptomatic or post-symptomatic phases |
| Han Y; Jiang M; Xia D; He L; Lv X; Liao X; Meng J. COVID-19 in a patient with long-term use of glucocorticoids: A study of a familial cluster. Clinical Immunology. 214:108413, 2020 05. | No primary data related to person-to-person transmission during asymptomatic, pre-symptomatic or post-symptomatic phases; the index patient had symptomatic contact with at least some of the other patients; subsequent exposure histories were not reported fully and the risk cannot be quantified (no denominator) |
| Han Y; Yang H. The transmission and diagnosis of 2019 novel coronavirus infection disease (COVID-19): A Chinese perspective. Journal of Medical Virology. 2020 Mar 06. | No primary data related to person-to-person transmission during asymptomatic, pre-symptomatic or post-symptomatic phases |
| Heinzerling A; Stuckey MJ; Scheuer T; Xu K; Perkins KM; Resseger H; Magill S; Verani JR; Jain S; Acosta M; Epson E. Transmission of COVID-19 to Health Care Personnel During Exposures to a Hospitalized Patient - Solano County, California, February 2020. MMWR - Morbidity & Mortality Weekly Report. 69(15):472-476, 2020 Apr 17. | No primary data related to person-to-person transmission during asymptomatic, pre-symptomatic or post-symptomatic phases; transmission to health care workers occurred during patient's symptomatic phase |
| Hellewell J; Abbott S; Gimma A; Bosse NI; Jarvis CI; Russell TW; Munday JD; Kucharski AJ; Edmunds WJ; Centre for the Mathematical Modelling of Infectious Diseases COVID-19 Working Group; Funk S; Eggo RM. Feasibility of controlling COVID-19 outbreaks by isolation of cases and contacts. The Lancet Global Health. 8(4):e488-e496, 2020 04. | No primary data related to person-to-person transmission during asymptomatic, pre-symptomatic or post-symptomatic phases; this was a modelling study that incorporated asymptomatic/presymptomatic transmission but only through scenarios based on the proportion of transmission that occurs before symptom onset and the impact on e.g. the probability of controlling an outbreak; correction also excluded |
| Helmy YA; Fawzy M; Elaswad A; Sobieh A; Kenney SP; Shehata AA. The COVID-19 Pandemic: A Comprehensive Review of Taxonomy, Genetics, Epidemiology, Diagnosis, Treatment, and Control. [Review] Journal of Clinical Medicine. 9(4), 2020 Apr 24. | No primary data related to person-to-person transmission during asymptomatic, pre-symptomatic or post-symptomatic phases |
| Hindson J. COVID-19: faecal-oral transmission?. Nature Reviews Gastroenterology and Hepatology. 17 (5) (pp 259), 2020. Date of Publication: 01 May 2020. | No primary data related to person-to-person transmission during asymptomatic, pre-symptomatic or post-symptomatic phases |
| Hsih WH; Cheng MY; Ho MW; Chou CH; Lin PC; Chi CY; Liao WC; Chen CY; Leong LY; Tien N; Lai HC; Lai YC; Lu MC. Featuring COVID-19 cases via screening symptomatic patients with epidemiologic link during flu season in a medical center of central Taiwan. Journal of Microbiology, Immunology & Infection. 2020 Mar 13. | Exposure history of COVID-19 patients incomplete; possibility of exposure during symptomatic phase |
| Hua J; Chen R; Zhao L; Wu X; Guo Q; He C; Li T; Ren X; Liu Z; Li Q; Wang F. Epidemiological features and medical care-seeking process of patients with COVID-19 in Wuhan, China. Erj Open Research. 6(2), 2020 Apr. | No primary data related to person-to-person transmission during asymptomatic, pre-symptomatic or post-symptomatic phases |
| Huff HV; Singh A. Asymptomatic transmission during the COVID-19 pandemic and implications for public health strategies. Clinical Infectious Diseases. 2020 May 28. | No primary data related to person-to-person transmission during asymptomatic, pre-symptomatic or post-symptomatic phases |
| Imai N, Cori A, Dorigatti I, Baguelin M, Donnelly CA, Riley S, Ferguson NM Report 3: Transmissibility of 2019-nCoV Imperial College COVID-19 reports, https://www.imperial.ac.uk/mrc-global-infectious-disease-analysis/covid-19/covid-19-reports/ | No primary data related to person-to-person transmission during asymptomatic, pre-symptomatic or post-symptomatic phases; this was a modelling study that did not incorporate asymptomatic/presymptomatic transmission |
| Islam MS; Rahman KM; Sun Y; Qureshi MO; Abdi I; Chughtai AA; Seale H. Current knowledge of COVID-19 and infection prevention and control strategies in healthcare settings: A global analysis. Infection Control & Hospital Epidemiology. 1-11, 2020 May 15. | No primary data related to person-to-person transmission during asymptomatic, pre-symptomatic or post-symptomatic phases |
| Iwata K; Miyakoshi C. A Simulation on Potential Secondary Spread of Novel Coronavirus in an Exported Country Using a Stochastic Epidemic SEIR Model. Journal of Clinical Medicine. 9(4), 2020 Mar 30. | No primary data related to person-to-person transmission during asymptomatic, pre-symptomatic or post-symptomatic phases; this was a modelling study that did not incorporate asymptomatic/presymptomatic transmission |
| James A; Eagle L; Phillips C; Hedges DS; Bodenhamer C; Brown R; Wheeler JG; Kirking H. High COVID-19 Attack Rate Among Attendees at Events at a Church - Arkansas, March 2020. MMWR - Morbidity & Mortality Weekly Report. 69(20):632-635, 2020 May 22. | No primary data related to person-to-person transmission during asymptomatic, pre-symptomatic or post-symptomatic phases; the transmission chain was initiated during the symptomatic phase of the primary cases; other exposure histories were not reported fully and the risk of asymptomatic/presymptomatic transmission cannot be quantified (denominator uncertain) |
| Jarvis CI; Van Zandvoort K; Gimma A; Prem K; CMMID COVID-19 working group; Klepac P; Rubin GJ; Edmunds WJ. Quantifying the impact of physical distance measures on the transmission of COVID-19 in the UK. BMC Medicine. 18(1):124, 2020 05 07. | No primary data related to person-to-person transmission during asymptomatic, pre-symptomatic or post-symptomatic phases; this study used a survey of personal contacts and behaviours to estimate the impact of social distancing measures on the reproduction number |
| Javid B., Weekes M.P., Matheson N.J. Covid-19: should the public wear face masks?. The BMJ. 369 (no pagination), 2020. Article Number: m1442. Date of Publication: 09 Apr 2020. | No primary data related to person-to-person transmission during asymptomatic, pre-symptomatic or post-symptomatic phases |
| Jia J; Hu X; Yang F; Song X; Dong L; Zhang J; Jiang F; Gao R. Epidemiological Characteristics on the Clustering Nature of COVID-19 in Qingdao City, 2020: A Descriptive Analysis. Disaster Medicine & Public Health Preparedness. 1-5, 2020 Mar 31. | No primary data related to person-to-person transmission during asymptomatic, pre-symptomatic or post-symptomatic phases |
| Jiang XL; Zhang XL; Zhao XN; Li CB; Lei J; Kou ZQ; Sun WK; Hang Y; Gao F; Ji SX; Lin CF; Pang B; Yao MX; Anderson BD; Wang GL; Yao L; Duan LJ; Kang DM; Ma MJ. Transmission potential of asymptomatic and paucisymptomatic SARS-CoV-2 infections: a three-family cluster study in China. Journal of Infectious Diseases. 2020 Apr 22. | No primary data related to person-to-person transmission during asymptomatic, pre-symptomatic or post-symptomatic phases |
| Jin YH; Huang Q; Wang YY; Zeng XT; Luo LS; Pan ZY; Yuan YF; Chen ZM; Cheng ZS; Huang X; Wang N; Li BH; Zi H; Zhao MJ; Ma LL; Deng T; Wang Y; Wang XH. Perceived infection transmission routes, infection control practices, psychosocial changes, and management of COVID-19 infected healthcare workers in a tertiary acute care hospital in Wuhan: a cross-sectional survey. Military Medical Research. 7(1):24, 2020 05 11. | No primary data related to person-to-person transmission during asymptomatic, pre-symptomatic or post-symptomatic phases |
| Jing QL; Li YG; Ma MM; Gu YZ; Li K; Ma Y; Wu D; Wu Y; Luo L; Zhang ZB. [Contagiousness and secondary attack rate of 2019 novel coronavirus based on cluster epidemics of COVID-19 in Guangzhou]. [Chinese] Chung-Hua Liu Hsing Ping Hsueh Tsa Chih Chinese Journal of Epidemiology. 41(0):E058, 2020 May 09. | No primary data related to person-to-person transmission during asymptomatic, pre-symptomatic or post-symptomatic phases |
| Joob B; Wiwanitkit V. Letter to the Editor: Coronavirus Disease 2019 (COVID-19), Infectivity, and the Incubation Period. Journal of Preventive Medicine & Public Health / Yebang Uihakhoe Chi. 53(2):70, 2020 Mar. | No primary data related to person-to-person transmission during asymptomatic, pre-symptomatic or post-symptomatic phases; comment on Bae 2020^[4]^ |
| Kakimoto K., Kamiya H., Yamagishi T., Matsui T., Suzuki M., Wakita T. Initial Investigation of Transmission of COVID-19 Among Crew Members During Quarantine of a Cruise Ship - Yokohama, Japan, February 2020. MMWR. Morbidity and mortality weekly report. 69 (11) (pp 312-313), 2020. Date of Publication: 20 Mar 2020. | No primary data related to person-to-person transmission during asymptomatic, pre-symptomatic or post-symptomatic phases; correction also excluded |
| Kam KQ; Yung CF; Cui L; Lin Tzer Pin R; Mak TM; Maiwald M; Li J; Chong CY; Nadua K; Tan NWH; Thoon KC. A Well Infant with Coronavirus Disease 2019 (COVID-19) with High Viral Load. Clinical Infectious Diseases. 2020 Feb 28. | No primary data related to person-to-person transmission during asymptomatic, pre-symptomatic or post-symptomatic phases |
| Karan A. To control the covid-19 outbreak, young, healthy patients should avoid the emergency department. The BMJ. 368 (no pagination), 2020. Article Number: m1040. Date of Publication: 17 Mar 2020. | No primary data related to person-to-person transmission during asymptomatic, pre-symptomatic or post-symptomatic phases |
| Keeley A.J., Evans C., Colton H., Ankcorn M., Cope A., State A., Bennett T., Giri P., de Silva T.I., Raza M. Roll-out of SARS-CoV-2 testing for healthcare workers at a large NHS Foundation Trust in the United Kingdom, March 2020. Eurosurveillance. 25 (14) (no pagination), 2020. Article Number: 2000433. Date of Publication: 09 Apr 2020. | No primary data related to person-to-person transmission during asymptomatic, pre-symptomatic or post-symptomatic phases |
| Kim GU; Kim MJ; Ra SH; Lee J; Bae S; Jung J; Kim SH. Clinical characteristics of asymptomatic and symptomatic patients with mild COVID-19. Clinical Microbiology & Infection. 2020 May 01. | No primary data related to person-to-person transmission during asymptomatic, pre-symptomatic or post-symptomatic phases |
| Kimball A; Hatfield KM; Arons M; James A; Taylor J; Spicer K; Bardossy AC; Oakley LP; Tanwar S; Chisty Z; Bell JM; Methner M; Harney J; Jacobs JR; Carlson CM; McLaughlin HP; Stone N; Clark S; Brostrom-Smith C; Page LC; Kay M; Lewis J; Russell D; Hiatt B; Gant J; Duchin JS; Clark TA; Honein MA; Reddy SC; Jernigan JA; Public Health - Seattle & King County; CDC COVID-19 Investigation Team. Asymptomatic and Presymptomatic SARS-CoV-2 Infections in Residents of a Long-Term Care Skilled Nursing Facility - King County, Washington, March 2020. MMWR - Morbidity & Mortality Weekly Report. 69(13):377-381, 2020 Apr 03. | No primary data related to person-to-person transmission during asymptomatic, pre-symptomatic or post-symptomatic phases; same study/setting as Arons 2020^[77]^ |
| Kirby T. UK senior police officer with COVID-19. The Lancet Respiratory Medicine. 8 (5) (pp 451), 2020. Date of Publication: May 2020. | No primary data related to person-to-person transmission during asymptomatic, pre-symptomatic or post-symptomatic phases |
| Kissler SM, Tedijanto C, Goldstein E, Grad YH, Lipsitch M Projecting the transmission dynamics of SARS-CoV-2 through the postpandemic period Science, Vol 368, Issue 6493, 22 May 2020 | No primary data related to person-to-person transmission during asymptomatic, pre-symptomatic or post-symptomatic phases |
| Kluytmans-van den Bergh MFQ; Buiting AGM; Pas SD; Bentvelsen RG; van den Bijllaardt W; van Oudheusden AJG; van Rijen MML; Verweij JJ; Koopmans MPG; Kluytmans JAJW. Prevalence and Clinical Presentation of Health Care Workers With Symptoms of Coronavirus Disease 2019 in 2 Dutch Hospitals During an Early Phase of the Pandemic. JAMA Network Open. 3(5):e209673, 2020 05 01. | No primary data related to person-to-person transmission during asymptomatic, pre-symptomatic or post-symptomatic phases |
| Kolifarhood G; Aghaali M; Mozafar Saadati H; Taherpour N; Rahimi S; Izadi N; Hashemi Nazari SS. Epidemiological and Clinical Aspects of COVID-19; a Narrative Review. [Review] Archives of Academic Emergency Medicine. 8(1):e41, 2020. | No primary data related to person-to-person transmission during asymptomatic, pre-symptomatic or post-symptomatic phases |
| Koo JR; Cook AR; Park M; Sun Y; Sun H; Lim JT; Tam C; Dickens BL. Interventions to mitigate early spread of SARS-CoV-2 in Singapore: a modelling study. The Lancet Infectious Diseases. 2020 Mar 23. | No primary data related to person-to-person transmission during asymptomatic, pre-symptomatic or post-symptomatic phases; this was a modelling study that incorporated asymptomatic/presymptomatic transmission but only through scenarios based on assumed transmissibility and the impact on e.g. the proportion of the population that would become infected; correction also excluded |
| Kucharski A.J., Russell T.W., Diamond C., Liu Y., Edmunds J., Funk S., Eggo R.M., Sun F., Jit M., Munday J.D., Davies N., Gimma A., van Zandvoort K., Gibbs H., Hellewell J., Jarvis C.I., Clifford S., Quilty B.J., Bosse N.I., Abbott S., Klepac P., Flasche S. Early dynamics of transmission and control of COVID-19: a mathematical modelling study. The Lancet Infectious Diseases. 20 (5) (pp 553-558), 2020. Date of Publication: May 2020. | No primary data related to person-to-person transmission during asymptomatic, pre-symptomatic or post-symptomatic phases; this was a modelling study that incorporated asymptomatic/presymptomatic transmission but only through scenarios based on assumed transmissibility and the impact on e.g. the reproduction number |
| Kwok K.O., Yu Wong V.W., Wei W.I., Shan Wong S.Y., Tang J.W.-T. Epidemiological characteristics of the first 53 laboratory-confirmed cases of COVID-19 epidemic in Hong Kong, 13 February 2020. Eurosurveillance. 25 (16) (no pagination), 2020. Article Number: 2000155. Date of Publication: 2020. | No primary data related to person-to-person transmission during asymptomatic, pre-symptomatic or post-symptomatic phases; article refers to possibility of pre-symptomatic transmission since the estimated serial interval is shorter than the estimated incubation period reported in other articles |
| Lai A., Bergna A., Acciarri C., Galli M., Zehender G. Early phylogenetic estimate of the effective reproduction number of SARS-CoV-2. Journal of Medical Virology. 92 (6) (pp 675-679), 2020. Date of Publication: 01 Jun 2020. | No primary data related to person-to-person transmission during asymptomatic, pre-symptomatic or post-symptomatic phases; this was a modelling study that did not incorporate asymptomatic/presymptomatic transmission |
| Lai CC; Liu YH; Wang CY; Wang YH; Hsueh SC; Yen MY; Ko WC; Hsueh PR. Asymptomatic carrier state, acute respiratory disease, and pneumonia due to severe acute respiratory syndrome coronavirus 2 (SARS-CoV-2): Facts and myths. [Review] Journal of Microbiology, Immunology & Infection. 2020 Mar 04. | No primary data related to person-to-person transmission during asymptomatic, pre-symptomatic or post-symptomatic phases |
| Lai X; Wang M; Qin C; Tan L; Ran L; Chen D; Zhang H; Shang K; Xia C; Wang S; Xu S; Wang W. Coronavirus Disease 2019 (COVID-2019) Infection Among Health Care Workers and Implications for Prevention Measures in a Tertiary Hospital in Wuhan, China. JAMA Network Open. 3(5):e209666, 2020 05 01. | No primary data related to person-to-person transmission during asymptomatic, pre-symptomatic or post-symptomatic phases |
| Lan L., Xu D., Ye G., Xia C., Wang S., Li Y., Xu H. Positive RT-PCR Test Results in Patients Recovered from COVID-19. JAMA - Journal of the American Medical Association. 323 (15) (pp 1502-1503), 2020. | No primary data related to person-to-person transmission during asymptomatic, pre-symptomatic or post-symptomatic phases |
| Le TQM; Takemura T; Moi ML; Nabeshima T; Nguyen LKH; Hoang VMP; Ung THT; Le TT; Nguyen VS; Pham HQA; Duong TN; Nguyen HT; Ngu DN; Nguyen CK; Morita K; Hasebe F; Dang DA. Severe Acute Respiratory Syndrome Coronavirus 2 Shedding by Travelers, Vietnam, 2020. Emerging Infectious Diseases. 26(7), 2020 Apr 02. | No primary data related to person-to-person transmission during asymptomatic, pre-symptomatic or post-symptomatic phases; possibility of symptomatic transmission in at least one case; exposure histories were not reported fully and the risk of asymptomatic/presymptomatic transmission cannot be quantified |
| Lee H; Heo JW; Kim SW; Lee J; Choi JH. A Lesson from Temporary Closing of a Single University-affiliated Hospital owing to In-Hospital Transmission of Coronavirus Disease 2019. Journal of Korean Medical Science. 35(13):e145, 2020 Apr 06. | No primary data related to person-to-person transmission during asymptomatic, pre-symptomatic or post-symptomatic phases |
| Lee JK; Jeong HW. Wearing face masks regardless of symptoms is crucial for preventing the spread of COVID-19 in hospitals. Infection Control & Hospital Epidemiology. 1-2, 2020 May 06. | No primary data related to person-to-person transmission during asymptomatic, pre-symptomatic or post-symptomatic phases |
| Lee V.J., Chiew C.J., Khong W.X. Interrupting transmission of COVID-19: lessons from containment efforts in Singapore. Journal of travel medicine. 27 (3) (no pagination), 2020. Date of Publication: 18 May 2020. | No primary data related to person-to-person transmission during asymptomatic, pre-symptomatic or post-symptomatic phases |
| Leung C.C., Lam T.H., Cheng K.K. Mass masking in the COVID-19 epidemic: people need guidance. The Lancet. 395 (10228) (pp 945), 2020. Date of Publication: 21 - 27 March 2020. | No primary data related to person-to-person transmission during asymptomatic, pre-symptomatic or post-symptomatic phases |
| Leung K., Wu J.T., Liu D., Leung G.M. First-wave COVID-19 transmissibility and severity in China outside Hubei after control measures, and second-wave scenario planning: a modelling impact assessment. The Lancet. 395 (10233) (pp 1382-1393), 2020. Date of Publication: 25 April - 1 May 2020. | No primary data related to person-to-person transmission during asymptomatic, pre-symptomatic or post-symptomatic phases; this was a modelling study that did not incorporate asymptomatic/presymptomatic transmission; negative serial intervals suggesting presymptomatic transmission were, however, discussed |
| Li CX; Wu B; Luo F; Zhang N. [Clinical Study and CT Findings of a Familial Cluster of Pneumonia with Coronavirus Disease 2019 (COVID-19)]. [Chinese] Sichuan da Xue Xue Bao. Yi Xue Ban/Journal of Sichuan University. Medical Science Edition. 51(2):155-158, 2020 Mar. | Unable to retrieve full text |
| Li J, Wang Y, Gilmour S, Wang M, Yoneoka D, Wang Y, You X, Gu J, Hao C, Peng L, Du Z, Xu DR, Hao Y Estimation of the epidemic properties of the 2019 novel coronavirus: A mathematical modeling study medRxiv, https://www.medrxiv.org/content/10.1101/2020.02.18.20024315v1 | No primary data related to person-to-person transmission during asymptomatic, pre-symptomatic or post-symptomatic phases; this was a modelling study that did not incorporate asymptomatic/presymptomatic transmission |
| Li Q., Guan X., Wu P., Wang X., Zhou L., Tong Y., Ren R., Leung K.S.M., Lau E.H.Y., Wong J.Y., Xing X., Xiang N., Wu Y., Li C., Chen Q., Li D., Liu T., Zhao J., Liu M., Tu W., Chen C., Jin L., Yang R., Wang Q., Zhou S., Wang R., Liu H., Luo Y., Liu Y., Shao G., Li H., Tao Z., Yang Y., Deng Z., Liu B., Ma Z., Zhang Y., Shi G., Lam T.T.Y., Wu J.T., Gao G.F., Cowling B.J., Yang B., Leung G.M., Feng Z. Early transmission dynamics in Wuhan, China, of novel coronavirus-infected pneumonia. New England Journal of Medicine. 382 (13) (pp 1199-1207), 2020. Date of Publication: 26 Mar 2020. | No primary data related to person-to-person transmission during asymptomatic, pre-symptomatic or post-symptomatic phases; this study includes modelling that did not incorporate asymptomatic/presymptomatic transmission |
| Li S; Song K; Yang B; Gao Y; Gao X. Preliminary Assessment of the COVID-19 Outbreak Using 3-Staged Model e-ISHR. Journal of Shanghai Jiaotong University Science. 25(2):157-164, 2020. | No primary data related to person-to-person transmission during asymptomatic, pre-symptomatic or post-symptomatic phases; this was a modelling study that did not incorporate asymptomatic/presymptomatic transmission |
| Li W; Zhang B; Lu J; Liu S; Chang Z; Cao P; Liu X; Zhang P; Ling Y; Tao K; Chen J. The characteristics of household transmission of COVID-19. Clinical Infectious Diseases. 2020 Apr 17. | No primary data related to person-to-person transmission during asymptomatic, pre-symptomatic or post-symptomatic phases |
| Li X; Wang W; Zhao X; Zai J; Zhao Q; Li Y; Chaillon A. Transmission dynamics and evolutionary history of 2019-nCoV. Journal of Medical Virology. 92(5):501-511, 2020 05. | No primary data related to person-to-person transmission during asymptomatic, pre-symptomatic or post-symptomatic phases; this was a study that analysed genome sequences to reconstruct transmission networks |
| Li X; Wang X; Liu J; Huang G; Shi X. Epidemiological characteristics of confirmed COVID-19 in Guizhou province, China. Disaster Medicine & Public Health Preparedness. 1-7, 2020 May 05. | No primary data related to person-to-person transmission during asymptomatic, pre-symptomatic or post-symptomatic phases |
| Lillie P.J., Samson A., Li A., Adams K., Capstick R., Barlow G.D., Easom N., Hamilton E., Moss P.J., Evans A., Ivan M., PHE Incident Team, Taha Y., Duncan C.J.A., Schmid M.L., the Airborne HCID Network Novel coronavirus disease (Covid-19): The first two patients in the UK with person to person transmission. Journal of Infection. 80 (5) (pp 578-606), 2020. Date of Publication: May 2020 | Transmission between patients assumed to occur during first patient's symptomatic phase; exposure history and outcomes for other close contacts incomplete |
| Lim J; Jeon S; Shin HY; Kim MJ; Seong YM; Lee WJ; Choe KW; Kang YM; Lee B; Park SJ. Case of the Index Patient Who Caused Tertiary Transmission of COVID-19 Infection in Korea: the Application of Lopinavir/Ritonavir for the Treatment of COVID-19 Infected Pneumonia Monitored by Quantitative RT-PCR. Journal of Korean Medical Science. 35(6):e79, 2020 Feb 17. | No primary data related to person-to-person transmission during asymptomatic, pre-symptomatic or post-symptomatic phases; the index patient had symptomatic contact with at least some of the other patients and subsequent exposure histories were not reported fully |
| Lin M., Beliavsky A., Katz K., Powis J.E., Ng W., Williams V., Science M., Groves H., Muller M.P., Vaisman A., Hota S., Johnstone J., Leis J.A. What can early Canadian experience screening for COVID-19 teach us about how to prepare for a pandemic?. CMAJ. 192 (12) (pp E314-E318), 2020. Date of Publication: 23 Mar 2020. | No primary data related to person-to-person transmission during asymptomatic, pre-symptomatic or post-symptomatic phases |
| Lipsitch M., Swerdlow D.L., Finelli L. Defining the epidemiology of Covid-19 - Studies needed. New England Journal of Medicine. 382 (13) (pp 1194-1196), 2020. Date of Publication: 26 Mar 2020. | No primary data related to person-to-person transmission during asymptomatic, pre-symptomatic or post-symptomatic phases |
| Liu F; Li X; Zhu G. Using the contact network model and Metropolis-Hastings sampling to reconstruct the COVID-19 spread on the "Diamond Princess". Science Bulletin. 2020 May 05. | No primary data related to person-to-person transmission during asymptomatic, pre-symptomatic or post-symptomatic phases; this was a modelling study that did not incorporate asymptomatic/presymptomatic transmission |
| Liu J., Liao X., Qian S., Yuan J., Wang F., Liu Y., Wang Z., Wang F.-S., Liu L., Zhang Z. Community Transmission of Severe Acute Respiratory Syndrome Coronavirus 2, Shenzhen, China, 2020. Emerging infectious diseases. 26 (6) (pp 1320-1323), 2020. Date of Publication: 01 Jun 2020. | No primary data related to person-to-person transmission during asymptomatic, pre-symptomatic or post-symptomatic phases |
| Liu JY; Chen TJ; Hwang SJ. Analysis of Imported Cases of COVID-19 in Taiwan: A Nationwide Study. International Journal of Environmental Research & Public Health [Electronic Resource]. 17(9), 2020 05 09. | No primary data related to person-to-person transmission during asymptomatic, pre-symptomatic or post-symptomatic phases |
| Liu L., Hong X., Su X., Chen H., Zhang D., Tang S., Chen L., Zhu B., Li X., Shi Y. Optimizing screening strategies for coronavirus disease 2019: A study from Middle China. Journal of Infection and Public Health. (no pagination), 2020. Date of Publication: 2020. | No primary data related to person-to-person transmission during asymptomatic, pre-symptomatic or post-symptomatic phases |
| Liu T., Hu J., Xiao J., He G., Kang M., Rong Z., Lin L., Zhong H., Huang Q., Deng A., Zeng W., Tan X., Zeng S., Zhu Z., Li J., Gong D., Wan D., Chen S., Guo L., Li Y., Sun L., Liang W., Song T., He J., Ma W. Time-varying transmission dynamics of Novel Coronavirus Pneumonia in China bioRxiv, https://doi.org/10.1101/2020.01.25.919787 | No primary data related to person-to-person transmission during asymptomatic, pre-symptomatic or post-symptomatic phases; this was a modelling study that did not incorporate asymptomatic/presymptomatic transmission |
| Liu T., Hu J., Kang M., Lin L., Zhong H., Xiao J., He G., Song T., Huang Q., Rong Z., Deng A., Zeng W., Tan X., Zeng S., Zhu Z., Li J., Wan D., Lu J., Deng H., He J., Ma W. Transmission dynamics of 2019 novel coronavirus (2019-nCoV) bioRxiv, https://doi.org/10.1101/2020.01.25.919787 | Earlier version of Liu 2020a^[78]^ |
| Liu Y., Eggo R.M., Kucharski A.J. Secondary attack rate and superspreading events for SARS-CoV-2. The Lancet. 395 (10227) (pp e47), 2020. Date of Publication: 14 - 20 March 2020. | No primary data related to person-to-person transmission during asymptomatic, pre-symptomatic or post-symptomatic phases |
| Liu Z; Huang S; Lu W; Su Z; Yin X; Liang H; Zhang H. Modeling the trend of coronavirus disease 2019 and restoration of operational capability of metropolitan medical service in China: a machine learning and mathematical model-based analysis. Global Health Research and Policy. 5:20, 2020. | No primary data related to person-to-person transmission during asymptomatic, pre-symptomatic or post-symptomatic phases; this was a modelling study that did not incorporate asymptomatic/presymptomatic transmission |
| Long YS, Zhai Z-M, Han L-L, Kang J, Li Y-L, Lin Z-H, Zeng L, Wu D-Y, Hao C-Q, Tang M, Liu Z, Lai Y-C Quantitative assessment of the role of undocumented infection in the 2019 novel coronavirus (COVID-19) pandemic arxiv, https://arxiv.org/abs/2003.12028 | No primary data related to person-to-person transmission during asymptomatic, pre-symptomatic or post-symptomatic phases; this was a modelling study that incorporated asymptomatic/presymptomatic transmission but only through scenarios based on assumed initial numbers of cases and the impact on e.g. the overall number of infections that would result |
| Lu D., Wang H., Yu R., Yang H., Zhao Y. Integrated infection control strategy to minimize nosocomial infection of coronavirus disease 2019 among ENT healthcare workers. Journal of Hospital Infection. 104 (4) (pp 454-455), 2020. Date of Publication: April 2020. | No primary data related to person-to-person transmission during asymptomatic, pre-symptomatic or post-symptomatic phases |
| Luan RS; Wang X; Sun X; Chen XS; Zhou T; Liu QH; Lu X; Wu XP; Gu DQ; Tang MS; Cui HJ; Shan XF; Ouyang J; Zhang B; Zhang W; Sichuan University Covid- ERG. [Epidemiology, Treatment, and Epidemic Prevention and Control of the Coronavirus Disease 2019: a Review]. [Review] [Chinese] Sichuan da Xue Xue Bao. Yi Xue Ban/Journal of Sichuan University. Medical Science Edition. 51(2):131-138, 2020 Mar. | Unable to retrieve full text |
| Luo C., Yao L., Zhang L., Yao M., Chen X., Wang Q., Shen H. Possible Transmission of Severe Acute Respiratory Syndrome Coronavirus 2 (SARS-CoV-2) in a Public Bath Center in Huai'an, Jiangsu Province, China. JAMA Network Open. (no pagination), 2020. Date of Publication: 2020. | No primary data related to person-to-person transmission during asymptomatic, pre-symptomatic or post-symptomatic phases; the index patient had symptomatic contact with the other patients |
| Ma Y; Xu QN; Wang FL; Ma XM; Wang XY; Zhang XG; Zhang ZF. Characteristics of asymptomatic patients with SARS-CoV-2 infection in Jinan, China. Microbes & Infection. 2020 May 07. | No primary data related to person-to-person transmission during asymptomatic, pre-symptomatic or post-symptomatic phases |
| MacIntyre CR. On a knife's edge of a COVID-19 pandemic: is containment still possible?. Public Health Research & Practice. 30(1), 2020 03 10. | No primary data related to person-to-person transmission during asymptomatic, pre-symptomatic or post-symptomatic phases |
| Maier BF; Brockmann D. Effective containment explains subexponential growth in recent confirmed COVID-19 cases in China. Science. 368(6492):742-746, 2020 05 15. | No primary data related to person-to-person transmission during asymptomatic, pre-symptomatic or post-symptomatic phases; this was a modelling study that did not incorporate asymptomatic/presymptomatic transmission |
| Majumder MS, Mandl, KD Early transmissibility assessment of a novel coronavirus in Wuhan, China SSRN’s Coronavirus and Infectious Disease Research page, https://www.ssrn.com/index.cfm/en/coronavirus/ | No primary data related to person-to-person transmission during asymptomatic, pre-symptomatic or post-symptomatic phases; this was a modelling study that did not incorporate asymptomatic/presymptomatic transmission |
| Manchein C; Brugnago EL; da Silva RM; Mendes CFO; Beims MW. Strong correlations between power-law growth of COVID-19 in four continents and the inefficiency of soft quarantine strategies. Chaos. 30(4):041102, 2020 Apr. | No primary data related to person-to-person transmission during asymptomatic, pre-symptomatic or post-symptomatic phases; this study included modelling that incorporated asymptomatic/presymptomatic transmission but only through scenarios based on assumed transmissibility and the impact on e.g. prevalence estimates |
| Mandal S; Bhatnagar T; Arinaminpathy N; Agarwal A; Chowdhury A; Murhekar M; Gangakhedkar RR; Sarkar S. Prudent public health intervention strategies to control the coronavirus disease 2019 transmission in India: A mathematical model-based approach. Indian Journal of Medical Research. 151(2 & 3):190-199, 2020 Feb & Mar. | No primary data related to person-to-person transmission during asymptomatic, pre-symptomatic or post-symptomatic phases; this was a modelling study that incorporated asymptomatic/presymptomatic states but only through scenarios based on assumed proportions of asymptomatic/presymptomatic infections and the impact on e.g. peak prevalence |
| Mao LJ; Xu J; Xu ZH; Xia XP; Li B; He JG; Zhao P; Pan JW; Zhang D; Su Y; Wang YH; Yuan ZF. A child with household transmitted COVID-19. BMC Infectious Diseases. 20(1):329, 2020 May 07. | No primary data related to person-to-person transmission during asymptomatic, pre-symptomatic or post-symptomatic phases; there was a possibility that asymptomatic/presymptomatic transmission had occurred but exposure histories were not reported fully and the risk cannot be quantified (no denominator) |
| Mayor S. Covid-19: Researchers launch app to track spread of symptoms in the UK. BMJ. 368:m1263, 2020 03 27. | No primary data related to person-to-person transmission during asymptomatic, pre-symptomatic or post-symptomatic phases |
| McMichael TM; Currie DW; Clark S; Pogosjans S; Kay M; Schwartz NG; Lewis J; Baer A; Kawakami V; Lukoff MD; Ferro J; Brostrom-Smith C; Rea TD; Sayre MR; Riedo FX; Russell D; Hiatt B; Montgomery P; Rao AK; Chow EJ; Tobolowsky F; Hughes MJ; Bardossy AC; Oakley LP; Jacobs JR; Stone ND; Reddy SC; Jernigan JA; Honein MA; Clark TA; Duchin JS; Public Health-Seattle and King County, EvergreenHealth, and CDC COVID-19 Investigation Team. Epidemiology of Covid-19 in a Long-Term Care Facility in King County, Washington. New England Journal of Medicine. 382(21):2005-2011, 2020 05 21. | No primary data related to person-to-person transmission during asymptomatic, pre-symptomatic or post-symptomatic phases |
| Meng H; Xiong R; He R; Lin W; Hao B; Zhang L; Lu Z; Shen X; Fan T; Jiang W; Yang W; Li T; Chen J; Geng Q. CT imaging and clinical course of asymptomatic cases with COVID-19 pneumonia at admission in Wuhan, China. Journal of Infection. 2020 Apr 12. | No primary data related to person-to-person transmission during asymptomatic, pre-symptomatic or post-symptomatic phases |
| Merza MA; Haleem Al Mezori AA; Mohammed HM; Abdulah DM. COVID-19 outbreak in Iraqi Kurdistan: The first report characterizing epidemiological, clinical, laboratory, and radiological findings of the disease. Diabetes & Metabolic Syndrome. 14(4):547-554, 2020 May 05. | Possibility that asymptomatic/presymptomatic transmission had occurred but exposure histories were not reported fully and the risk cannot be quantified (no denominator) |
| Miller R; Englund K. Transmission and risk factors of OF COVID-19. Cleveland Clinic Journal of Medicine. 2020 May 14. | No primary data related to person-to-person transmission during asymptomatic, pre-symptomatic or post-symptomatic phases |
| Mirmohammadkhani M., Paknazar F., Rashidy-Pour A. Evaluation of the epidemiological pattern of covid-19 applying basic reproduction number: An educational review article. Koomesh. 22 (3) (pp 373-379), 2020. Date of Publication: 2020. | Unable to translate full text |
| Mizumoto K; Chowell G. Transmission potential of the novel coronavirus (COVID-19) onboard the diamond Princess Cruises Ship, 2020. Infectious Disease Modelling. 5:264-270, 2020. | No primary data related to person-to-person transmission during asymptomatic, pre-symptomatic or post-symptomatic phases; this was a modelling study that did not incorporate asymptomatic/presymptomatic transmission |
| Mizumoto K; Kagaya K; Zarebski A; Chowell G. Estimating the asymptomatic proportion of coronavirus disease 2019 (COVID-19) cases on board the Diamond Princess cruise ship, Yokohama, Japan, 2020. Euro Surveillance: Bulletin Europeen sur les Maladies Transmissibles = European Communicable Disease Bulletin. 25(10), 2020 03. | No primary data related to person-to-person transmission during asymptomatic, pre-symptomatic or post-symptomatic phases |
| Moore SE, Okyere E Controlling the transmission dynamics of COVID-19 arxiv, https://arxiv.org/abs/2004.00443 | No primary data related to person-to-person transmission during asymptomatic, pre-symptomatic or post-symptomatic phases; this was a modelling study that did not incorporate asymptomatic/presymptomatic transmission |
| Mosites E; Parker EM; Clarke KEN; Gaeta JM; Baggett TP; Imbert E; Sankaran M; Scarborough A; Huster K; Hanson M; Gonzales E; Rauch J; Page L; McMichael TM; Keating R; Marx GE; Andrews T; Schmit K; Morris SB; Dowling NF; Peacock G; COVID-19 Homelessness Team. Assessment of SARS-CoV-2 Infection Prevalence in Homeless Shelters - Four U.S. Cities, March 27-April 15, 2020. MMWR - Morbidity & Mortality Weekly Report. 69(17):521-522, 2020 May 01. | No primary data related to person-to-person transmission during asymptomatic, pre-symptomatic or post-symptomatic phases; possible overlap with Tobolowsky 2020^[79]^ in terms of study settings/participants |
| Munayco C.V., Tariq A., Rothenberg R., Soto-Cabezas G.G., Reyes M.F., Valle A., Rojas-Mezarina L., Cabezas C., Loayza M., Chowell G., Garro D.C., Vasquez K.M., Castro E.S., Ordinola I.S., Mimbela J.M., Cornejo K.M., Quijano F.C., La Torre Rosillo L., Ibarguen L.O., Dominguez M.V., Gonzalez Seminario R.V., Silva M.C., Dreyfus M.S., Pineda M.L., Durand M., Janampa N., Chuquihuaccha J., Lizarbe S.M., Cusi D.E., Pilco I.M., Jaramillo A., Vargas K., Cabanillas O., Arrasco J., Vargas M., Ramos W. Early transmission dynamics of COVID-19 in a southern hemisphere setting: Lima-Peru: February 29th-March 30th, 2020. Infectious Disease Modelling. 5 (pp 338-345), 2020. Date of Publication: 2020. | No primary data related to person-to-person transmission during asymptomatic, pre-symptomatic or post-symptomatic phases |
| Ng O.-T., Marimuthu K., Chia P.-Y., Koh V., Chiew C.J., de Wang L., Young B.E., Chan M., Vasoo S., Ling L.-M., Lye D.C., Kam K.-Q., Thoon K.-C., Kurupatham L., Said Z., Goh E., Low C., Lim S.-K., Raj P., Oh O., Koh V.T.J., Poh C., Mak T.-M., Cui L., Cook A.R., Lin R.T.P., Leo Y.-S., Lee V.J.M. SARS-CoV-2 infection among travelers returning from Wuhan, China. New England Journal of Medicine. 382 (15) (pp 1476-1478), 2020. Date of Publication: 09 Apr 2020. | No primary data related to person-to-person transmission during asymptomatic, pre-symptomatic or post-symptomatic phases |
| Nicastri E; D'Abramo A; Faggioni G; De Santis R; Mariano A; Lepore L; Molinari F; Petralito G; Fillo S; Munzi D; Corpolongo A; Bordi L; Carletti F; Castiletti C; Colavita F; Lalle E; Bevilacqua N; Giancola ML; Scorzolini L; Lanini S; Palazzolo C; De Domenico A; Spinelli MA; Scognamiglio P; Piredda P; Iacomino R; Mone A; Puro V; Petrosillo N; Battistini A; Vairo F; Lista F; Ippolito G; On Behalf Of Inmi And The Italian Army Covid-Study Groups. Coronavirus disease (COVID-19) in a paucisymptomatic patient: epidemiological and clinical challenge in settings with limited community transmission, Italy, February 2020. Euro Surveillance: Bulletin Europeen sur les Maladies Transmissibles = European Communicable Disease Bulletin. 25(11), 2020 03. | No primary data related to person-to-person transmission during asymptomatic, pre-symptomatic or post-symptomatic phases; incomplete exposure history for co-evacuees |
| Niehus R; De Salazar PM; Taylor AR; Lipsitch M. Using observational data to quantify bias of traveller-derived COVID-19 prevalence estimates in Wuhan, China. The Lancet Infectious Diseases. 2020 Apr 01. | No primary data related to person-to-person transmission during asymptomatic, pre-symptomatic or post-symptomatic phases |
| Nishiura H; Linton NM; Akhmetzhanov AR. Serial interval of novel coronavirus (COVID-19) infections. International Journal of Infectious Diseases. 93:284-286, 2020 Apr. | No primary data related to person-to-person transmission during asymptomatic, pre-symptomatic or post-symptomatic phases; article refers to possibility of pre-symptomatic transmission since the estimated serial interval is shorter than the estimated incubation period reported in other articles |
| Normile D. Airport screening is largely futile, research shows. Science. 367 (6483) (pp 1177-1178), 2020. Date of Publication: 13 Mar 2020. | No primary data related to person-to-person transmission during asymptomatic, pre-symptomatic or post-symptomatic phases |
| Normile D., Cohen J., Enserink M., Huihui B. As normalcy returns, can China keep COVID-19 at bay? Infected travelers pose a continuing threat, but local coronavirus transmission still occurs as well. Science. 368 (6486) (pp 18-19), 2020. Date of Publication: 03 Apr 2020. | No primary data related to person-to-person transmission during asymptomatic, pre-symptomatic or post-symptomatic phases |
| Nussbaumer-Streit B; Mayr V; Dobrescu AI; Chapman A; Persad E; Klerings I; Wagner G; Siebert U; Christof C; Zachariah C; Gartlehner G. Quarantine alone or in combination with other public health measures to control COVID-19: a rapid review. Cochrane Database of Systematic Reviews. 4:CD013574, 2020 04 08. | No primary data related to person-to-person transmission during asymptomatic, pre-symptomatic or post-symptomatic phases |
| Okada P., Buathong R., Phuygun S., Thanadachakul T., Parnmen S., Wongboot W., Waicharoen S., Wacharapluesadee S., Uttayamakul S., Vachiraphan A., Chittaganpitch M., Mekha N., Janejai N., Iamsirithaworn S., Lee R.T.C., Maurer-Stroh S. Early transmission patterns of coronavirus disease 2019 (COVID-19) in travellers from Wuhan to Thailand, January 2020. Eurosurveillance. 25 (8) (no pagination), 2020. Article Number: 2000097. Date of Publication: 27 Feb 2020. | No primary data related to person-to-person transmission during asymptomatic, pre-symptomatic or post-symptomatic phases; the index patients had symptomatic contact with at least some of the other patients and exposure histories were not reported fully |
| Ornelas-Aguirre J.M. The new coronavirus that came from the East: analysis of the initial epidemic in Mexico. Gaceta medica de Mexico. 156 (4) (no pagination), 2020. Date of Publication: 14 May 2020. | No primary data related to person-to-person transmission during asymptomatic, pre-symptomatic or post-symptomatic phases |
| Pan A; Liu L; Wang C; Guo H; Hao X; Wang Q; Huang J; He N; Yu H; Lin X; Wei S; Wu T. Association of Public Health Interventions With the Epidemiology of the COVID-19 Outbreak in Wuhan, China. JAMA. 2020 Apr 10. | No primary data related to person-to-person transmission during asymptomatic, pre-symptomatic or post-symptomatic phases |
| Pan X., Chen D., Xia Y., Wu X., Li T., Ou X., Zhou L., Liu J. Asymptomatic cases in a family cluster with SARS-CoV-2 infection. The Lancet Infectious Diseases. 20 (4) (pp 410-411), 2020. Date of Publication: April 2020. | No primary data related to person-to-person transmission during asymptomatic, pre-symptomatic or post-symptomatic phases; the index patient had some symptomatic contact with both of the other patients and exposure histories were not reported fully |
| Pan XX; Chen Y; Wang AH; Wang JM; Ye LX; Gu SH; Fang T; Xu GZ. [Study on transmission dynamic of 15 clusters of coronavirus disease 2019 cases in Ningbo]. [Chinese] Chung-Hua Liu Hsing Ping Hsueh Tsa Chih Chinese Journal of Epidemiology. 41(0):E066, 2020 May 13. | No primary data related to person-to-person transmission during asymptomatic, pre-symptomatic or post-symptomatic phases |
| Pang L., Liu S., Zhang X., Tian T., Zhao Z. Transmission dynamics and control strategies of covid-19 in wuhan, china. Journal of Biological Systems. (no pagination), 2020. Article Number: 2020. Date of Publication: 2020. | No primary data related to person-to-person transmission during asymptomatic, pre-symptomatic or post-symptomatic phases; this was a modelling study that included some consideration of asymptomatic/pre-symptomatic transmission but did not report estimates of transmissibility directly |
| Park SW; Cornforth DM; Dushoff J; Weitz JS. The time scale of asymptomatic transmission affects estimates of epidemic potential in the COVID-19 outbreak. Epidemics. 31:100392, 2020 May 11. | No primary data related to person-to-person transmission during asymptomatic, pre-symptomatic or post-symptomatic phases; this was a modelling study that incorporated asymptomatic/presymptomatic transmission but only through scenarios based on assumed transmissibility and the impact on e.g. the basic reproduction number |
| Peak CM; Kahn R; Grad YH; Childs LM; Li R; Lipsitch M; Buckee CO. Individual quarantine versus active monitoring of contacts for the mitigation of COVID-19: a modelling study. The Lancet Infectious Diseases. 2020 May 20. | No primary data related to person-to-person transmission during asymptomatic, pre-symptomatic or post-symptomatic phases; this was a modelling study that incorporated asymptomatic/presymptomatic transmission but only through scenarios based on assumed transmissibility and the impact on e.g. the basic reproduction number; shorter serial intervals being suggestive of more presymptomatic transmission was, however, discussed |
| Peng L, Yang W, Zhang D, Zhuge C, Hong L Epidemic analysis of COVID-19 in China by dynamical modeling medRxiv, https://www.medrxiv.org/content/10.1101/2020.02.16.20023465v1 | No primary data related to person-to-person transmission during asymptomatic, pre-symptomatic or post-symptomatic phases; this was a modelling study that referred explicitly to asymptomatic/presymptomatic states but did not distinguish between asymptomatic/presymptomatic and symptomatic transmission |
| Peto J. Covid-19 mass testing facilities could end the epidemic rapidly. BMJ. 368:m1163, 2020 03 22. | No primary data related to person-to-person transmission during asymptomatic, pre-symptomatic or post-symptomatic phases |
| Phan L.T., Luong Q.C., Nguyen T.V., Nguyen H.T., Le H.Q., Nguyen T.T., Cao T.M., Pham Q.D. Importation and human-to-human transmission of a novel coronavirus in Vietnam. New England Journal of Medicine. 382 (9) (pp 872-874), 2020. Date of Publication: 27 Feb 2020. | No primary data related to person-to-person transmission during asymptomatic, pre-symptomatic or post-symptomatic phases; the index patient had some symptomatic contact with another patient |
| Pongpirul W.A., Pongpirul K., Ratnarathon A.C., Prasithsirikul W. Journey of a Thai Taxi driver and novel coronavirus. New England Journal of Medicine. 382 (11) (pp 1067-1068), 2020. Date of Publication: 12 Mar 2020. | No primary data related to person-to-person transmission during asymptomatic, pre-symptomatic or post-symptomatic phases; contact between taxi driver and family members did not result in transmission but exposure history of family members unclear and risk cannot be quantified (no denominator) |
| Prather KA; Wang CC; Schooley RT. Reducing transmission of SARS-CoV-2. Science. 2020 May 27. | No primary data related to person-to-person transmission during asymptomatic, pre-symptomatic or post-symptomatic phases |
| Qian GQ; Yang NB; Ding F; Ma AHY; Wang ZY; Shen YF; Shi CW; Lian X; Chu JG; Chen L; Wang ZY; Ren DW; Li GX; Chen XQ; Shen HJ; Chen XM. Epidemiologic and Clinical Characteristics of 91 Hospitalized Patients with COVID-19 in Zhejiang, China: A retrospective, multi-centre case series. Qjm. 2020 Mar 17. | No primary data related to person-to-person transmission during asymptomatic, pre-symptomatic or post-symptomatic phases |
| Qiu J. Covert coronavirus infections could be seeding new outbreaks. Nature. (no pagination), 2020. Date of Publication: 20 Mar 2020. | No primary data related to person-to-person transmission during asymptomatic, pre-symptomatic or post-symptomatic phases |
| Quilty BJ; Clifford S; Flasche S; Eggo RM; CMMID nCoV working group. Effectiveness of airport screening at detecting travellers infected with novel coronavirus (2019-nCoV). Euro Surveillance: Bulletin Europeen sur les Maladies Transmissibles = European Communicable Disease Bulletin. 25(5), 2020 02. | No primary data related to person-to-person transmission during asymptomatic, pre-symptomatic or post-symptomatic phases; this was a modelling study that incorporated asymptomatic/presymptomatic states but focusing on detection of infected people through screening, rather than transmissibility of infection |
| Rago Z; Szijjarto L; Duda E; Bella Z. Opportunity of periodic monitoring of COVID-19 patients, asymptomatic virus carriers, and postinfectious individuals with IgM/IgG rapid antibody tests among healthcare workers during SARS-CoV-2 pandemic. [Hungarian] A COVID-19-betegek, tunetmentes hordozok, illetve a betegsegen mar atesettek periodikus monitorizalasi lehetosege IgM/IgG antitest alapu gyorstesztekkel az egeszsegugyi szemelyzet koreben a SARS-CoV-2-jarvany idejen. <A COVID-19-betegek, tunetmentes hordozok, illetve a betegsegen mar atesettek periodikus monitorizalasi lehetosege IgM/IgG antitest alapu gyorstesztekkel az egeszsegugyi szemelyzet koreben a SARS-CoV-2-jarvany idejen.> Orvosi Hetilap. 161(21):854-860, 2020 05. | No primary data related to person-to-person transmission during asymptomatic, pre-symptomatic or post-symptomatic phases |
| Rahimi F; Talebi Bezmin Abadi A. Challenges of managing the asymptomatic carriers of SARS-CoV-2. Travel Medicine & Infectious Disease. 101677, 2020 Apr 18. | No primary data related to person-to-person transmission during asymptomatic, pre-symptomatic or post-symptomatic phases |
| Read JM, Bridgen JRE, Cummings DAT, Ho A, Jewell CP Novel coronavirus 2019-nCoV: early estimation of epidemiological parameters and epidemic predictions medRxiv, https://doi.org/10.1101/2020.01.23.20018549 | No primary data related to person-to-person transmission during asymptomatic, pre-symptomatic or post-symptomatic phases; this was a modelling study that did not incorporate asymptomatic/presymptomatic transmission |
| Riou J; Althaus CL. Pattern of early human-to-human transmission of Wuhan 2019 novel coronavirus (2019-nCoV), December 2019 to January 2020. Euro Surveillance: Bulletin Europeen sur les Maladies Transmissibles = European Communicable Disease Bulletin. 25(4), 2020 01. | No primary data related to person-to-person transmission during asymptomatic, pre-symptomatic or post-symptomatic phases; this was a modelling study that did not incorporate asymptomatic/presymptomatic transmission |
| Rivett L; Sridhar S; Sparkes D; Routledge M; Jones NK; Forrest S; Young J; Pereira-Dias J; Hamilton WL; Ferris M; Torok ME; Meredith L; CITIID-NIHR COVID-19 BioResource Collaboration; Curran MD; Fuller S; Chaudhry A; Shaw A; Samworth RJ; Bradley JR; Dougan G; Smith KGC; Lehner PJ; Matheson NJ; Wright G; Goodfellow IG; Baker S; Weekes MP. Screening of healthcare workers for SARS-CoV-2 highlights the role of asymptomatic carriage in COVID-19 transmission. eLife. 9, 2020 May 11. | No primary data related to person-to-person transmission during asymptomatic, pre-symptomatic or post-symptomatic phases |
| Rong XM; Yang L; Chu HD; Fan M. Effect of delay in diagnosis on transmission of COVID-19. Mathematical Biosciences & Engineering: MBE. 17(3):2725-2740, 2020 03 11. | No primary data related to person-to-person transmission during asymptomatic, pre-symptomatic or post-symptomatic phases; this was a modelling study that incorporated asymptomatic/presymptomatic transmission by equating transmission during the incubation phase with asymptomatic/presymptomatic transmission; the relative transmissibility of asymptomatic/presymptomatic and symptomatic states was not estimated |
| Roxby AC; Greninger AL; Hatfield KM; Lynch JB; Dellit TH; James A; Taylor J; Page LC; Kimball A; Arons M; Munanga A; Stone N; Jernigan JA; Reddy SC; Lewis J; Cohen SA; Jerome KR; Duchin JS; Neme S. Outbreak Investigation of COVID-19 Among Residents and Staff of an Independent and Assisted Living Community for Older Adults in Seattle, Washington. JAMA Internal Medicine. 2020 May 21. | No primary data related to person-to-person transmission during asymptomatic, pre-symptomatic or post-symptomatic phases; same study/setting as Roxby 2020b^[80]^ |
| Roxby AC; Greninger AL; Hatfield KM; Lynch JB; Dellit TH; James A; Taylor J; Page LC; Kimball A; Arons M; Schieve LA; Munanga A; Stone N; Jernigan JA; Reddy SC; Lewis J; Cohen SA; Jerome KR; Duchin JS; Neme S. Detection of SARS-CoV-2 Among Residents and Staff Members of an Independent and Assisted Living Community for Older Adults - Seattle, Washington, 2020. MMWR - Morbidity & Mortality Weekly Report. 69(14):416-418, 2020 Apr 10. | No primary data related to person-to-person transmission during asymptomatic, pre-symptomatic or post-symptomatic phases; same study/setting as Roxby 2020a^[81]^ |
| Sanche S., Lin Y.T., Xu C., Romero-Severson E., Hengartner N., Ke R. High Contagiousness and Rapid Spread of Severe Acute Respiratory Syndrome Coronavirus 2. Emerging infectious diseases. 26 (7) (pp 1470-1477), 2020. Date of Publication: 01 Jul 2020. | No primary data related to person-to-person transmission during asymptomatic, pre-symptomatic or post-symptomatic phases; this was a modelling study that did not incorporate asymptomatic/presymptomatic transmission |
| Sarkodie SA; Owusu PA. Investigating the cases of novel coronavirus disease (COVID-19) in China using dynamic statistical techniques. Heliyon. 6(4):e03747, 2020 Apr. | No primary data related to person-to-person transmission during asymptomatic, pre-symptomatic or post-symptomatic phases |
| Schwierzeck V; Konig JC; Kuhn J; Mellmann A; Correa-Martinez CL; Omran H; Konrad M; Kaiser T; Kampmeier S. First reported nosocomial outbreak of severe acute respiratory syndrome coronavirus 2 (SARS-CoV-2) in a pediatric dialysis unit. Clinical Infectious Diseases. 2020 Apr 27. | No primary data related to person-to-person transmission during asymptomatic, pre-symptomatic or post-symptomatic phases; exposure to index case occurred during symptomatic phase |
| Shen M, Peng Z, Xiao Y, Zhang L Modelling the epidemic trend of the 2019 novel coronavirus outbreak in China bioRxiv, https://www.biorxiv.org/content/10.1101/2020.01.23.916726v1 | No primary data related to person-to-person transmission during asymptomatic, pre-symptomatic or post-symptomatic phases; this was a modelling study that did not incorporate asymptomatic/presymptomatic transmission |
| Shim E; Tariq A; Choi W; Lee Y; Chowell G. Transmission potential and severity of COVID-19 in South Korea. International Journal of Infectious Diseases. 93:339-344, 2020 Apr. | No primary data related to person-to-person transmission during asymptomatic, pre-symptomatic or post-symptomatic phases; this study included modelling that did not incorporate asymptomatic/presymptomatic transmission; it also reported several clusters of infection but the exposure histories of the patients was not fully reported |
| Simha A, Prasad RV, Narayana S A simple stochastic SIR model for COVID 19 infection dynamics for Karnataka: learning from Europe arxiv, https://arxiv.org/abs/2003.11920 | No primary data related to person-to-person transmission during asymptomatic, pre-symptomatic or post-symptomatic phases; this was a modelling study that did not incorporate asymptomatic/presymptomatic transmission |
| Sjodin H., Wilder-Smith A., Osman S., Farooq Z., Rocklov J. Only strict quarantine measures can curb the coronavirus disease (COVID-19) outbreak in Italy, 2020. Eurosurveillance. 25 (13) (no pagination), 2020. Date of Publication: 02 Apr 2020. | No primary data related to person-to-person transmission during asymptomatic, pre-symptomatic or post-symptomatic phases; this was a modelling study that incorporated asymptomatic/presymptomatic transmission but only through scenarios based on assumed proportions/durations of asymptomatic/presymptomatic infections and the impact on e.g. duration of quarantine needed to contain an outbreak |
| Song JY; Yun JG; Noh JY; Cheong HJ; Kim WJ. Covid-19 in South Korea - Challenges of Subclinical Manifestations. New England Journal of Medicine. 382(19):1858-1859, 2020 05 07. | No primary data related to person-to-person transmission during asymptomatic, pre-symptomatic or post-symptomatic phases; likely transmission chains were characterised by index patients who were symptomatic at some stage and exposure histories were not reported fully |
| Song QQ; Zhao H; Fang LQ; Liu W; Zheng C; Zhang Y. [Study on assessing early epidemiological parameters of COVID-19 epidemic in China]. [Chinese] Chung-Hua Liu Hsing Ping Hsueh Tsa Chih Chinese Journal of Epidemiology. 41(4):461-465, 2020 Apr 10. | No primary data related to person-to-person transmission during asymptomatic, pre-symptomatic or post-symptomatic phases; this study included modelling that incorporated asymptomatic/presymptomatic transmission but did not distinguish between asymptomatic/presymptomatic and symptomatic transmission in terms of the impact on e.g. the reproduction number |
| Stower H. Clinical and epidemiological characteristics of children with COVID-19. Nature Medicine. 26 (4) (pp 465), 2020. Date of Publication: 01 Apr 2020. | No primary data related to person-to-person transmission during asymptomatic, pre-symptomatic or post-symptomatic phases |
| Streinu-Cercel A. Sars-cov-2 in romania - situation update and containment strategies. GERMS. 10 (1) (pp 8), 2020. Date of Publication: 2020. | No primary data related to person-to-person transmission during asymptomatic, pre-symptomatic or post-symptomatic phases |
| Swerdlow D.L., Finelli L., Lipsitch M. The authors reply:. New England Journal of Medicine. 382 (19) (pp 1869-1870), 2020. Date of Publication: 07 May 2020. | No primary data related to person-to-person transmission during asymptomatic, pre-symptomatic or post-symptomatic phases |
| Tan YP; Tan BY; Pan J; Wu J; Zeng SZ; Wei HY. Epidemiologic and clinical characteristics of 10 children with coronavirus disease 2019 in Changsha, China. Journal of Clinical Virology. 127:104353, 2020 06. | No primary data related to person-to-person transmission during asymptomatic, pre-symptomatic or post-symptomatic phases; simultaneous exposure outside household initially and later symptomatic exposure within household in at least some cases |
| Tang B., Wang X., Li Q., Bragazzi N.L., Tang S., Xiao Y., Wu J. Estimation of the transmission risk of the 2019-nCoV and its implication for public health interventions. Journal of Clinical Medicine. 9 (2) (no pagination), 2020. Article Number: 462. Date of Publication: | No primary data related to person-to-person transmission during asymptomatic, pre-symptomatic or post-symptomatic phases; this was a modelling study that incorporated asymptomatic/presymptomatic transmission but only through scenarios based on assumed transmissibility and the impact on e.g. the basic reproduction number |
| Tang B; Bragazzi NL; Li Q; Tang S; Xiao Y; Wu J. An updated estimation of the risk of transmission of the novel coronavirus (2019-nCov). Infectious Disease Modelling. 5:248-255, 2020. | No primary data related to person-to-person transmission during asymptomatic, pre-symptomatic or post-symptomatic phases; this was a modelling study that did not incorporate asymptomatic/presymptomatic transmission |
| Tang SY; Xiao YN; Peng ZH; Shen HB. [Prediction modeling with data fusion and prevention strategy analysis for the COVID-19 outbreak]. [Chinese] Chung-Hua Liu Hsing Ping Hsueh Tsa Chih Chinese Journal of Epidemiology. 41(4):480-484, 2020 Apr 10. | No primary data related to person-to-person transmission during asymptomatic, pre-symptomatic or post-symptomatic phases; this was a modelling study that did not incorporate asymptomatic/presymptomatic transmission |
| Tariq A; Lee Y; Roosa K; Blumberg S; Yan P; Ma S; Chowell G. Real-time monitoring the transmission potential of COVID-19 in Singapore, March 2020. BMC Medicine. 18(1):166, 2020 06 03. | No primary data related to person-to-person transmission during asymptomatic, pre-symptomatic or post-symptomatic phases; multiple clusters were identified but exposure histories were not reported fully and the risk cannot be quantified (no denominator) |
| Thompson RN. Novel Coronavirus Outbreak in Wuhan, China, 2020: Intense Surveillance Is Vital for Preventing Sustained Transmission in New Locations. Journal of Clinical Medicine. 9(2), 2020 Feb 11. | No primary data related to person-to-person transmission during asymptomatic, pre-symptomatic or post-symptomatic phases; this was a modelling study that did not incorporate asymptomatic/presymptomatic transmission |
| Thompson RN; Lovell-Read FA; Obolski U. Time from Symptom Onset to Hospitalisation of Coronavirus Disease 2019 (COVID-19) Cases: Implications for the Proportion of Transmissions from Infectors with Few Symptoms. Journal of Clinical Medicine. 9(5), 2020 May 01. | No primary data related to person-to-person transmission during asymptomatic, pre-symptomatic or post-symptomatic phases |
| Tindale L, Coombe M, Stockdale JE, Garlock E, Lau WYV, Saraswat M, Lee Y-HB, Zhang L, Chen D, Wallinga J, Colijn C Transmission interval estimates suggest pre-symptomatic spread of COVID-19 medRxiv, https://doi.org/10.1101/2020.03.03.20029983 | No primary data related to person-to-person transmission during asymptomatic, pre-symptomatic or post-symptomatic phases; serial intervals being short in relation to incubation periods and therefore suggesting presymptomatic transmission was, however, discussed |
| Tobolowsky FA; Gonzales E; Self JL; Rao CY; Keating R; Marx GE; McMichael TM; Lukoff MD; Duchin JS; Huster K; Rauch J; McLendon H; Hanson M; Nichols D; Pogosjans S; Fagalde M; Lenahan J; Maier E; Whitney H; Sugg N; Chu H; Rogers J; Mosites E; Kay M. COVID-19 Outbreak Among Three Affiliated Homeless Service Sites - King County, Washington, 2020. MMWR - Morbidity & Mortality Weekly Report. 69(17):523-526, 2020 May 01. | No primary data related to person-to-person transmission during asymptomatic, pre-symptomatic or post-symptomatic phases; possible overlap with Mosites 2020^[82]^ in terms of study settings/participants |
| Tuite A.R., Bogoch I.I., Sherbo R., Watts A., Fisman D., Khan K. Estimation of Coronavirus Disease 2019 (COVID-19) Burden and Potential for International Dissemination of Infection From Iran. Annals of internal medicine. 172 (10) (pp 699-701), 2020. Date of Publication: 19 May 2020. | No primary data related to person-to-person transmission during asymptomatic, pre-symptomatic or post-symptomatic phases |
| Tuite AR; Fisman DN; Greer AL. Mathematical modelling of COVID-19 transmission and mitigation strategies in the population of Ontario, Canada. CMAJ Canadian Medical Association Journal. 2020 Apr 08. | No primary data related to person-to-person transmission during asymptomatic, pre-symptomatic or post-symptomatic phases; this was a modelling study that incorporated asymptomatic/presymptomatic transmission but only through scenarios based on assumed transmissibility and the impact on e.g. the proportion of the population that would become infected |
| Uddin M; Mustafa F; Rizvi TA; Loney T; Suwaidi HA; Al-Marzouqi AHH; Eldin AK; Alsabeeha N; Adrian TE; Stefanini C; Nowotny N; Alsheikh-Ali A; Senok AC. SARS-CoV-2/COVID-19: Viral Genomics, Epidemiology, Vaccines, and Therapeutic Interventions. [Review] Viruses. 12(5), 2020 05 10. | No primary data related to person-to-person transmission during asymptomatic, pre-symptomatic or post-symptomatic phases |
| von Freyburg A; Hagedorn H; Brucher B; Schmidt A; Scherer MA. [COVID-19 cluster study at a teaching hospital]. [German] COVID-19-Cluster-Studie an einem Lehrkrankenhaus : Corona-Pandemie 2020. <COVID-19-Cluster-Studie an einem Lehrkrankenhaus : Corona-Pandemie 2020.> MMW Fortschritte der Medizin. 162(9):64-67, 2020 May. | No primary data related to person-to-person transmission during asymptomatic, pre-symptomatic or post-symptomatic phases |
| Wallace M; Hagan L; Curran KG; Williams SP; Handanagic S; Bjork A; Davidson SL; Lawrence RT; McLaughlin J; Butterfield M; James AE; Patil N; Lucas K; Hutchinson J; Sosa L; Jara A; Griffin P; Simonson S; Brown CM; Smoyer S; Weinberg M; Pattee B; Howell M; Donahue M; Hesham S; Shelley E; Philips G; Selvage D; Staley EM; Lee A; Mannell M; McCotter O; Villalobos R; Bell L; Diedhiou A; Ortbahn D; Clayton JL; Sanders K; Cranford H; Barbeau B; McCombs KG; Holsinger C; Kwit NA; Pringle JC; Kariko S; Strick L; Allord M; Tillman C; Morrison A; Rowe D; Marlow M. COVID-19 in Correctional and Detention Facilities - United States, February-April 2020. MMWR - Morbidity & Mortality Weekly Report. 69(19):587-590, 2020 May 15. | No primary data related to person-to-person transmission during asymptomatic, pre-symptomatic or post-symptomatic phases |
| Wan K.H., Huang S.S., Young A.L., Lam D.S.C. Precautionary measures needed for ophthalmologists during pandemic of the coronavirus disease 2019 (COVID-19). Acta Ophthalmologica. 98 (3) (pp 221-222), 2020. Date of Publication: 01 May 2020. | No primary data related to person-to-person transmission during asymptomatic, pre-symptomatic or post-symptomatic phases |
| Wan R; Mao ZQ; He LY; Hu YC; Wei-Chen. Evidence from two cases of asymptomatic infection with SARS-CoV-2: Are 14 days of isolation sufficient?. International Journal of Infectious Diseases. 95:174-175, 2020 Apr 03. | No primary data related to person-to-person transmission during asymptomatic, pre-symptomatic or post-symptomatic phases |
| Wang HQ. [Comparison of epidemiological and clinical characteristics of SARS, pandemic (H1N1) 2009 and COVID-19, and consideration on prevention and control strategies of COVID-19]. [Chinese] Chung-Hua Yu Fang i Hsueh Tsa Chih [Chinese Journal of Preventive Medicine]. 54(0):E033, 2020 Apr 27. | No primary data related to person-to-person transmission during asymptomatic, pre-symptomatic or post-symptomatic phases |
| Wang J., Zhou M., Liu F. Reasons for healthcare workers becoming infected with novel coronavirus disease 2019 (COVID-19) in China. Journal of Hospital Infection. 105 (1) (pp 100-101), 2020. Date of Publication: May 2020. | No primary data related to person-to-person transmission during asymptomatic, pre-symptomatic or post-symptomatic phases |
| Wang K; Zhao S; Liao Y; Zhao T; Wang X; Zhang X; Jiao H; Li H; Yin Y; Wang MH; Xiao L; Wang L; He D. Estimating the serial interval of the novel coronavirus disease (COVID-19) based on the public surveillance data in Shenzhen, China from January 19 to February 22, 2020. Transboundary & Emerging Diseases. 2020 May 26. | No primary data related to person-to-person transmission during asymptomatic, pre-symptomatic or post-symptomatic phases; there was a suggestion that asymptomatic/presymptomatic transmission occurred but exposure histories were not reported fully and the risk cannot be quantified (no denominator); negative serial intervals suggesting presymptomatic transmission were, however, discussed |
| Wang L, Zhou Y, He J, Zhu B, Wang F, Tang L, Eisenberg M, Song PXK An epidemiological forecast model and software assessing interventions on COVID-19 epidemic in China medRxiv, https://www.medrxiv.org/content/10.1101/2020.02.29.20029421v1 | No primary data related to person-to-person transmission during asymptomatic, pre-symptomatic or post-symptomatic phases; this was a modelling study that did not incorporate asymptomatic/presymptomatic transmission |
| Wang L; Duan Y; Zhang W; Liang J; Xu J; Zhang Y; Wu C; Xu Y; Li H. Epidemiologic and Clinical Characteristics of 26 Cases of COVID-19 Arising from Patient-to-Patient Transmission in Liaocheng, China. Clinical Epidemiology. 12:387-391, 2020. | No primary data related to person-to-person transmission during asymptomatic, pre-symptomatic or post-symptomatic phases; correction also excluded |
| Wang X; Zhou Q; He Y; Liu L; Ma X; Wei X; Jiang N; Liang L; Zheng Y; Ma L; Xu Y; Yang D; Zhang J; Yang B; Jiang N; Deng T; Zhai B; Gao Y; Liu W; Bai X; Pan T; Wang G; Chang Y; Zhang Z; Shi H; Ma WL; Gao Z. Nosocomial Outbreak of 2019 Novel Coronavirus Pneumonia in Wuhan, China. European Respiratory Journal. 2020 May 04. | No primary data related to person-to-person transmission during asymptomatic, pre-symptomatic or post-symptomatic phases |
| Wang Y; Kang H; Liu X; Tong Z. Asymptomatic cases with SARS-CoV-2 infection. Journal of Medical Virology. 2020 May 08. | No primary data related to person-to-person transmission during asymptomatic, pre-symptomatic or post-symptomatic phases |
| Wang Y; Liu Y; Liu L; Wang X; Luo N; Li L. Clinical Outcomes in 55 Patients With Severe Acute Respiratory Syndrome Coronavirus 2 Who Were Asymptomatic at Hospital Admission in Shenzhen, China. Journal of Infectious Diseases. 221(11):1770-1774, 2020 05 11. | No primary data related to person-to-person transmission during asymptomatic, pre-symptomatic or post-symptomatic phases |
| Wang Y; Tong J; Qin Y; Xie T; Li J; Li J; Xiang J; Cui Y; Higgs ES; Xiang J; He Y. Characterization of an asymptomatic cohort of SARS-COV-2 infected individuals outside of Wuhan, China. Clinical Infectious Diseases. 2020 May 22. | No primary data related to person-to-person transmission during asymptomatic, pre-symptomatic or post-symptomatic phases |
| Wang Y; Wang Y; Chen Y; Qin Q. Unique epidemiological and clinical features of the emerging 2019 novel coronavirus pneumonia (COVID-19) implicate special control measures. [Review] Journal of Medical Virology. 2020 Mar 05. | No primary data related to person-to-person transmission during asymptomatic, pre-symptomatic or post-symptomatic phases |
| Wang Z; Ma W; Zheng X; Wu G; Zhang R. Household transmission of SARS-CoV-2. Journal of Infection. 2020 Apr 10. | No primary data related to person-to-person transmission during asymptomatic, pre-symptomatic or post-symptomatic phases |
| Wangping J., Ke H., Yang S., Wenzhe C., Shengshu W., Shanshan Y., Jianwei W., Fuyin K., Penggang T., Jing L., Miao L., Yao H. Extended SIR Prediction of the Epidemics Trend of COVID-19 in Italy and Compared With Hunan, China. Frontiers in Medicine. 7 (no pagination), 2020. Article Number: 169. Date of Publication: 06 May 2020. | No primary data related to person-to-person transmission during asymptomatic, pre-symptomatic or post-symptomatic phases; this was a modelling study that did not incorporate asymptomatic/presymptomatic transmission |
| Wee LE; Sim XYJ; Conceicao EP; Aung MK; Goh JQ; Yeo DWT; Gan WH; Chua YY; Wijaya L; Tan TT; Tan BH; Ling ML; Venkatachalam I. Containment of COVID-19 cases among healthcare workers: The role of surveillance, early detection, and outbreak management. Infection Control & Hospital Epidemiology. 1-7, 2020 May 11. | No primary data related to person-to-person transmission during asymptomatic, pre-symptomatic or post-symptomatic phases |
| Wei XS; Wang XR; Zhang JC; Yang WB; Ma WL; Yang BH; Jiang NC; Gao ZC; Shi HZ; Zhou Q. A cluster of health care workers with COVID-19 pneumonia caused by SARS-CoV-2. Journal of Microbiology, Immunology & Infection. 2020 Apr 27. | Exposure history of health care workers incomplete; possibility of exposure during symptomatic phase |
| Weng L., Jiao Y., Li Y. First case of covid-19 in the United States. New England Journal of Medicine. 382 (19) (no pagination), 2020. Article Number: e53. Date of Publication: 07 May 2020. | No primary data related to person-to-person transmission during asymptomatic, pre-symptomatic or post-symptomatic phases |
| Wong J; Abdul Aziz ABZ; Chaw L; Mahamud A; Griffith MM; Ying-Ru LO; Naing L. High proportion of asymptomatic and presymptomatic COVID-19 infections in travelers and returning residents to Brunei. Journal of Travel Medicine. 2020 May 05. | No primary data related to person-to-person transmission during asymptomatic, pre-symptomatic or post-symptomatic phases; there was a suggestion that asymptomatic/presymptomatic transmission had occurred but exposure histories were not reported fully and the risk cannot be quantified (no denominator) |
| Wong SC; Kwong RT; Wu TC; Chan JWM; Chu MY; Lee SY; Wong HY; Lung DC. Risk of nosocomial transmission of coronavirus disease 2019: an experience in a general ward setting in Hong Kong. Journal of Hospital Infection. 2020 Apr 04. | No primary data related to person-to-person transmission during asymptomatic, pre-symptomatic or post-symptomatic phases |
| Wu J; Huang Y; Tu C; Bi C; Chen Z; Luo L; Huang M; Chen M; Tan C; Wang Z; Wang K; Liang Y; Huang J; Zheng X; Liu J. Household Transmission of SARS-CoV-2, Zhuhai, China, 2020. Clinical Infectious Diseases. 2020 May 11. | No primary data related to person-to-person transmission during asymptomatic, pre-symptomatic or post-symptomatic phases |
| Wu JT; Leung K; Leung GM. Nowcasting and forecasting the potential domestic and international spread of the 2019-nCoV outbreak originating in Wuhan, China: a modelling study. Lancet. 395(10225):689-697, 2020 02 29. | No primary data related to person-to-person transmission during asymptomatic, pre-symptomatic or post-symptomatic phases; this was a modelling study that did not incorporate asymptomatic/presymptomatic transmission |
| Wu P; Hao X; Lau EHY; Wong JY; Leung KSM; Wu JT; Cowling BJ; Leung GM. Real-time tentative assessment of the epidemiological characteristics of novel coronavirus infections in Wuhan, China, as at 22 January 2020. Euro Surveillance: Bulletin Europeen sur les Maladies Transmissibles = European Communicable Disease Bulletin. 25(3), 2020 Jan. | No primary data related to person-to-person transmission during asymptomatic, pre-symptomatic or post-symptomatic phases |
| Wu ZY. [Asymptomatic and pre-symptomatic cases of COVID-19 contribution to spreading the epidemic and need for targeted control strategies]. [Chinese] Chung-Hua Liu Hsing Ping Hsueh Tsa Chih Chinese Journal of Epidemiology. 41(00):E036, 2020 04 10. | No primary data related to person-to-person transmission during asymptomatic, pre-symptomatic or post-symptomatic phases |
| Xia XY; Wu J; Liu HL; Xia H; Jia B; Huang WX. Epidemiological and initial clinical characteristics of patients with family aggregation of COVID-19. Journal of Clinical Virology. 127:104360, 2020 06. | No primary data related to person-to-person transmission during asymptomatic, pre-symptomatic or post-symptomatic phases; the index patient had symptomatic contact with several of the other patients and exposure histories were not reported fully |
| Xiong Y; Song S; Ye G; Wang X. Family cluster of three recovered cases of pneumonia due to severe acute respiratory syndrome coronavirus 2 infection. BMJ Case Reports. 13(5), 2020 May 04. | No primary data related to person-to-person transmission during asymptomatic, pre-symptomatic or post-symptomatic phases; the index patient had some symptomatic contact with both of the other patients and exposure histories were not reported fully |
| Xu B., Kraemer M.U.G., Gutierrez B., Mekaru S., Sewalk K., Loskill A., Wang L., Cohn E., Hill S., Zarebski A., Li S., Wu C.-H., Hulland E., Morgan J., Scarpino S., Brownstein J., Pybus O., Pigott D., Kraemer M. Open access epidemiological data from the COVID-19 outbreak. The Lancet Infectious Diseases. 20 (5) (pp 534), 2020. Date of Publication: May 2020. | No primary data related to person-to-person transmission during asymptomatic, pre-symptomatic or post-symptomatic phases |
| Xu T; Huang R; Zhu L; Wang J; Cheng J; Zhang B; Zhao H; Chen K; Shao H; Zhu C; Wu C; Liu L. Epidemiological and clinical features of asymptomatic patients with SARS-CoV-2 infection. Journal of Medical Virology. 2020 Apr 28. | No primary data related to person-to-person transmission during asymptomatic, pre-symptomatic or post-symptomatic phases |
| Yamahata Y; Shibata A. Preparation for Quarantine on the Cruise Ship Diamond Princess in Japan due to COVID-19. JMIR Public Health and Surveillance. 6(2):e18821, 2020 05 11. | No primary data related to person-to-person transmission during asymptomatic, pre-symptomatic or post-symptomatic phases |
| Yang CY; Wang J. A mathematical model for the novel coronavirus epidemic in Wuhan, China. Mathematical Biosciences & Engineering: MBE. 17(3):2708-2724, 2020 03 11. | No primary data related to person-to-person transmission during asymptomatic, pre-symptomatic or post-symptomatic phases; this was a modelling study that incorporated asymptomatic/presymptomatic transmission by equating transmission during the incubation phase with asymptomatic/presymptomatic transmission; the relative transmissibility of asymptomatic/presymptomatic and symptomatic states was not estimated |
| Yang HY; Duan GC. [Analysis on the epidemic factors for the Corona Virus Disease]. [Chinese] Chung-Hua Yu Fang i Hsueh Tsa Chih [Chinese Journal of Preventive Medicine]. 54(0):E021, 2020 Mar 03. | No primary data related to person-to-person transmission during asymptomatic, pre-symptomatic or post-symptomatic phases |
| Yang Z., Zeng Z., Wang K., Wong S.-S., Liang W., Zanin M., Liu P., Cao X., Gao Z., Mai Z., Liang J., Liu X., Li S., Li Y., Ye F., Guan W., Yang Y., Li F., Luo S., Xie Y., Liu B., Wang Z., Zhang S., Wang Y., Zhong N., He J. Modified SEIR and AI prediction of the epidemics trend of COVID-19 in China under public health interventions. Journal of Thoracic Disease. 12 (3) (pp 165-174), 2020. Date of Publication: 01 Mar 2020. | No primary data related to person-to-person transmission during asymptomatic, pre-symptomatic or post-symptomatic phases; this was a modelling study that incorporated asymptomatic/presymptomatic transmission by equating transmission during the incubation phase with asymptomatic/presymptomatic transmission; differences in transmissibility between asymptomatic/presymptomatic and symptomatic phases depended only on different numbers of contacts with other people during these phases (i.e. underlying transmissibility during the different phases was assumed to be equal) |
| Ye LX; Wang HB; Lu HC; Chen BB; Zhu YY; Gu SH; Wang JM; Pan XX; Fang T; Dong HJ. [Investigation of a cluster epidemic of COVID-19 in Ningbo]. [Chinese] Chung-Hua Liu Hsing Ping Hsueh Tsa Chih Chinese Journal of Epidemiology. 41(0):E065, 2020 May 13. | No primary data related to person-to-person transmission during asymptomatic, pre-symptomatic or post-symptomatic phases; the index patient had symptomatic contact with at least some of the other patients and exposure histories were not reported fully |
| Yin S; Peng Y; Ren Y; Hu M; Tang L; Xiang Z; Li X; Wang M; Wang W. The implications of preliminary screening and diagnosis: Clinical characteristics of 33 mild patients with SARS-CoV-2 infection in Hunan, China. Journal of Clinical Virology. 128:104397, 2020 Apr 30. | No primary data related to person-to-person transmission during asymptomatic, pre-symptomatic or post-symptomatic phases |
| You C; Deng Y; Hu W; Sun J; Lin Q; Zhou F; Pang CH; Zhang Y; Chen Z; Zhou XH. Estimation of the time-varying reproduction number of COVID-19 outbreak in China. International Journal of Hygiene & Environmental Health. 228:113555, 2020 May 11. | No primary data related to person-to-person transmission during asymptomatic, pre-symptomatic or post-symptomatic phases; this was a modelling study that acknowledged the possibility of asymptomatic/pre-symptomatic transmission but did not distinguish between these and symptomatic transmission; negative serial intervals suggesting presymptomatic transmission were, however, discussed |
| Yousefpour A; Jahanshahi H; Bekiros S. Optimal policies for control of the novel coronavirus (COVID-19). Chaos Solitons & Fractals. 109883, 2020 May 16. | No primary data related to person-to-person transmission during asymptomatic, pre-symptomatic or post-symptomatic phases; this was a modelling study that acknowledged the possibility of asymptomatic/pre-symptomatic transmission but did not distinguish between these and symptomatic transmission |
| Yu X; Yang R. COVID-19 transmission through asymptomatic carriers is a challenge to containment. Influenza & Other Respiratory Viruses. 2020 Apr 04. | Contact between released prisoner and family member may have resulted in asymptomatic/presymptomatic transmission but exposure history of family member unclear and risk cannot be quantified (no denominator) |
| Yuan J., Li M., Lv G., Lu Z.K. Monitoring transmissibility and mortality of COVID-19 in Europe. International Journal of Infectious Diseases. 95 (pp 311-315), 2020. Date of Publication: June 2020. | No primary data related to person-to-person transmission during asymptomatic, pre-symptomatic or post-symptomatic phases |
| Zhang J; Litvinova M; Wang W; Wang Y; Deng X; Chen X; Li M; Zheng W; Yi L; Chen X; Wu Q; Liang Y; Wang X; Yang J; Sun K; Longini IM Jr; Halloran ME; Wu P; Cowling BJ; Merler S; Viboud C; Vespignani A; Ajelli M; Yu H. Evolving epidemiology and transmission dynamics of coronavirus disease 2019 outside Hubei province, China: a descriptive and modelling study. The Lancet Infectious Diseases. 2020 Apr 02. | No primary data related to person-to-person transmission during asymptomatic, pre-symptomatic or post-symptomatic phases |
| Zhang JZ; Zhou P; Han DB; Wang WC; Cui C; Zhou R; Xu KX; Liu L; Wang XH; Bai XH; Jiang XK. [Investigation on a cluster epidemic of COVID-19 in a supermarket in Liaocheng, Shandong province]. [Chinese] Chung-Hua Liu Hsing Ping Hsueh Tsa Chih Chinese Journal of Epidemiology. 41(0):E055, 2020 Apr 27. | No primary data related to person-to-person transmission during asymptomatic, pre-symptomatic or post-symptomatic phases; exposure histories were not reported fully |
| Zhang S; Diao M; Yu W; Pei L; Lin Z; Chen D. Estimation of the reproductive number of novel coronavirus (COVID-19) and the probable outbreak size on the Diamond Princess cruise ship: A data-driven analysis. International Journal of Infectious Diseases. 93:201-204, 2020 Apr. | No primary data related to person-to-person transmission during asymptomatic, pre-symptomatic or post-symptomatic phases; this was a modelling study that did not distinguish between asymptomatic/presymptomatic and symptomatic transmission |
| Zhang X. Epidemiology of Covid-19. The New England journal of medicine. 382 (19) (pp 1869), 2020. Date of Publication: 07 May 2020. | No primary data related to person-to-person transmission during asymptomatic, pre-symptomatic or post-symptomatic phases |
| Zhang Y; Li Y; Wang L; Li M; Zhou X. Evaluating Transmission Heterogeneity and Super-Spreading Event of COVID-19 in a Metropolis of China. International Journal of Environmental Research & Public Health [Electronic Resource]. 17(10), 2020 05 24. | No primary data related to person-to-person transmission during asymptomatic, pre-symptomatic or post-symptomatic phases; there was a suggestion that asymptomatic/presymptomatic transmission could occur but exposure histories were not reported fully and the risk cannot be quantified (no denominator) |
| Zhao S; Chen H. Modeling the epidemic dynamics and control of COVID-19 outbreak in China. Quantitative Biology. 1-9, 2020 Mar 11. | No primary data related to person-to-person transmission during asymptomatic, pre-symptomatic or post-symptomatic phases; this was a modelling study that acknowledged the possibility of asymptomatic/pre-symptomatic transmission but did not distinguish between these and symptomatic transmission |
| Zhao S; Lin Q; Ran J; Musa SS; Yang G; Wang W; Lou Y; Gao D; Yang L; He D; Wang MH. Preliminary estimation of the basic reproduction number of novel coronavirus (2019-nCoV) in China, from 2019 to 2020: A data-driven analysis in the early phase of the outbreak. International Journal of Infectious Diseases. 92:214-217, 2020 Mar. | No primary data related to person-to-person transmission during asymptomatic, pre-symptomatic or post-symptomatic phases; this was a modelling study that did not distinguish between asymptomatic/presymptomatic and symptomatic transmission |
| Zhao Z; Li X; Liu F; Zhu G; Ma C; Wang L. Prediction of the COVID-19 spread in African countries and implications for prevention and control: A case study in South Africa, Egypt, Algeria, Nigeria, Senegal and Kenya. Science of the Total Environment. 729:138959, 2020 Apr 25. | No primary data related to person-to-person transmission during asymptomatic, pre-symptomatic or post-symptomatic phases; this was a modelling study that did not incorporate asymptomatic/presymptomatic transmission |
| Zhen-Dong Y; Gao-Jun Z; Run-Ming J; Zhi-Sheng L; Zong-Qi D; Xiong X; Guo-Wei S. Clinical and transmission dynamics characteristics of 406 children with coronavirus disease 2019 in China: A review. [Review] Journal of Infection. 2020 Apr 28. | No primary data related to person-to-person transmission during asymptomatic, pre-symptomatic or post-symptomatic phases |
| Zhou T; Liu Q; Yang Z; Liao J; Yang K; Bai W; Lu X; Zhang W. Preliminary prediction of the basic reproduction number of the Wuhan novel coronavirus 2019-nCoV. Journal of Evidence-based Medicine. 13(1):3-7, 2020 Feb. | No primary data related to person-to-person transmission during asymptomatic, pre-symptomatic or post-symptomatic phases; this was a modelling study that did not incorporate asymptomatic/presymptomatic transmission |
| Zhu N; Li C; Ning SS; Chen S; Cao L; Yang GJ; Li XX; Nian YP; Wang WH; Liu YZ; Wang L; Lei FL; Zhang Y; Zhuang GH. [Epidemiological characteristics of COVID-19 in Shaanxi province]. [Chinese] Chung-Hua Liu Hsing Ping Hsueh Tsa Chih Chinese Journal of Epidemiology. 41(0):E034, 2020 Apr 03. | No primary data related to person-to-person transmission during asymptomatic, pre-symptomatic or post-symptomatic phases |
| Zhu Y; Chen YQ. On a Statistical Transmission Model in Analysis of the Early Phase of COVID-19 Outbreak. Statistics in Biosciences. 1-17, 2020 Apr 02. | No primary data related to person-to-person transmission during asymptomatic, pre-symptomatic or post-symptomatic phases; this was a modelling study that included some consideration of asymptomatic/pre-symptomatic transmission but did not report estimates of transmissibility directly |

## **Appendix F: Characteristics of included studies table**

**Table F.1** Characteristics of included studies

| **Study identifier** | **Study type** | **Country** | **Population** | **Exposure phase(s)/state(s) investigated** | **Comparative study** | **Transmission outcomes** | **Reviewer comments** |
| --- | --- | --- | --- | --- | --- | --- | --- |
| Bae 2020^[4]^ | Cluster/outbreak investigation (case study/series) | South Korea | People at risk of person-to-person transmission of SARS-CoV-2 in community setting | Presymptomatic | No | Quantitative assessment of SARS-CoV-2 transmission risk | None |
| Baettig 2020^[5]^ | Cluster/outbreak investigation (case study/series) | Switzerland | People at risk of person-to-person transmission of SARS-CoV-2 in community setting | Presymptomatic | No | Quantitative assessment of SARS-CoV-2 transmission risk | None |
| Bai 2020b^[6]^ | Cluster/outbreak investigation (case study/series) | China | People at risk of person-to-person transmission of SARS-CoV-2 in community setting | Asymptomatic | No | Qualitative assessment of SARS-CoV-2 transmission risk | None |
| Buonanno 2020^[8]^ | Mathematical modelling of epidemic spread | Italy | People at risk of person-to-person transmission of SARS-CoV-2 in indoor commercial environments | Asymptomatic  Symptomatic | Yes (asymptomatic transmission in different indoor environments) | Quantitative assessment of SARS-CoV-2 transmission risk | None |
| Cai 2020^[9]^ | Cluster/outbreak investigation (case study/series) | China | People at risk of person-to-person transmission of SARS-CoV-2 in community settings | Presymptomatic  Symptomatic | No | Qualitative assessment of SARS-CoV-2 transmission risk | None |
| Chan 2020^[10]^ | Cluster/outbreak investigation (case study/series) | China | People at risk of person-to-person transmission of SARS-CoV-2 in community settings | Presymptomatic  Symptomatic | No | Qualitative assessment of SARS-CoV-2 transmission risk | None |
| Chen 2020;^[11]^ He 2020a;^[19]^ Yin 2020^[52]^ | Relative transmissibility (nested case–control study) | China | People at risk of person-to-person transmission of SARS-CoV-2 in community and nosocomial settings | Asymptomatic  Symptomatic | Yes | Quantitative assessment of SARS-CoV-2 transmission risk | Multiple reports/analyses of same data |
| Cheng 2020^[12]^ | Relative transmissibility (nested case–control study) | Taiwan | People at risk of person-to-person transmission of SARS-CoV-2 in community and nosocomial settings | Presymptomatic  Asymptomatic  Symptomatic | Yes | Quantitative assessment of SARS-CoV-2 transmission risk | None |
| Ferretti 2020^[13]^ | Mathematical modelling of epidemic spread | Not applicable (generic setting – not focused on a specific country) | People at risk of person-to-person transmission of SARS-CoV-2 in any setting | Presymptomatic  Asymptomatic  Symptomatic | Yes | Quantitative assessment of SARS-CoV-2 transmission risk | None |
| Gao 2020a^[14]^ | Cluster/outbreak investigation (case study/series) | China | People at risk of person-to-person transmission of SARS-CoV-2 in nosocomial setting | Asymptomatic | No | Quantitative assessment of SARS-CoV-2 transmission risk | PPE in use |
| Gao 2020b^[15]^ | Cluster/outbreak investigation (case study/series) | China | People at risk of person-to-person transmission of SARS-CoV-2 in community settings | Presymptomatic | No | Qualitative assessment of SARS-CoV-2 transmission risk | None |
| Gatto 2020^[16]^ | Mathematical modelling of epidemic spread | Italy | People at risk of person-to-person transmission of SARS-CoV-2 in any setting | Susceptible  Exposed  Presymptomatic  Infected with severe symptoms  Asymptomatic or with mild symptoms  Hospitalised  In home quarantine  Recovered  Dead | Yes | Quantitative assessment of SARS-CoV-2 transmission risk | None |
| Guan 2020^[17]^ | Cluster/outbreak investigation (case study/series) | China | People at risk of person-to-person transmission of SARS-CoV-2 in community settings | Presymptomatic  Symptomatic | No | Qualitative assessment of SARS-CoV-2 transmission risk | None |
| Hamner 2020^[18]^ | Cluster/outbreak investigation (case study/series) | USA | People at risk of person-to-person transmission of SARS-CoV-2 in community setting | Presymptomatic  Symptomatic | No | Quantitative assessment of SARS-CoV-2 transmission risk | None |
| He 2020b^[20]^ | Cluster/outbreak investigation (case study/series) | China  Hong Kong  Japan  Malaysia  Singapore  Taiwan  USA  Vietnam | People at risk of person-to-person transmission of SARS-CoV-2, reporting of settings incomplete, but includes community settings | Presymptomatic  Symptomatic | No | Qualitative assessment of SARS-CoV-2 transmission risk | Cannot rule out overlap with other cluster/outbreak investigations |
| Hijnen 2020^[21]^ | Cluster/outbreak investigation (case study/series) | Italy | People at risk of person-to-person transmission of SARS-CoV-2 in community settings | Presymptomatic | No | Quantitative assessment of SARS-CoV-2 transmission risk | None |
| Hu 2020^[22]^ | Cluster/outbreak investigation (case study/series) | China | People at risk of person-to-person transmission of SARS-CoV-2 in community setting | Asymptomatic | No | Quantitative assessment of SARS-CoV-2 transmission risk | None |
| Huang 2020a^[23]^ | Cluster/outbreak investigation (case study/series) | China | People at risk of person-to-person transmission of SARS-CoV-2 in community settings | Presymptomatic | No | Quantitative assessment of SARS-CoV-2 transmission risk | None |
| Huang 2020b^[24]^ | Cluster/outbreak investigation (case study/series) | China | People at risk of person-to-person transmission of SARS-CoV-2 in community settings | Presymptomatic | No | Qualitative assessment of SARS-CoV-2 transmission risk | None |
| Kang 2020^[25]^ | Cluster/outbreak investigation (case study/series) | China | People at risk of person-to-person transmission of SARS-CoV-2 in community settings | Presymptomatic | No | Qualitative assessment of SARS-CoV-2 transmission risk | None |
| Li 2020a^[27]^ | Cluster/outbreak investigation (case study/series) | China | People at risk of person-to-person transmission of SARS-CoV-2 in community and nosocomial settings | Presymptomatic  Symptomatic | No | Qualitative assessment of SARS-CoV-2 transmission risk | None |
| Li 2020b^[28]^ | Cluster/outbreak investigation (case study/series) | China | People at risk of person-to-person transmission of SARS-CoV-2 in community setting | Presymptomatic | No | Qualitative assessment of SARS-CoV-2 transmission risk | None |
| Li 2020c^[29]^ | Mathematical modelling of epidemic spread | China | People at risk of person-to-person transmission of SARS-CoV-2 in any setting | Susceptible  Exposed  Documented  Infected (symptoms severe  enough to be confirmed/observed)  Undocumented infected (lacking symptoms severe enough to be confirmed/observed) | Yes | Quantitative assessment of SARS-CoV-2 transmission risk | None |
| Li 2020d^[30]^ | Cluster/outbreak investigation (case study/series) | China | People at risk of person-to-person transmission of SARS-CoV-2 in nosocomial setting | Presymptomatic | No | Qualitative assessment of SARS-CoV-2 transmission risk | None |
| Liu 2020b^[31]^ | Cluster/outbreak investigation (case study/series) | Taiwan | People at risk of person-to-person transmission of SARS-CoV-2 in community setting | Presymptomatic | No | Quantitative assessment of SARS-CoV-2 transmission risk | None |
| Lu 2020a^[33]^ | Cluster/outbreak investigation (case study/series) | China | People at risk of person-to-person transmission of SARS-CoV-2 in community settings | Presymptomatic | No | Qualitative assessment of SARS-CoV-2 transmission risk | None |
| Lu 2020b^[34]^ | Cluster/outbreak investigation (case study/series) | China | People at risk of person-to-person transmission of SARS-CoV-2 in community setting | Asymptomatic | No | Qualitative assessment of SARS-CoV-2 transmission risk | None |
| Park 2020^[35]^ | Relative transmissibility (nested case–control study) | South Korea | People at risk of person-to-person transmission of SARS-CoV-2 in community settings | Presymptomatic  Asymptomatic | Yes | Quantitative assessment of SARS-CoV-2 transmission risk | None |
| Pung 2020^[36]^ | Cluster/outbreak investigation (case study/series) | Singapore | People at risk of person-to-person transmission of SARS-CoV-2 in community settings | Unknown (presymptomatic or asymptomatic) | No | Qualitative assessment of SARS-CoV-2 transmission risk | Multiple clusters/outbreaks reported in same article; overlap with Yong 2020^[53]^ and Wei 2020^[46]^ summarised separately |
| Qian 2020^[37]^ | Cluster/outbreak investigation (case study/series) | China | People at risk of person-to-person transmission of SARS-CoV-2 in community settings | Presymptomatic or asymptomatic | No | Quantitative assessment of SARS-CoV-2 transmission risk | None |
| Qiu 2020a^[38]^ | Cluster/outbreak investigation (case study/series) | China | People at risk of person-to-person transmission of SARS-CoV-2 in community settings | Asymptomatic | No | Qualitative assessment of SARS-CoV-2 transmission risk | None |
| Qiu 2020b^[39]^ | Cluster/outbreak investigation (case study/series) | China | People at risk of person-to-person transmission of SARS-CoV-2 in community settings | Presymptomatic | No | Qualitative assessment of SARS-CoV-2 transmission risk | None |
| Ravaioli 2020^[40]^ | Cluster/outbreak investigation (case study/series) | Italy | People at risk of person-to-person transmission of SARS-CoV-2 in nosocomial setting | Presymptomatic | No | Quantitative assessment of SARS-CoV-2 transmission risk | PPE in use |
| Rothe 2020;^[41]^ Kupferschmidt 2020;^[26]^ Bohmer 2020^[7]^ | Cluster/outbreak investigation (case study/series) | Germany | People at risk of person-to-person transmission of SARS-CoV-2 in community setting | Presymptomatic or pauci-symptomatic | No | Qualitative assessment of SARS-CoV-2 transmission risk | Multiple reports of, or commentary on, same cluster/outbreak |
| Scott 2020^[42]^ | Cluster/outbreak investigation (case study/series) | USA | People at risk of person-to-person transmission of SARS-CoV-2 in community and nosocomial settings | Asymptomatic | No | Quantitative assessment of SARS-CoV-2 transmission risk | Some PPE in use |
| Song 2020^[43]^ | Cluster/outbreak investigation (case study/series) | China | People at risk of person-to-person transmission of SARS-CoV-2 in community setting | Presymptomatic | No | Qualitative assessment of SARS-CoV-2 transmission risk | None |
| Tong 2020^[44]^ | Cluster/outbreak investigation (case study/series) | China | People at risk of person-to-person transmission of SARS-CoV-2 in community settings | Presymptomatic | No | Qualitative assessment of SARS-CoV-2 transmission risk | None |
| Wan 2020^[45]^ | Mathematical modelling of epidemic spread | China | People at risk of person-to-person transmission of SARS-CoV-2 in any setting | Susceptible  Exposed  Infectious with symptoms  Infectious but asymptomatic  Isolated susceptible  Quarantined infected awaiting confirmation  Hospitalised  Recovered (including recovered after being hospitalised)  Dead | Yes | Quantitative assessment of SARS-CoV-2 transmission risk | None |
| Wei 2020^[46]^ | Cluster/outbreak investigation (case study/series) | Singapore | People at risk of person-to-person transmission of SARS-CoV-2 in community settings | Presymptomatic | No | Qualitative assessment of SARS-CoV-2 transmission risk | Multiple clusters/outbreaks reported in same article; overlap with Yong 2020^[53]^ and Pung 2020^[36]^ summarised separately |
| Wu 2020;^[47]^ Yang 2020;^[50]^ Liu 2020c^[32]^ | Cluster/outbreak investigation (case study/series) | China | People at risk of person-to-person transmission of SARS-CoV-2 in community setting | Presymptomatic  Symptomatic | No | Qualitative assessment of SARS-CoV-2 transmission risk | Multiple reports of same cluster/outbreak |
| Xia 2020^[48]^ | Cluster/outbreak investigation (case study/series) | China | People at risk of person-to-person transmission of SARS-CoV-2 in community settings | Presymptomatic | No | Qualitative assessment of SARS-CoV-2 transmission risk | None |
| Xiao 2020^[49]^ | Cluster/outbreak investigation (case study/series) | China | People at risk of person-to-person transmission of SARS-CoV-2 in community settings | Presymptomatic | No | Qualitative assessment of SARS-CoV-2 transmission risk | None |
| Ye 2020^[51]^ | Cluster/outbreak investigation (case study/series) | China | People at risk of person-to-person transmission of SARS-CoV-2 in community settings | Presymptomatic | No | Qualitative assessment of SARS-CoV-2 transmission risk | None |
| Yong 2020;^[53]^ Pung 2020;^[36]^ Wei 2020^[46]^ | Cluster/outbreak investigation (case study/series) | Singapore | People at risk of person-to-person transmission of SARS-CoV-2 in community setting | Presymptomatic | No | Qualitative assessment of SARS-CoV-2 transmission risk | Multiple reports of same cluster/outbreak |
| Yu 2020^[54]^ | Cluster/outbreak investigation (case study/series) | China | People at risk of person-to-person transmission of SARS-CoV-2 in community settings | Presymptomatic | No | Qualitative assessment of SARS-CoV-2 transmission risk | None |
| Zhang 2020a^[55]^ | Cluster/outbreak investigation (case study/series) | China | People at risk of person-to-person transmission of SARS-CoV-2 in community settings | Asymptomatic | No | Qualitative assessment of SARS-CoV-2 transmission risk | None |
| Zhang 2020b^[56]^ | Relative transmissibility (nested case–control study) | China | People at risk of person-to-person transmission of SARS-CoV-2 in community and nosocomial settings | Presymptomatic Asymptomatic | Yes | Quantitative assessment of SARS-CoV-2 transmission risk | Cannot rule out overlap with cluster/outbreak investigations |
| Zhang 2020c^[57]^ | Cluster/outbreak investigation (case study/series) | China | People at risk of person-to-person transmission of SARS-CoV-2 in community settings | Presymptomatic or pauci-symptomatic  Symptomatic | No | Qualitative assessment of SARS-CoV-2 transmission risk | None |
| Zhao 2020^[58]^ | Cluster/outbreak investigation (case study/series) | China | People at risk of person-to-person transmission of SARS-CoV-2 in community settings | Uncertain (probably presymptomatic) | No | Qualitative assessment of SARS-CoV-2 transmission risk | None |

Studies listed in alphabetical order; grey shading indicates consideration of evidence from outside mainland China
PPE personal protective equipment; SARS-CoV-2 severe acute respiratory syndrome coronavirus 2

## **Appendix G: Evidence tables**

**Table G.1** Case series reports investigating clusters/outbreaks and associated transmission chains

| **Study identifier** | **Index case(s)** | **Associated cluster/outbreak** | **Case identification and contact tracing** | **Exposure and precautions/controls** | **Transmission outcomes** | **Study quality^a^** | **Reviewer comments** |
| --- | --- | --- | --- | --- | --- | --- | --- |
| Cai 2020^[9]^ | **Source of infection**  Temporarily in Wuhan, Hubei province, China  **Date(s) of infection**  Not reported (returned to Wenzhou, Zhejiang province, China on 18 Dec 2019) | **Setting**  Community (shopping centre, office, and associated contacts)  **Location**  Wenzhou, Zhejiang province, China  **Earliest exposure to index case**  18 Dec 2019  **Latest follow up**  12 Feb 2020 | **Identification method**  SARS-CoV-2 RNA (RT-PCR)  **Contact tracing**  Extensive (shopping centre staff, office staff, customers, and their contacts)  **People tested**  Contacts of first symptomatic case and colleague, including index case, and their contacts | **Exposure phase(s) relative to index case’s infection**  Presymptomatic (days since index case was infected not calculable)  Symptomatic  **Precautions/controls used**  Closure of shopping centre | **Transmission demonstrated**  Uncertain (possibly presymptomatic)  **Attack rate (exposure via index case only)**  Not calculable (possibly at least 1 secondary infection resulting from presymptomatic exposure to index case) | **Clear inclusion criteria:** yes  **Condition measured in standard, reliable way:** yes  **Valid methods used to identify condition:** yes  **Consecutive inclusion:** not applicable  **Complete inclusion:** unclear  **Clear reporting of demographics:** yes  **Clear reporting of clinical information:** yes  **Clear reporting of outcomes and follow up:** yes  **Clear reporting of presenting site(s)/clinic(s) demographic information:** not applicable  **Statistical analysis appropriate:** not applicable | None |
| He 2020b^[20]^ | **Source of infection**  Not reported (77 transmission pairs investigated, therefore, 77 index cases)  **Date(s) of infection**  Not reported (on or before 18 Dec 2019 for the first transmission pair) | **Setting**  Not reported (mostly community (household or extended family) among transmission pairs)  **Location**  Mainland China (numbers of transmission pairs in parentheses):  Hefei, Anhui province (8); Nanyang, Henan province (10); Shaanxi province (19); Shenzhen, Guangdong province (10)  Other (numbers of transmission pairs in parentheses):  Chicago, Illinois, USA (1); Hong Kong (8); Japan (12); Malaysia (2); Singapore (1); Taiwan (4); Vietnam (2)  **Earliest exposure to index case**  18 Dec 2019 (across the 77 transmission pairs)  **Latest follow up**  5 Mar 2020 (across the 77 transmission pairs) | **Identification method**  Laboratory testing (no further details reported)  **Contact tracing**  No primary contact tracing as part of the study (information about transmission  pairs obtained from public domain sources, such as government and media communications)  **People tested**  Not reported (but transmission pairs reported as laboratory-confirmed cases) | **Exposure phase(s) relative to index case’s infection**  Presymptomatic (days since index case was infected not calculable) or symptomatic (depending on transmission pair)  **Precautions/controls used**  Home quarantine of some cases among transmission pairs | **Transmission demonstrated**  Uncertain (possibly presymptomatic)  **Attack rate (exposure via index case only)**  Not calculable (possibly at least 3 secondary infections resulting from presymptomatic exposure to 3 index cases, reported as ‘transmission pair 54’ (Nanyang, Henan province, China), ‘transmission pair 63’ (Shaanxi province, China) and ‘transmission pair 68’ (Shaanxi province, China)) | **Clear inclusion criteria:** yes  **Condition measured in standard, reliable way:** unclear  **Valid methods used to identify condition:** unclear  **Consecutive inclusion:** unclear  **Complete inclusion:** unclear  **Clear reporting of demographics:** yes  **Clear reporting of clinical information:** yes  **Clear reporting of outcomes and follow up:** yes  **Clear reporting of presenting site(s)/clinic(s) demographic information:** not applicable  **Statistical analysis appropriate:** not applicable | Serial interval and incubation period distributions (estimated from information about transmission pairs) and viral shedding profiles (relative to onset of symptoms in a separate sample of laboratory-confirmed cases) also reported, but not relevant to this review; no apparent overlap between ‘transmission pair 54’ (Nanyang, Henan province, China), ‘transmission pair 63’ (Shaanxi province, China) or ‘transmission pair 68’ (Shaanxi province, China) and clusters/outbreaks summarised elsewhere in this table, but cannot rule out overlap between other transmission pairs and clusters/outbreaks summarised elsewhere in the table, therefore summarised here in a single row |
| Chan 2020^[10]^ | **Source of infection**  Visiting relatives in Wuhan, Hubei province, China (possibly 2 index cases with common exposure)  **Date(s) of infection**  29 Dec 2019 to 4 Jan 2020 (returned to Shenzhen, Guangdong  province, China on 4 Jan 2020) | **Setting**  Community (household and family gatherings)  **Location**  Shenzhen, Guangdong  province, China  **Earliest exposure to index case**  29 Dec 2019  **Latest follow up**  15 Jan 2020 | **Identification method**  SARS-CoV-2 RNA (RT-PCR) or radiological (ground-glass appearance on CT scan of the lungs, 1 household member only)  **Contact tracing**  Limited (extended family only)  **People tested**  Index case’s household | **Exposure phase(s) relative to index case’s infection**  Presymptomatic (< 7 days since either index case was infected)  Symptomatic  **Precautions/controls used**  Some wearing of surgical masks by index case’s household while visiting relatives in Wuhan, Hubei province, China, including relative in hospital | **Transmission demonstrated**  Uncertain (possibly presymptomatic)  **Attack rate (exposure via index case only)**  Not calculable (possibly at least 3 secondary infections resulting from presymptomatic exposure to either index case) | **Clear inclusion criteria:** yes  **Condition measured in standard, reliable way:** yes  **Valid methods used to identify condition:** yes  **Consecutive inclusion:** not applicable  **Complete inclusion:** unclear  **Clear reporting of demographics:** yes  **Clear reporting of clinical information:** yes  **Clear reporting of outcomes and follow up:** yes  **Clear reporting of presenting site(s)/clinic(s) demographic information:** not applicable  **Statistical analysis appropriate:** not applicable | None |
| Li 2020d^[30]^ | **Source of infection**  Not reported (but investigation based in Wuhan, Hubei province, China)  **Date(s) of infection**  Not reported (but index case had ground-glass appearance on preoperative CT scan of the chest, 3 Jan 2020) | **Setting**  Nosocomial (unrecognised preoperative infection in thoracic surgery department)  **Location**  Wuhan, Hubei province, China  **Earliest exposure to index case**  3 Jan 2020 (or before)  **Latest follow up**  3 Mar 2020 | **Identification method**  SARS-CoV-2 RNA (RT-PCR) or clinical (symptoms suggesting infection) or radiological (ground-glass appearance on CT scan of the chest)  **Contact tracing**  Limited (exposure history investigated retrospectively for hospital patients/staff with confirmed infection)  **People tested**  Suspected cases | **Exposure phase(s) relative to index case’s infection**  Presymptomatic (days since index case was infected not calculable)  **Precautions/controls used**  Hospital quarantine of confirmed cases, closure of hospital to new admissions | **Transmission demonstrated**  Uncertain (possibly presymptomatic)  **Attack rate (exposure via index case only)**  Not calculable (possibly at least 3 secondary infections resulting from presymptomatic exposure to index case) | **Clear inclusion criteria:** yes  **Condition measured in standard, reliable way:** yes  **Valid methods used to identify condition:** yes  **Consecutive inclusion:** unclear  **Complete inclusion:** unclear  **Clear reporting of demographics:** yes  **Clear reporting of clinical information:** yes  **Clear reporting of outcomes and follow up:** yes  **Clear reporting of presenting site(s)/clinic(s) demographic information:** not applicable  **Statistical analysis appropriate:** /not applicable | Data extracted are for transmission chain associated with ‘patient 01’; no other transmission chains were reported in the article |
| Tong 2020^[44]^ | **Source of infection**  Living in Wuhan, Hubei province, China  **Date(s) of infection**  Not reported (travelled to Zhoushan, Zhejiang province, China on 5 Jan 2020) | **Setting**  Community (academic conference and associated households)  **Location**  Zhoushan, Zhejiang province, China  **Earliest exposure to index case**  5 Jan 2020  **Latest follow up**  1 Feb 2020 | **Identification method**  SARS-CoV-2 RNA (RT-PCR)  **Contact tracing**  Limited (households of 2 conference attendees who dined with index case)  **People tested**  Households of 2 conference attendees who dined with index case | **Exposure phase(s) relative to index case’s infection**  Presymptomatic (days since index case was infected not calculable)  **Precautions/controls used**  Hospital isolation of confirmed cases, home quarantine for other household members | **Transmission demonstrated**  Uncertain (possibly presymptomatic)  **Attack rate (exposure via index case only)**  Not calculable (possibly at least 1 secondary infection resulting from presymptomatic exposure to index case) | **Clear inclusion criteria:** yes  **Condition measured in standard, reliable way:** yes  **Valid methods used to identify condition:** yes  **Consecutive inclusion:** not applicable  **Complete inclusion:** unclear  **Clear reporting of demographics:** yes  **Clear reporting of clinical information:** yes  **Clear reporting of outcomes and follow up:** yes  **Clear reporting of presenting site(s)/clinic(s) demographic information:** not applicable  **Statistical analysis appropriate:** not applicable | None |
| *Bai 2020b^[6]^* | ***Source of infection***  *Living in Wuhan, Hubei province, China*  ***Date(s) of infection***  *Not reported (travelled to Anyang, Henan province, China on 10 Jan 2020)* | ***Setting***  *Community (family gatherings)*  ***Location***  *Anyang, Henan, province, China*  ***Earliest exposure to index case***  *10 Jan 2020*  ***Latest follow up***  *11 Feb 2020* | ***Identification method***  *SARS-CoV-2 RNA (RT-PCR)*  ***Contact tracing***  *Limited (extended family only)*  ***People tested***  *Extended family, including index case* | ***Exposure phase(s) relative to index case’s infection***  *Asymptomatic (no abnormalities on CT scan of the chest; days since index case was infected not calculable)*  ***Precautions/controls used***  *Isolation of index case for observation after relatives confirmed infected* | ***Transmission demonstrated***  *Uncertain (possibly asymptomatic)*  ***Attack rate (exposure via index case only)***  *Not calculable (possibly at least 1 secondary infection resulting from asymptomatic exposure to index case)* | ***Clear inclusion criteria:*** *yes*  ***Condition measured in standard, reliable way:*** *yes*  ***Valid methods used to identify condition:*** *yes*  ***Consecutive inclusion:*** *not applicable*  ***Complete inclusion:*** *unclear*  ***Clear reporting of demographics:*** *yes*  ***Clear reporting of clinical information:*** *yes*  ***Clear reporting of outcomes and follow up:*** *yes*  ***Clear reporting of presenting site(s)/clinic(s) demographic information:*** *not applicable*  ***Statistical analysis appropriate:*** *not applicable* | *None* |
| Xia 2020^[48]^ | **Source of infection**  Travelling to Wuhan, Hubei province, China, or having contact with people from  Wuhan, people with confirmed infection or people with respiratory symptoms (50 clusters/outbreaks investigated, therefore, 50 index cases)  **Date(s) of infection**  10 to 28 Jan 2020 (range across the 50 index cases) | **Setting**  Community (including household or extended family)  **Location**  China, excluding Hubei province  **Earliest exposure to index case**  10 Jan 2020  **Latest follow up**  16 Feb 2020 | **Identification method**  Not reported (possibly RT-PCR)  **Contact tracing**  Extensive (tracing process not reported, but number of clusters/outbreaks investigated suggests systematic approach to contact tracing)  **People tested**  Not reported (possibly symptomatic cases only) | **Exposure phase(s) relative to index case’s infection**  Presymptomatic (days since index case was infected not calculable)  **Precautions/controls used**  Quarantine of close contacts of symptomatic and test-positive cases | **Transmission demonstrated**  Uncertain (possibly presymptomatic)  **Attack rate (exposure via index case only)**  Not calculable (possibly at least 4 secondary infections resulting from presymptomatic exposure to 4 index cases, reported as ’cluster 5’, ‘cluster 21’, ‘cluster 29’, and ‘cluster 46’) | **Clear inclusion criteria:** yes  **Condition measured in standard, reliable way:** unclear  **Valid methods used to identify condition:** unclear  **Consecutive inclusion:** unclear  **Complete inclusion:** unclear  **Clear reporting of demographics:** yes  **Clear reporting of clinical information:** yes  **Clear reporting of outcomes and follow up:** yes  **Clear reporting of presenting site(s)/clinic(s) demographic information:** not applicable  **Statistical analysis appropriate:** not applicable | Estimated duration of incubation period also reported, from which proportion of presymptomatic transmission estimated; cannot rule out overlap with other clusters/outbreaks investigated in China, therefore summarised here in a single row |
| Kang 2020^[25]^ | **Source of infection**  Travelling via Wuhan, Hubei province, China (possibly 2 index cases with common exposure)  **Date(s) of infection**  11 Jan 2020 (travelled to Guangzhou, Guangdong province, China) | **Setting**  Community (family gathering and contact with other residents in apartment building)  **Location**  Guangzhou, Guangdong province, China  **Earliest exposure to index case**  11 Jan 2020  **Latest follow up**  27 Jan 2020 (or after) | **Identification method**  SARS-CoV-2 RNA (RT-PCR or genetic sequencing)  **Contact tracing**  Limited (extended family and household of another resident of apartment building with whom index case had contact)  **People tested**  Index case’s extended family and other residents in apartment building with whom the index case had contact | **Exposure phase(s) relative to index case’s infection**  Presymptomatic (< 7 days since either index case was infected)  **Precautions/controls used**  Hospital isolation of confirmed cases, no mask worn in apartment building by resident using lift immediately after index case | **Transmission demonstrated**  Uncertain (possibly presymptomatic)  **Attack rate (exposure via index case only)**  Not calculable (possibly at least 2 secondary infections resulting from presymptomatic exposure to index case) | **Clear inclusion criteria:** yes  **Condition measured in standard, reliable way:** yes  **Valid methods used to identify condition:** yes  **Consecutive inclusion:** unclear  **Complete inclusion:** unclear  **Clear reporting of demographics:** yes  **Clear reporting of clinical information:** yes  **Clear reporting of outcomes and follow up:** yes  **Clear reporting of presenting site(s)/clinic(s) demographic information:** not applicable  **Statistical analysis appropriate:** not applicable | Data extracted are for ‘community infection event’, one of several clusters investigated in the article (the other ‘representatives’ of clusters/outbreaks reported in detail involved symptomatic transmission by index cases) |
| Zhang 2020c^[57]^ | **Source of infection**  Meeting people in Hangzhou, Zhejiang province, China who were later confirmed as infected  **Date(s) of infection**  12 and 13 Jan 2020 (discrepancy between article text and table, but extracted dates more plausible given overall context of article) | **Setting**  Community (household and passengers on return flight from Singapore to Hangzhou, Zhejiang province, China)  **Location**  Hangzhou, Zhejiang province, China (after returning from Singapore)  **Earliest exposure to index case**  12 Jan 2020  **Latest follow up**  9 Feb 2020 | **Identification method**  SARS-CoV-2 RNA (RT-PCR)  **Contact tracing**  Limited (index case’s household and close contacts during flight from Singapore)  **People tested**  Index case’s household | **Exposure phase(s) relative to index case’s infection**  Presymptomatic (< 7 days since index case was infected)  Symptomatic  **Precautions/controls used**  Quarantine by airport authorities of index case’s household and close contacts among passengers and crew on return flight from Singapore | **Transmission demonstrated**  Uncertain (possibly pauci-symptomatic)  **Attack rate (exposure via index case only)**  Not calculable (possibly at least 1 secondary infection resulting from presymptomatic or pauci-symptomatic exposure to index case) | **Clear inclusion criteria:** yes  **Condition measured in standard, reliable way:** yes  **Valid methods used to identify condition:** yes  **Consecutive inclusion:** not applicable  **Complete inclusion:** unclear  **Clear reporting of demographics:** yes  **Clear reporting of clinical information:** yes  **Clear reporting of outcomes and follow up:** yes  **Clear reporting of presenting site(s)/clinic(s) demographic information:** not applicable  **Statistical analysis appropriate:** not applicable | Data extracted are for ‘case cluster 1’ in which the index case’s household travelled on holiday to Singapore on 19 Jan 2020, returning to Hangzhou, Zhejiang province, China on 22 Jan 2020; no data relevant to this review could be extracted for ‘case cluster 2’, which relates to an international tour group that visited Singapore and Malaysia (no apparent overlap with ‘cluster A’ tour group of Pung 2020)^[36]^ |
| Wu 2020;^[47]^ Yang 2020;^[50]^ Liu 2020c^[32]^ | **Source of infection**  Uncertain (possibly related to employee visiting wholesale markets on or before 12 and 13 Jan 2020)  **Date(s) of infection**  Uncertain (possibly 12 to 13 Jan 2020 – see under source of infection above) | **Setting**  Community (department store)  **Location**  Tianjin, China  **Earliest exposure to index case**  12 Jan 2020  **Latest follow up**  18 Feb 2020 (Wu 2020)^[47]^, 20 Feb 2020 (Yang 2020)^[50]^ or 22 Feb 2020 (Liu 2020c)^[32]^ | **Identification method**  SARS-CoV-2 RNA (RT-PCR (Liu 2020c))^[32]^  **Contact tracing**  Limited (staff, customers and possibly their close contacts, including family members)  **People tested**  Not reported | **Exposure phase(s) relative to index case’s infection**  Presymptomatic (7 to 10 days since index case was infected)  Symptomatic  **Precautions/controls used**  Disinfection and closure of department store after cluster recognised, surrounding roads disinfected, centralised/home isolation/quarantine of staff and customers | **Transmission demonstrated**  Uncertain (possibly presymptomatic)  **Attack rate (exposure via index case only)**  Not calculable (possibly at least 6 customers with confirmed infection resulting from presymptomatic exposure) | **Clear inclusion criteria:** unclear  **Condition measured in standard, reliable way:** yes  **Valid methods used to identify condition:** yes  **Consecutive inclusion:** not applicable  **Complete inclusion:** unclear  **Clear reporting of demographics:** unclear  **Clear reporting of clinical information:** yes  **Clear reporting of outcomes and follow up:** unclear  **Clear reporting of presenting site(s)/clinic(s) demographic information:** not applicable  **Statistical analysis appropriate:** not applicable | Articles published in Chinese and incomplete translation might account for some lack of clarity in reporting; unless otherwise stated data extracted from Wu 2020,^[47]^ which focused solely on the cluster/outbreak summarised here; Yang 2020^[50]^ reported 377 clusters/outbreaks outside Hubei province, China, including a detailed description of the cluster/outbreak summarised here (another cluster/outbreak reported in detail did not describe the transmission chain and so no data relevant to this review could be extracted); unable to fully translate example of family cluster/outbreak also reported in Liu 2020c,^[32]^ which might refer to presymptomatic transmission |
| Gao 2020a^[14]^ | ***Source of infection***  *Unknown*  ***Date(s) of infection***  *Unknown* | ***Setting***  *Nosocomial (unrecognised infection in emergency department)*  ***Location***  *Guangzhou, Guangdong*  *province, China*  ***Earliest exposure to index case***  *13 Jan 2020*  ***Latest follow up***  *2 Mar 2020* | ***Identification method***  *SARS-CoV-2 RNA (RT-PCR)*  ***Contact tracing***  *Extensive (screening of hospital patients, visitors (family members) and staff)*  ***People tested***  *Patients, including index case, at least some of the visitors, and staff* | ***Exposure phase(s) relative to index case’s infection***  *Asymptomatic (except for shortness of breath associated with underlying health condition and non-COVID-19 abnormalities on CT scan of the chest; days since index case was infected not calculable)*  ***Precautions/controls used***  *Hospital quarantine of index case, other patients and visitors, all of whom wore masks except when eating or drinking; hospital staff wore N95 respirators, isolation gowns and goggles* | ***Transmission demonstrated***  *No (exposure to asymptomatic case with PPE in use)*  ***Attack rate (exposure via index case only)***  *0% (0/455) with PPE in use (95% CI 0% to 0.8%; calculated by HIS team)* | ***Clear inclusion criteria:*** *yes*  ***Condition measured in standard, reliable way:*** *yes*  ***Valid methods used to identify condition:*** *yes*  ***Consecutive inclusion:*** *not applicable*  ***Complete inclusion:*** *yes*  ***Clear reporting of demographics:*** *yes*  ***Clear reporting of clinical information:*** *yes*  ***Clear reporting of outcomes and follow up:*** *yes*  ***Clear reporting of presenting site(s)/clinic(s) demographic information:*** *yes*  ***Statistical analysis appropriate:*** *not applicable* | *None* |
| Li 2020a^[27]^ | **Source of infection**  Travelling via Wuhan, Hubei province, China  **Date(s) of infection**  14 Jan 2020 (travelled to Xuzhou, Jiangsu province, China on 14 Jan 2020) | **Setting**  Community (households and family gatherings) and nosocomial (hospital ward visited by index case)  **Location**  Xuzhou, Jiangsu province, China  **Earliest exposure to index case**  14 Jan 2020  **Latest follow up**  17 Feb 2020 | **Identification method**  SARS-CoV-2 RNA (RT-PCR)  **Contact tracing**  Limited (households, extended family, hospital patients and visitors)  **People tested**  Index case’s extended family, another hospital patient and their extended family) | **Exposure phase(s) relative to index case’s infection**  Presymptomatic (< 7 days since index case was infected)  Symptomatic  **Precautions/controls used**  Not reported | **Transmission demonstrated**  Uncertain (possibly presymptomatic)  **Attack rate (exposure via index case only)**  Not calculable (possibly at least 1 secondary infection resulting from presymptomatic exposure to index case) | **Clear inclusion criteria:** yes  **Condition measured in standard, reliable way:** yes  **Valid methods used to identify condition:** yes  **Consecutive inclusion:** not applicable  **Complete inclusion:** unclear  **Clear reporting of demographics:** yes  **Clear reporting of clinical information:** yes  **Clear reporting of outcomes and follow up:** yes  **Clear reporting of presenting site(s)/clinic(s) demographic information:** unclear  **Statistical analysis appropriate:** not applicable | None |
| Yu 2020^[54]^ | **Source of infection**  Living in Wuhan, Hubei province, China (possibly 2 index cases)  **Date(s) of infection**  Not reported (travelled to Shanghai, China on 15 Jan 2020) | **Setting**  Community (household and family gathering)  **Location**  Shanghai, China  **Earliest exposure to index case**  15 Jan 2020  **Latest follow up**  25 Jan 2020 | **Identification method**  SARS-CoV-2 RNA (RT-PCR)  **Contact tracing**  Limited (extended family only)  **People tested**  Index cases and extended family | **Exposure phase(s) relative to index case’s infection**  Presymptomatic (days since index case was infected not calculable)  **Precautions/controls used**  Hospital isolation of suspected cases | **Transmission demonstrated**  Yes (presymptomatic)  **Attack rate (exposure via index case only)**  Not calculable (at least 1 secondary infection resulting from presymptomatic exposure, direction of transmission uncertain) | **Clear inclusion criteria:** yes  **Condition measured in standard, reliable way:** yes  **Valid methods used to identify condition:** yes  **Consecutive inclusion:** not applicable  **Complete inclusion:** unclear  **Clear reporting of demographics:** yes  **Clear reporting of clinical information:** yes  **Clear reporting of outcomes and follow up:** yes  **Clear reporting of presenting site(s)/clinic(s) demographic information:** not applicable  **Statistical analysis appropriate:** not applicable | None |
| Guan 2020^[17]^ | **Source of infection**  Living in Xiaogan, Hubei province, China  **Date(s) of infection**  Not reported (travelled to Wuhan, Hubei province, China on 15 Jan 2020, and on to Beijing, China on 16 Jan 2020) | **Setting**  Community (household, family gatherings and visiting friends)  **Location**  Wuhan, Hubei province and Beijing, China  **Earliest exposure to index case**  15 Jan 2020  **Latest follow up**  18 Feb 2020 | **Identification method**  SARS-CoV-2 RNA (RT-PCR)  **Contact tracing**  Limited (household, extended family, friends and their extended families)  **People tested**  Index case, extended family, friends and their extended family | **Exposure phase(s) relative to index case’s infection**  Presymptomatic (days since index case was infected not calculable)  Symptomatic  **Precautions/controls used**  Index case ‘wore a mask throughout’, friends wore masks during train journey to Beijing, other contacts mainly home isolation following exposure to index case | **Transmission demonstrated**  Uncertain (possibly presymptomatic)  **Attack rate (exposure via index case only)**  Not calculable (direction of transmission between presumed index case and friends uncertain, incomplete contact tracing and denominator not reported) | **Clear inclusion criteria:** yes  **Condition measured in standard, reliable way:** yes  **Valid methods used to identify condition:** yes  **Consecutive inclusion:** not applicable  **Complete inclusion:** unclear  **Clear reporting of demographics:** yes  **Clear reporting of clinical information:** yes  **Clear reporting of outcomes and follow up:** yes  **Clear reporting of presenting site(s)/clinic(s) demographic information:** not applicable  **Statistical analysis appropriate:** not applicable | None |
| Xiao 2020^[49]^ | **Source of infection**  Visiting gym used by confirmed cases in Bengbu, Anhui province, China  **Date(s) of infection**  15 to 18 Jan 2020 (travelled to Shanghai, China on 19 Jan 2020) | **Setting**  Community (family gatherings, visiting friends)  **Location**  Bengbu, Anhui province and Shanghai, China  **Earliest exposure to index case**  15 Jan 2020 (or later)  **Latest follow up**  21 Feb 2020 | **Identification method**  SARS-CoV-2 RNA (RT-PCR)  **Contact tracing**  Extensive (investigated all close contacts of index case, including family members and healthcare workers)  **People tested**  Unclear (possibly symptomatic cases only) | **Exposure phase(s) relative to index case’s infection**  Presymptomatic (< 7 days since index case was infected)  **Precautions/controls used**  Home/hospital isolation of close contacts of suspected cases, 1 case wore a mask on the way to work | **Transmission demonstrated**  Uncertain (possibly presymptomatic)  **Attack rate (exposure via index case only)**  Not calculable (transmission chain unclear and denominator not reported) | **Clear inclusion criteria:** yes  **Condition measured in standard, reliable way:** yes  **Valid methods used to identify condition:** yes  **Consecutive inclusion:** not applicable  **Complete inclusion:** unclear  **Clear reporting of demographics:** unclear  **Clear reporting of clinical information:** yes  **Clear reporting of outcomes and follow up:** unclear  **Clear reporting of presenting site(s)/clinic(s) demographic information:** not applicable  **Statistical analysis appropriate:** not applicable | Article published in Chinese and incomplete translation might account for some lack of clarity in reporting |
| Song 2020^[43]^ | **Source of infection**  Attending work meeting in Beijing, China (or travelling via Wuhan, Hubei province, China, alternative index case)  **Date(s) of infection**  15 Jan 2020 (or 19 Jan 2020, alternative index case; travelled to Beijing, China on 19 Jan 2020) | **Setting**  Community (family gatherings)  **Location**  Beijing, China  **Earliest exposure to index case**  15 or 19 Jan 2020  **Latest follow up**  6 Mar 2020 | **Identification method**  SARS-CoV-2 RNA (RT-PCR)  **Contact tracing**  Limited (extended family only)  **People tested**  Extended family, including both possible index cases (possibly suspected cases only) | **Exposure phase(s) relative to index case’s infection**  Presymptomatic (< 7 days since either index case was infected)  **Precautions/controls used**  Not reported | **Transmission demonstrated**  Uncertain (presymptomatic)  **Attack rate (exposure via index case only)**  Not calculable (possibly at least 2 secondary infections resulting from presymptomatic exposure, direction of transmission uncertain) | **Clear inclusion criteria:** yes  **Condition measured in standard, reliable way:** yes  **Valid methods used to identify condition:** yes  **Consecutive inclusion:** not applicable  **Complete inclusion:** unclear  **Clear reporting of demographics:** yes  **Clear reporting of clinical information:** yes  **Clear reporting of outcomes and follow up:** yes  **Clear reporting of presenting site(s)/clinic(s) demographic information:** not applicable  **Statistical analysis appropriate:** not applicable | Data extracted are for ‘family B’; data not extracted for 3 other family clusters/outbreaks involving symptomatic transmission |
| *Scott 2020^[42]^* | ***Source of infection***  *Visiting family in Wuhan and other locations in Hubei province, China*  ***Date(s) of infection***  *7 to 19 Jan 2020 (travelled to Maricopa County, Arizona, USA 19 Jan 2020)* | ***Setting***  *Community (household and rideshare partners) and nosocomial (through contact with healthcare setting)*  ***Location***  *Maricopa County, Arizona, USA*  ***Earliest exposure to index case***  *Before 19 Jan 2020*  ***Latest follow up***  *5 Mar 2020* | ***Identification method***  *SARS-CoV-2 RNA (RT-PCR)*  ***Contact tracing***  *Limited (people exposed to index case from 1 day before clinical samples obtained, including household, rideshare partners and healthcare workers)*  ***People tested***  *Index case, other suspected (symptomatic) cases and high-risk contacts (prolonged contact (>= 10 minutes) at < 2 metres)* | ***Exposure phase(s) relative to index case’s infection***  *Asymptomatic (except for ‘previously existing mild non-productive cough’; 11 to 14 days since index case was infected)*  ***Precautions/controls used***  *Index case wore a mask while travelling to clinic and during clinic visit, and while in same room as housemates after returning home; home quarantine with separate accommodation for housemates; home quarantine for healthcare workers with high-risk exposure (close contact or not using recommended PPE)* | ***Transmission demonstrated***  *No (asymptomatic, except for pre-existing cough, some PPE use)*  ***Attack rate (exposure via index case only)***  *0% (0/10) among high-risk contacts; possibility of PPE being used (95% CI 0% to 31%; calculated by HIS team)* | ***Clear inclusion criteria:*** *yes*  ***Condition measured in standard, reliable way:*** *yes*  ***Valid methods used to identify condition:*** *yes*  ***Consecutive inclusion:*** *not applicable*  ***Complete inclusion:*** *unclear*  ***Clear reporting of demographics:*** *unclear*  ***Clear reporting of clinical information:*** *yes*  ***Clear reporting of outcomes and follow up:*** *yes*  ***Clear reporting of presenting site(s)/clinic(s) demographic information:*** *not applicable*  ***Statistical analysis appropriate:*** *not applicable* | *None* |
| Qian 2020^[37]^ | **Source of infection**  Visiting temple in Zhejiang province, China (2 index cases with common exposure)  **Date(s) of infection**  19 Jan 2020 | **Setting**  Community (household and family gatherings)  **Location**  Zhejiang province, China  **Earliest exposure to index case**  19 Jan 2020  **Latest follow up**  11 Feb 2020 | **Identification method**  SARS-CoV-2 RNA (RT-PCR)  **Contact tracing**  Limited (extended family only)  **People tested**  Extended family, including index cases | **Exposure phase(s) relative to index case’s infection**  Presymptomatic (< 7 days since index case was infected) or asymptomatic (no testing/investigations other than RT-PCR reported; < 7 days since index case was infected)  **Precautions/controls used**  Hospital quarantine for contacts | **Transmission demonstrated**  Uncertain (possibly presymptomatic, assuming first index case infected first; second index patient asymptomatic and tested positive after first index case)  **Attack rate (exposure via index case only)**  *75% (3/4), or 80% (4/5) if second index case counted as exposed (95% CI 19% to 99% or 28% to 99%, respectively; calculated by HIS team)* | **Clear inclusion criteria:** yes  **Condition measured in standard, reliable way:** yes  **Valid methods used to identify condition:** yes  **Consecutive inclusion:** not applicable  **Complete inclusion:** unclear  **Clear reporting of demographics:** yes  **Clear reporting of clinical information:** yes  **Clear reporting of outcomes and follow up:** yes  **Clear reporting of presenting site(s)/clinic(s) demographic information:** not applicable  **Statistical analysis appropriate:** not applicable | None |
| Huang 2020a^[23]^ | **Source of infection**  Working in Wuhan, Hubei province, China  **Date(s) of infection**  7 to 19 Jan 2020 | **Setting**  Community (household, family gathering and meetings with friends in restaurants and karaoke room)  **Location**  Hefei (including Feidong), Anhui province, China  **Earliest exposure to index case**  19 Jan 2020  **Latest follow up**  20 Feb 2020 | **Identification method**  SARS-CoV-2 RNA (RT-PCR; at least 2 positive test results at least 24 hours apart required for confirmation of infection)  **Contact tracing**  Extensive (close contacts of index case and their contacts, including households, relatives, classmates, friends,  and healthcare workers where relevant)  **People tested**  Index case and close contacts | **Exposure phase(s) relative to index case’s infection**  Presymptomatic (days since index case was infected not calculable, but <= 12 days since index case was infected)  **Precautions/controls used**  Hospital/home quarantine of close contacts without symptoms, wearing masks and with emphasis on hand hygiene once symptoms developed; index case visited relatives on 19 Jan 2020 without wearing a mask, but wore a mask while attending outpatient clinic on 22 Jan 2020; healthcare workers used PPE (contact, droplet, and airborne precautions with eye protection) when caring for index case after admission/isolation | **Transmission demonstrated**  Yes (presymptomatic)  **Attack rate (exposure via index case only)**  40% (7/22) among household, extended family and friends of index case with close contact during presymptomatic phase; excludes contacts who reported transient subjective symptoms, but tested negative using RT-PCR (95% CI 14% to 55%; calculated by HIS team) | **Clear inclusion criteria:** yes  **Condition measured in standard, reliable way:** yes  **Valid methods used to identify condition:** yes  **Consecutive inclusion:** not applicable  **Complete inclusion:** yes  **Clear reporting of demographics:** yes  **Clear reporting of clinical information:** yes  **Clear reporting of outcomes and follow up:** yes  **Clear reporting of presenting site(s)/clinic(s) demographic information:** not applicable  **Statistical analysis appropriate:** not applicable | None |
| Yong 2020;^[53]^ Pung 2020;^[36]^ Wei 2020^[46]^ | **Source of infection**  Living in Wuhan, Hubei province, China (possibly 2 index cases)  **Date(s) of infection**  Not reported (travelled to Singapore on 19 Jan 2020 (Pung 2020^[36]^ and Wei 2020))^[46]^ | **Setting**  Community (church attendance)  **Location**  Singapore  **Earliest exposure to index case**  19 Jan 2020  **Latest follow up**  26 Feb 2020 (Yong 2020)^[53]^ or 7 Mar 2020 (Pung 2020)^[36]^ or 16 Mar 2020 (Wei 2020)^[46]^ | **Identification method**  SARS-CoV-2 RNA (RT-PCR) or serological testing (ELISA and VNT)  **Contact tracing**  Comprehensive (all people with prolonged contact at < 2 metres from 14 days before symptoms started until isolation of confirmed cases)  **People tested**  Suspected (symptomatic) cases, including all patients admitted to hospital with pneumonia (ELISA and VNT testing for 1 case who had recovered from infection) | **Exposure phase(s) relative to index case’s infection**  Presymptomatic (days since index case was infected not calculable)  **Precautions/controls used**  Hospital isolation of confirmed cases and centralised/home quarantine of close contacts, supported by active case finding (Pung 2020)^[36]^ | **Transmission demonstrated**  Yes (presymptomatic)  **Attack rate (exposure via index case only)**  Not calculable (at least 3 secondary infections resulting from presymptomatic exposure to index case) | **Clear inclusion criteria:** yes  **Condition measured in standard, reliable way:** yes  **Valid methods used to identify condition:** yes  **Consecutive inclusion:** not applicable  **Complete inclusion:** yes  **Clear reporting of demographics:** yes  **Clear reporting of clinical information:** yes  **Clear reporting of outcomes and follow up:** yes  **Clear reporting of presenting site(s)/clinic(s) demographic information:** not applicable  **Statistical analysis appropriate:** not applicable | Unless otherwise stated data extracted from Yong 2020,^[53]^ which focused mainly on linking 3 clusters, including ‘church A’ for which data extracted here (data not extracted for ‘family gathering’ or ‘church B’ because these involved symptomatic transmission); ‘cluster C’ of Pung 2020^[36]^ and ‘cluster A’ of Wei 2020^[46]^ assumed to be the same as ‘church A’ of Yong 2020;^[53]^; Pung 2020^[36]^ focused on first 3 clusters identified in Singapore (‘cluster A’ – tourist group from China and shopworkers – excluded because members of tour group and local shopworker reported as having symptoms concurrently; ‘cluster B’ – company conference summarised separately); primary aim of Wei 2020^[46]^ was to summarise evidence from clusters most likely to demonstrate presymptomatic transmission based on systematic investigation of all confirmed cases in Singapore (7 clusters in total; ‘cluster B’ to ‘cluster G’ summarised separately) |
| *Zhang 2020a^[55]^* | ***Source of infection***  *Temporarily in Wuhan, Hubei province, China (including visiting relative)*  ***Date(s) of infection***  *Not reported (travelled to Beijing, China on 19 Jan 2020)* | ***Setting***  *Community (household and family gathering)*  ***Location***  *Beijing, China*  ***Earliest exposure to index case***  *19 Jan 2020*  ***Latest follow up***  *29 Feb 2020* | ***Identification method***  *SARS-CoV-2 RNA (RT-PCR)*  ***Contact tracing***  *Limited (extended family only)*  ***People tested***  *Extended family, including index case* | ***Exposure phase(s) relative to index case’s infection***  *Asymptomatic (but with ground-glass appearance on CT scan of the chest; days since index case was infected not calculable)*  ***Precautions/controls used***  *Hospital isolation of confirmed cases* | ***Transmission demonstrated***  *Uncertain (possibly asymptomatic, but with ground-glass appearance on CT scan of the chest)*  ***Attack rate (exposure via index case only)***  *Not calculable (possibly at least 3 secondary infections resulting from asymptomatic exposure to index case)* | ***Clear inclusion criteria:*** *yes*  ***Condition measured in standard, reliable way:*** *yes*  ***Valid methods used to identify condition:*** *yes*  ***Consecutive inclusion:*** *not applicable*  ***Complete inclusion:*** *unclear*  ***Clear reporting of demographics:*** *yes*  ***Clear reporting of clinical information:*** *yes*  ***Clear reporting of outcomes and follow up:*** *yes*  ***Clear reporting of presenting site(s)/clinic(s) demographic information:*** *not applicable*  ***Statistical analysis appropriate:*** *not applicable* | *None* |
| Gao 2020b^[15]^ | **Source of infection**  Attending work meeting involving residents of Wuhan, Hubei province, China (meeting location not reported, but index case stayed in same hotel as Wuhan residents)  **Date(s) of infection**  15 and 16 Jan 2020 (travelled on to Japan on 19 Jan 2020 and returned to Wuxi, Jiangsu province, China on 24 Jan 2020) | **Setting**  Community (fellow travellers in tour group visiting Japan and their relatives/friends)  **Location**  Japan and Wuxi, Jiangsu province, China  **Earliest exposure to index case**  19 Jan 2020  **Latest follow up**  11 Mar 2020 | **Identification method**  SARS-CoV-2 RNA (RT-PCR or genetic sequencing)  **Contact tracing**  Limited (contacts of first symptomatic case, including tour group visiting Japan and their contacts)  **People tested**  First symptomatic case and at least some of their contacts, including index case | **Exposure phase(s) relative to index case’s infection**  Presymptomatic (< 7 days since index case was infected)  **Precautions/controls used**  Quarantine of close contacts | **Transmission demonstrated**  Uncertain (possibly presymptomatic)  **Attack rate (exposure via index case only)**  Not calculable (possibly at least 1 secondary infection resulting from presymptomatic exposure to index case) | **Clear inclusion criteria:** yes  **Condition measured in standard, reliable way:** unclear  **Valid methods used to identify condition:** yes  **Consecutive inclusion:** not applicable  **Complete inclusion:** unclear  **Clear reporting of demographics:** yes  **Clear reporting of clinical information:** yes  **Clear reporting of outcomes and follow up:** yes  **Clear reporting of presenting site(s)/clinic(s) demographic information:** not applicable  **Statistical analysis appropriate:** not applicable | None |
| Zhao 2020^[58]^ | **Source of infection**  Working in Jingzhou, Hubei province, China  **Date(s) of infection**  Not reported (travelled to Chongqing, China on 20 Jan 2020) | **Setting**  Community (household, relatives, friends, workers in restaurant and Mahjong hall)  **Location**  Chongqing, China  **Earliest exposure to index case**  20 Jan 2020  **Latest follow up**  9 Feb 2020 (or after) | **Identification method**  SARS-CoV-2 RNA (PCR, no further details reported)  **Contact tracing**  Extensive (extended family, fellow students, work colleagues, etc)  **People tested**  Uncertain (index case and at least some contacts) | **Exposure phase(s) relative to index case’s infection**  Uncertain, but probably presymptomatic (days since index case was infected not calculable)  **Precautions/controls used**  None | **Transmission demonstrated**  Uncertain (possibly presymptomatic)  **Attack rate (exposure via index case only)**  Not calculable (across 3 generations of infection investigated, 18% (13/74) people exposed only ‘during the incubation period’ became infected) | **Clear inclusion criteria:** yes  **Condition measured in standard, reliable way:** unclear  **Valid methods used to identify condition:** yes  **Consecutive inclusion:** not applicable  **Complete inclusion:** unclear  **Clear reporting of demographics:** unclear  **Clear reporting of clinical information:** not applicable  **Clear reporting of outcomes and follow up:** yes  **Clear reporting of presenting site(s)/clinic(s) demographic information:** not applicable  **Statistical analysis appropriate:** not applicable | Article published in Chinese and incomplete translation might account for some lack of clarity in reporting |
| Pung 2020^[36]^ | **Source of infection**  Living in Wuhan, Hubei province, China or other location in mainland China (several possible index cases)  **Date(s) of infection**  Not reported (travelled to Singapore on 20 Jan 2020 or before) | **Setting**  Community (work meeting/conference involving employees living in 19 countries and their households)  **Location**  Singapore  **Earliest exposure to index case**  20 Jan 2020  **Latest follow up**  7 Mar 2020 | **Identification method**  SARS-CoV-2 RNA (RT-PCR)  **Contact tracing**  Comprehensive (all people with contact from 14 days before symptoms started until isolation of confirmed cases, including hotel workers in the conference venue); open source reports used for contacts outside Singapore; active case finding with support from the Malaysian  International Health Regulation focal point (first symptomatic case was identified in Malaysia after returning from the conference)  **People tested**  Conference attendees remaining in Singapore | **Exposure phase(s) relative to index case’s infection**  Unknown (days since index case was infected not calculable, index case not clearly identified, but assumed to be conference attendee from China)  **Precautions/controls used**  Hospital isolation of suspected/ confirmed cases, centralised/home quarantine of close contacts (those who had prolonged contact with a confirmed case at < 2 metres), telephone surveillance of other contacts | **Transmission demonstrated**  Uncertain (possibly presymptomatic or asymptomatic, but insufficient information about index case to infer symptom status)  **Attack rate (exposure via index case only)**  Not calculable (at least 7 secondary infections resulting from exposure during the conference, transmission chain uncertain) | **Clear inclusion criteria:** yes  **Condition measured in standard, reliable way:** yes  **Valid methods used to identify condition:** yes  **Consecutive inclusion:** not applicable  **Complete inclusion:** yes  **Clear reporting of demographics:** unclear  **Clear reporting of clinical information:** yes  **Clear reporting of outcomes and follow up:** yes  **Clear reporting of presenting site(s)/clinic(s) demographic information:** not applicable  **Statistical analysis appropriate:** not applicable | Data extracted are for transmission chain associated with ‘cluster B’ – company conference; article focused on first 3 clusters identified in Singapore (‘cluster A’ – tourist group from China and shopworkers – excluded because members of tour group and local shopworker reported as having symptoms concurrently; ‘cluster C’ – church – summarised alongside Yong 2020^[53]^ and Wei 2020)^[46]^ |
| Rothe 2020;^[41]^ Kupferschmidt 2020;^[26]^ Bohmer 2020^[7]^ | **Source of infection**  Uncertain (being visited in Shanghai, China by relatives living in Wuhan, Hubei province, China (Bohmer 2020)^[7]^ or sitting near coughing passenger during flight to Munich, Bavaria, Germany)  **Date(s) of infection**  16 Jan 2020 (visited by relatives; Bohmer 2020)^[7]^ or 19 Jan 2020 (travelled to Munich, Bavaria, Germany) | **Setting**  Community (work)  **Location**  Munich, Bavaria, Germany  **Earliest exposure to index case**  20 Jan 2020  **Latest follow up**  5 Feb 2020 (Rothe 2020)^[41]^ or 2 May 2020 (Bohmer 2020)^[7]^ | **Identification method**  SARS-CoV-2 RNA (RT-PCR)  **Contact tracing**  Extensive (all people with confirmed infection linked to index case and their contacts; Bohmer 2020)^[7]^  **People tested**  People who had high-risk contact with a confirmed case (cumulative face-to-face contact > 15 minutes, direct contact with secretions or body fluids of confirmed case, or healthcare workers who worked within 2 metres of a confirmed case without PPE) and other symptomatic contacts (Bohmer 2020)^[7]^ | **Exposure phase(s) relative to index case’s infection**  Presymptomatic (< 7 days since index case was infected) or pauci-symptomatic (reported originally as asymptomatic; index case had taken medicine containing paracetamol)  **Precautions/controls used**  Hospital isolation of cases confirmed in Germany; home quarantine for high-risk contacts and company site closed and disinfected after cases confirmed (Bohmer 2020)^[7]^ | **Transmission demonstrated**  Uncertain (presymptomatic or pauci-symptomatic)  **Attack rate (exposure via index case only)**  Not calculable (at least 1 secondary infection resulting from presymptomatic/pauci-symptomatic exposure to index case) | **Clear inclusion criteria:** yes  **Condition measured in standard, reliable way:** yes  **Valid methods used to identify condition:** yes  **Consecutive inclusion:** not applicable  **Complete inclusion:** unclear  **Clear reporting of demographics:** yes  **Clear reporting of clinical information:** yes  **Clear reporting of outcomes and follow up:** yes  **Clear reporting of presenting site(s)/clinic(s) demographic information:** not applicable  **Statistical analysis appropriate:** /not applicable | Unless otherwise stated data extracted from Rothe 2020;^[41]^ Kupferschmidt 2020^[26]^ and Bohmer 2020^[7]^ reported independent commentary and investigation, respectively, on the same cluster/outbreak |
| Huang 2020b^[24]^ | **Source of infection**  Travelling via Wuhan, Hubei province, China  **Date(s) of infection**  21 Jan 2020 (travelled to Nanjing, Jiangsu province, China) | **Setting**  Community (household and family gatherings)  **Location**  Nanjing, Jiangsu province, China  **Earliest exposure to index case**  21 Jan 2020  **Latest follow up**  2 Feb 2020 | **Identification method**  SARS-CoV-2 RNA (PCR, no further details reported)  **Contact tracing**  Limited (extended family only)  **People tested**  Extended family, including index case | **Exposure phase(s) relative to index case’s infection**  Presymptomatic (days since index case was infected not calculable)  **Precautions/controls used**  Not reported | **Transmission demonstrated**  Uncertain (possibly presymptomatic)  **Attack rate (exposure via index case only)**  Not calculable (possibly at least 4 secondary infections resulting from presymptomatic exposure to index case) | **Clear inclusion criteria:** yes  **Condition measured in standard, reliable way:** yes  **Valid methods used to identify condition:** yes  **Consecutive inclusion:** not applicable  **Complete inclusion:** unclear  **Clear reporting of demographics:** unclear  **Clear reporting of clinical information:** yes  **Clear reporting of outcomes and follow up:** yes  **Clear reporting of presenting site(s)/clinic(s) demographic information:** not applicable  **Statistical analysis appropriate:** not applicable | None |
| Bae 2020^[4]^ | **Source of infection**  Living in Wuhan, Hubei province, China  **Date(s) of infection**  Not reported (travelled via Yangzhou, Jiangsu province, China to Jeju Island, South Korea on 21 Jan 2020) | **Setting**  Community (tourism)  **Location**  Jeju Island, South Korea  **Earliest exposure to index case**  21 Jan 2020  **Latest follow up**  8 Feb 2020 | **Identification method**  Clinical (symptoms suggesting infection)  **Contact tracing**  Extensive (based on CCTV in hotel and travel route through Jeju Island, South Korea)  **People tested**  None (quarantine included monitoring for symptoms, but none were reported) | **Exposure phase(s) relative to index case’s infection**  Presymptomatic (days since index case was infected not calculable)  **Precautions/controls used**  Quarantine of close contacts (duration > 10 seconds) on Jeju Island, South Korea | **Transmission demonstrated**  No (presymptomatic)  **Attack rate (exposure via index case only)**  0% (0/11) among close contacts on Jeju Island, South Korea (95% CI 0% to 28%; calculated by HIS team) | **Clear inclusion criteria:** yes  **Condition measured in standard, reliable way:** unclear  **Valid methods used to identify condition:** unclear  **Consecutive inclusion:** not applicable  **Complete inclusion:** yes  **Clear reporting of demographics:** unclear  **Clear reporting of clinical information:** yes  **Clear reporting of outcomes and follow up:** yes  **Clear reporting of presenting site(s)/clinic(s) demographic information:** not applicable  **Statistical analysis appropriate:** not applicable | None |
| Liu 2020b^[31]^ | **Source of infection**  Temporarily living in Wuhan, Hubei province, China  **Date(s) of infection**  21 Oct 2019 to 20 Jan 2020 (travelled to Taiwan on 20 Jan 2020) | **Setting**  Community (household)  **Location**  Taiwan  **Earliest exposure to index case**  21 Jan 2020  **Latest follow up**  11 Feb 2020 | **Identification method**  SARS-CoV-2 RNA (RT-PCR)  **Contact tracing**  Limited (household only)  **People tested**  Index case’s household | **Exposure phase(s) relative to index case’s infection**  Presymptomatic (days since index case was infected not calculable)  **Precautions/controls used**  Not reported | **Transmission demonstrated**  Yes (presymptomatic)  **Attack rate (exposure via index case only)**  100% (1/1) among household members (95% CI 3% to 100%; calculated by HIS team) | **Clear inclusion criteria:** yes  **Condition measured in standard, reliable way:** yes  **Valid methods used to identify condition:** yes  **Consecutive inclusion:** not applicable  **Complete inclusion:** unclear  **Clear reporting of demographics:** yes  **Clear reporting of clinical information:** yes  **Clear reporting of outcomes and follow up:** yes  **Clear reporting of presenting site(s)/clinic(s) demographic information:** not applicable  **Statistical analysis appropriate:** not applicable | None |
| *Lu 2020b^[34]^* | ***Source of infection***  *Not reported (index case assumed to be relative resident in Xiangyang, Hubei province, China)*  ***Date(s) of infection***  *Not reported (travelled to Guangdong province, China on 21 Jan 2020)* | ***Setting***  *Community (family gathering)*  ***Location***  *Guangdong province, China*  ***Earliest exposure to index case***  *21 Jan 2020*  ***Latest follow up***  *13 Feb 2020* | ***Identification method***  *SARS-CoV-2 RNA (nucleic acid, no further details reported) or radiological (ground-glass appearance on CT scan of the chest, first confirmed case only)*  ***Contact tracing***  *Unclear (‘close contacts’ of first confirmed case, including extended family)*  ***People tested***  *Unclear (first confirmed case and at least two contacts, including index case)* | ***Exposure phase(s) relative to index case’s infection***  *Asymptomatic (but with ground-glass appearance on CT scan of the chest; days since index case was infected not calculable)*  ***Precautions/controls used***  *Hospital investigation for contacts of first confirmed case* | ***Transmission demonstrated***  *Uncertain (possibly asymptomatic, but with ground-glass appearance on CT scan of the chest)*  ***Attack rate (exposure via index case only)***  *Not calculable (possibly at least 1 secondary infection resulting from asymptomatic exposure to index case)* | ***Clear inclusion criteria:*** *unclear*  ***Condition measured in standard, reliable way:*** *unclear*  ***Valid methods used to identify condition:*** *unclear*  ***Consecutive inclusion:*** *not applicable*  ***Complete inclusion:*** *unclear*  ***Clear reporting of demographics:*** *unclear*  ***Clear reporting of clinical information:*** *yes*  ***Clear reporting of outcomes and follow up:*** *yes*  ***Clear reporting of presenting site(s)/clinic(s) demographic information:*** *not applicable*  ***Statistical analysis appropriate:*** *not applicable* | *None* |
| *Hu 2020^[22]^* | ***Source of infection***  *Visiting Huanggang, Hubei province, China*  ***Date(s) of infection***  *19 to 20 Jan 2020 (returned to Nanjing, Jiangsu province, China on 21 Jan 2020)* | ***Setting***  *Community (household)*  ***Location***  *Nanjing, Jiangsu province, China*  ***Earliest exposure to index case***  *21 Jan 2020*  ***Latest follow up***  *18 Feb 2020* | ***Identification method***  *SARS-CoV-2 RNA (RT-PCR)*  ***Contact tracing***  *Extensive (cohabiting family members of confirmed/suspected cases and other close contacts for symptomatic cases as part of larger study)*  ***People tested***  *Index case and other members of their household* | ***Exposure phase(s) relative to index case’s infection***  *Asymptomatic (but with typical signs of viral infection on CT scan of the chest; < 7 days since index case was infected)*  ***Precautions/controls used***  *Hospital admission for all asymptomatic carriers* | ***Transmission demonstrated***  *Yes (asymptomatic, but with typical signs of viral infection on CT scan of the chest)*  ***Attack rate (exposure via index case only)***  *100% (3/3) assuming all members of index case’s household are reported (95% CI 29% to 100%; calculated by HIS team)* | ***Clear inclusion criteria:*** *yes*  ***Condition measured in standard, reliable way:*** *yes*  ***Valid methods used to identify condition:*** *yes*  ***Consecutive inclusion:*** *yes*  ***Complete inclusion:*** *yes*  ***Clear reporting of demographics:*** *yes*  ***Clear reporting of clinical information:*** *yes*  ***Clear reporting of outcomes and follow up:*** *yes*  ***Clear reporting of presenting site(s)/clinic(s) demographic information:*** *yes*  ***Statistical analysis appropriate:*** *not applicable* | *Data extracted are for detailed investigation of transmission chain associated with ‘case 13’; part of larger study focused on clinical characteristics of asymptomatic/presymptomatic infections; transmission chains for other cases not reported* |
| *Qiu 2020a^[38]^* | ***Source of infection***  *Various – mainly living in or visiting Wuhan, Hubei province, China or having contact with another person who had been in Wuhan (case ‘C’4’ in ‘cluster 19’ had contact with colleague who had been in Wuhan)*  ***Date(s) of infection***  *Not reported* | ***Setting***  *Community (households, family gatherings, meeting friends, work, shared transport, etc)*  ***Location***  *Huaihua and Shaoyang, Hunan province, China*  ***Earliest exposure to index case***  *Before 22 Jan 2020*  ***Latest follow up***  *12 Feb 2020* | ***Identification method***  *SARS-CoV-2 RNA (RT-PCR)*  ***Contact tracing***  *Extensive (contact history investigated for all confirmed cases)*  ***People tested***  *Not reported (but includes case ‘C’4’ and subsequent cases in ‘cluster 19’; retrospective study of confirmed cases to explore their contact history and associated transmission chains)* | ***Exposure phase(s) relative to index case’s infection***  *Asymptomatic (‘no symptoms*  *with a positive result of PCR test’; days since index case was infected not calculable)*  ***Precautions/controls used***  *‘Level one emergency response’ implemented in Hunan province, China on 24 Jan 2020; PPE used in hospitals (no further details reported)* | ***Transmission demonstrated***  *Uncertain (asymptomatic, but uncertain whether e.g. CT scan of the chest performed)*  ***Attack rate (exposure via index case only)***  *Not calculable (possibly at least 1 secondary infection resulting from asymptomatic exposure to index case ‘C’4’)* | ***Clear inclusion criteria:*** *yes*  ***Condition measured in standard, reliable way:*** *unclear*  ***Valid methods used to identify condition:*** *yes*  ***Consecutive inclusion:*** *yes*  ***Complete inclusion:*** *yes*  ***Clear reporting of demographics:*** *unclear*  ***Clear reporting of clinical information:*** *unclear*  ***Clear reporting of outcomes and follow up:*** *unclear*  ***Clear reporting of presenting site(s)/clinic(s) demographic information:*** *unclear*  ***Statistical analysis appropriate:*** *not applicable* | *Data extracted are for detailed investigation of transmission chain associated with ‘cluster 19’; part of larger study focused on transmission and clinical characteristics of infection (data not extracted for other clusters involving symptomatic transmission or possible asymptomatic transmission, but without testing for SARS-CoV-2 RNA)* |
| Qiu 2020b^[39]^ | **Source of infection**  Working in Wuhan, Hubei province, China (possibly 2 index cases with common exposure)  **Date(s) of infection**  22 Nov 2019 to 22 Jan 2020 (travelled to Zhengzhou, Henan province, China on 22 Jan 2020) | **Setting**  Community (household and family gatherings)  **Location**  Zhengzhou, Henan province, China  **Earliest exposure to index case**  22 Jan 2020  **Latest follow up**  11 Feb 2020 | **Identification method**  SARS-CoV-2 RNA (RT-PCR)  **Contact tracing**  Limited (extended family)  **People tested**  Suspected cases | **Exposure phase(s) relative to index case’s infection**  Presymptomatic (days since index case was infected not calculable)  Symptomatic  **Precautions/controls used**  Hospital isolation of suspected cases | **Transmission demonstrated**  Uncertain (possibly presymptomatic)  **Attack rate (exposure via index case only)**  Not calculable (possibly at least 1 secondary infection resulting from presymptomatic exposure to index case) | **Clear inclusion criteria:** unclear  **Condition measured in standard, reliable way:** unclear  **Valid methods used to identify condition:** yes  **Consecutive inclusion:** not applicable  **Complete inclusion:** unclear  **Clear reporting of demographics:** yes  **Clear reporting of clinical information:** yes  **Clear reporting of outcomes and follow up:** yes  **Clear reporting of presenting site(s)/clinic(s) demographic information:** not applicable  **Statistical analysis appropriate:** not applicable | Article published in Chinese and incomplete translation might account for some lack of clarity in reporting |
| Ye 2020^[51]^ | **Source of infection**  Living in Wuhan, Hubei province, China (possibly 3 index cases)  **Date(s) of infection**  Not reported (travelled to Luzhou, Sichuan province, China on 22 Jan 2020) | **Setting**  Community (household and family gatherings)  **Location**  Luzhou, Sichuan province, China  **Earliest exposure to index case**  22 Jan 2020  **Latest follow up**  15 Feb 2020 | **Identification method**  SARS-CoV-2 RNA (RT-PCR)  **Contact tracing**  Extensive (44 contacts of first confirmed case admitted to hospital for physical examination)  **People tested**  Uncertain (but included index case and extended family) | **Exposure phase(s) relative to index case’s infection**  Presymptomatic (days since index case was infected not calculable)  **Precautions/controls used**  Hospital isolation of confirmed cases | **Transmission demonstrated**  Yes (presymptomatic)  **Attack rate (exposure via index case only)**  Not calculable (at least 2 secondary infections resulting from presymptomatic exposure to index case) | **Clear inclusion criteria:** yes  **Condition measured in standard, reliable way:** unclear  **Valid methods used to identify condition:** yes  **Consecutive inclusion:** not applicable  **Complete inclusion:** unclear  **Clear reporting of demographics:** yes  **Clear reporting of clinical information:** yes  **Clear reporting of outcomes and follow up:** yes  **Clear reporting of presenting site(s)/clinic(s) demographic information:** not applicable  **Statistical analysis appropriate:** not applicable | None |
| Lu 2020a^[33]^ | **Source of infection**  Living in Wuhan, Hubei province, China (possibly 4 index cases with at least some common exposure)  **Date(s) of infection**  Not reported (travelled to Guangzhou, Guangdong province, China on 23 Jan 2020) | **Setting**  Community (household and restaurant)  **Location**  Guangzhou, Guangdong province, China  **Earliest exposure to index case**  24 Jan 2020 (excluding index case’s household)  **Latest follow up**  10 Feb 2020 | **Identification method**  SARS-CoV-2 RNA (RT-PCR)  **Contact tracing**  Extensive (customers and staff present in restaurant at lunchtime on 24 Jan 2020)  **People tested**  Index case and restaurant contacts | **Exposure phase(s) relative to index case’s infection**  Presymptomatic (days since index case was infected not calculable)  **Precautions/controls used**  Quarantine (location not reported) of customers who ate lunch in the restaurant on 24 Jan 2020 | **Transmission demonstrated**  Yes (presymptomatic)  **Attack rate (exposure via index case only)**  Not calculable (at least 2 secondary infections resulting from presymptomatic exposure to the index case) | **Clear inclusion criteria:** yes  **Condition measured in standard, reliable way:** yes  **Valid methods used to identify condition:** yes  **Consecutive inclusion:** not applicable  **Complete inclusion:** yes  **Clear reporting of demographics:** unclear  **Clear reporting of clinical information:** yes  **Clear reporting of outcomes and follow up:** yes  **Clear reporting of presenting site(s)/clinic(s) demographic information:** not applicable  **Statistical analysis appropriate:** not applicable | None |
| Li 2020b^[28]^ | **Source of infection**  Visiting relatives in Wuxi, Jiangsu  province, China  **Date(s) of infection**  26 to 28 Jan 2020 (travelled on to Zhoushan, Zhejiang province, China on 31 Jan 2020) | **Setting**  Community (family gatherings)  **Location**  Zhoushan, Zhejiang province, China  **Earliest exposure to index case**  31 Jan 2020  **Latest follow up**  11 Feb 2020 | **Identification method**  SARS-CoV-2 RNA (RT-PCR)  **Contact tracing**  Limited (extended family only)  **People tested**  Extended family, including index case | **Exposure phase(s) relative to index case’s infection**  Presymptomatic (< 7 days since index case was infected)  **Precautions/controls used**  Home quarantine of index case from 29 Jan 2020 (after relatives in Wuxi, Jiangsu province, China identified as suspected cases) not adhered to; hospital isolation from 5 Feb 2020 (after relatives in Wuxi confirmed as infected) | **Transmission demonstrated**  Uncertain (possibly presymptomatic)  **Attack rate (exposure via index case only)**  Not calculable (possibly at least 1 secondary infection resulting from presymptomatic exposure to index case) | **Clear inclusion criteria:** yes  **Condition measured in standard, reliable way:** yes  **Valid methods used to identify condition:** yes  **Consecutive inclusion:** not applicable  **Complete inclusion:** unclear  **Clear reporting of demographics:** yes  **Clear reporting of clinical information:** yes  **Clear reporting of outcomes and follow up:** yes  **Clear reporting of presenting site(s)/clinic(s) demographic information:** not applicable  **Statistical analysis appropriate:** not applicable | None |
| Hijnen 2020^[21]^ | **Source of infection**  Working in Milan, Lombardy, Italy (dermatology outpatient clinic)  **Date(s) of infection**  18 Feb 2020 (travelled to Munich, Bavaria, Germany on or before 20 Feb 2020) | **Setting**  Community (work meeting and taxi journey)  **Location**  Munich, Bavaria Germany  **Earliest exposure to index case**  20 Feb 2020  **Latest follow up**  20 Apr 2020 | **Identification method**  SARS-CoV-2 RNA (RT-PCR) or serological testing (ELISA)  **Contact tracing**  Limited (meeting attendees, their households, work colleagues, etc)  **People tested**  Index case and other meeting attendees (except 1 with no signs of infection; ELISA testing for 1 attendee for whom SARS-CoV-2 RNA testing not available) | **Exposure phase(s) relative to index case’s infection**  Presymptomatic (< 7 days since index case was infected)  **Precautions/controls used**  Nobody wore a mask during the meeting; hospital/hotel/home isolation of meeting attendees after index case’s infection confirmed | **Transmission demonstrated**  Yes (presymptomatic)  **Attack rate (exposure via index case only)**  At least 85% (11/13) among meeting attendees (95% CI 55% to 98%; calculated by HIS team) | **Clear inclusion criteria:** yes  **Condition measured in standard, reliable way:** yes  **Valid methods used to identify condition:** yes  **Consecutive inclusion:** not applicable  **Complete inclusion:** yes  **Clear reporting of demographics:** yes  **Clear reporting of clinical information:** yes  **Clear reporting of outcomes and follow up:** yes  **Clear reporting of presenting site(s)/clinic(s) demographic information:** not applicable  **Statistical analysis appropriate:** not applicable | None |
| Wei 2020^[46]^ | **Source of infection**  Having dinner with confirmed case, Singapore  **Date(s) of infection**  15 Feb 2020 (attended singing class on 24 Feb 2020) | **Setting**  Community (singing class)  **Location**  Singapore  **Earliest exposure to index case**  24 Feb 2020  **Latest follow up**  29 Feb 2020 | **Identification method**  SARS-CoV-2 RNA (PCR, no further details reported)  **Contact tracing**  Extensive (active case finding and monitoring from onset of symptoms)  **People tested**  Suspected (symptomatic) cases; asymptomatic people tested only for groups at very high risk of infection (e.g. people on evacuation flights from Wuhan, Hubei province, China and households with high attack rates) | **Exposure phase(s) relative to index case’s infection**  Presymptomatic (7 to 10 days since index case was infected)  **Precautions/controls used**  Isolation of confirmed cases and daily monitoring of contacts | **Transmission demonstrated**  Uncertain (possibly presymptomatic)  **Attack rate (exposure via index case only)**  Not calculable (possibly at least 1 secondary infection resulting from presymptomatic exposure to index case) | **Clear inclusion criteria:** yes  **Condition measured in standard, reliable way:** yes  **Valid methods used to identify condition:** yes  **Consecutive inclusion:** not applicable  **Complete inclusion:** yes  **Clear reporting of demographics:** yes  **Clear reporting of clinical information:** yes  **Clear reporting of outcomes and follow up:** yes  **Clear reporting of presenting site(s)/clinic(s) demographic information:** not applicable  **Statistical analysis appropriate:** not applicable | Data extracted are for ‘cluster B’; primary aim was to summarise evidence from clusters most likely to demonstrate presymptomatic transmission based on systematic investigation of all confirmed cases in Singapore (7 clusters in total; ‘cluster A’ summarised alongside ‘church A’ of Yong 2020^[53]^ and ‘cluster C’ of Pung 2020;^[36]^ remaining clusters summarised separately) |
| Wei 2020^[46]^ | **Source of infection**  Contact with confirmed case, Singapore  **Date(s) of infection**  26 Feb 2020 | **Setting**  Community (household)  **Location**  Singapore  **Earliest exposure to index case**  27 Feb 2020  **Latest follow up**  5 Mar 2020 | **Identification method**  SARS-CoV-2 RNA (PCR, no further details reported)  **Contact tracing**  Extensive (active case finding and monitoring from onset of symptoms)  **People tested**  Suspected (symptomatic) cases; asymptomatic people tested only for groups at very high risk of infection (e.g. people on evacuation flights from Wuhan, Hubei province, China and households with high attack rates) | **Exposure phase(s) relative to index case’s infection**  Presymptomatic (< 7 days since index case was infected)  **Precautions/controls used**  Isolation of confirmed cases and daily monitoring of contacts | **Transmission demonstrated**  Uncertain (possibly presymptomatic)  **Attack rate (exposure via index case only)**  Not calculable (possibly at least 1 secondary infection resulting from presymptomatic exposure to index case) | **Clear inclusion criteria:** yes  **Condition measured in standard, reliable way:** yes  **Valid methods used to identify condition:** yes  **Consecutive inclusion:** not applicable  **Complete inclusion:** yes  **Clear reporting of demographics:** yes  **Clear reporting of clinical information:** yes  **Clear reporting of outcomes and follow up:** yes  **Clear reporting of presenting site(s)/clinic(s) demographic information:** not applicable  **Statistical analysis appropriate:** not applicable | Data extracted are for ‘cluster C’; primary aim was to summarise evidence from clusters most likely to demonstrate presymptomatic transmission based on systematic investigation of all confirmed cases in Singapore (7 clusters in total; ‘cluster A’ summarised alongside ‘church A’ of Yong 2020^[53]^ and ‘cluster C’ of Pung 2020;^[36]^ remaining clusters summarised separately) |
| Wei 2020^[46]^ | **Source of infection**  Attending singing class involving confirmed case, Singapore  **Date(s) of infection**  27 Feb 2020 (attended church on 1 Mar 2020) | **Setting**  Community (church)  **Location**  Singapore  **Earliest exposure to index case**  1 Mar 2020  **Latest follow up**  5 Mar 2020 | **Identification method**  SARS-CoV-2 RNA (PCR, no further details reported)  **Contact tracing**  Extensive (active case finding and monitoring from onset of symptoms)  **People tested**  Suspected (symptomatic) cases; asymptomatic people tested only for groups at very high risk of infection (e.g. people on evacuation flights from Wuhan, Hubei province, China and households with high attack rates) | **Exposure phase(s) relative to index case’s infection**  Presymptomatic (< 7 days since index case was infected)  **Precautions/controls used**  Isolation of confirmed cases and daily monitoring of contacts | **Transmission demonstrated**  Uncertain (possibly presymptomatic)  **Attack rate (exposure via index case only)**  Not calculable (possibly at least 2 secondary infections resulting from presymptomatic exposure to index case) | **Clear inclusion criteria:** yes  **Condition measured in standard, reliable way:** yes  **Valid methods used to identify condition:** yes  **Consecutive inclusion:** not applicable  **Complete inclusion:** yes  **Clear reporting of demographics:** yes  **Clear reporting of clinical information:** yes  **Clear reporting of outcomes and follow up:** yes  **Clear reporting of presenting site(s)/clinic(s) demographic information:** not applicable  **Statistical analysis appropriate:** not applicable | Data extracted are for ‘cluster F’; primary aim was to summarise evidence from clusters most likely to demonstrate presymptomatic transmission based on systematic investigation of all confirmed cases in Singapore (7 clusters in total; ‘cluster A’ summarised alongside ‘church A’ of Yong 2020^[53]^ and ‘cluster C’ of Pung 2020;^[36]^ remaining clusters summarised separately) |
| Wei 2020^[46]^ | **Source of infection**  Contact with patient with pneumonia, the Philippines  **Date(s) of infection**  23 Feb to 2 Mar 2020 (returned to Singapore on or after 2 Mar 2020) | **Setting**  Community (household)  **Location**  Singapore  **Earliest exposure to index case**  3 Mar 2020  **Latest follow up**  8 Mar 2020 | **Identification method**  SARS-CoV-2 RNA (PCR, no further details reported)  **Contact tracing**  Extensive (active case finding and monitoring from onset of symptoms)  **People tested**  Suspected (symptomatic) cases; asymptomatic people tested only for groups at very high risk of infection (e.g. people on evacuation flights from Wuhan, Hubei province, China and households with high attack rates) | **Exposure phase(s) relative to index case’s infection**  Presymptomatic (days since index case was infected not calculable, but <= 13 days since index case was infected)  **Precautions/controls used**  Isolation of confirmed cases and daily monitoring of contacts | **Transmission demonstrated**  Uncertain (possibly presymptomatic)  **Attack rate (exposure via index case only)**  Not calculable (possibly at least 1 secondary infection resulting from presymptomatic exposure to index case) | **Clear inclusion criteria:** yes  **Condition measured in standard, reliable way:** yes  **Valid methods used to identify condition:** yes  **Consecutive inclusion:** not applicable  **Complete inclusion:** yes  **Clear reporting of demographics:** yes  **Clear reporting of clinical information:** yes  **Clear reporting of outcomes and follow up:** yes  **Clear reporting of presenting site(s)/clinic(s) demographic information:** not applicable  **Statistical analysis appropriate:** not applicable | Data extracted are for ‘cluster D’; primary aim was to summarise evidence from clusters most likely to demonstrate presymptomatic transmission based on systematic investigation of all confirmed cases in Singapore (7 clusters in total; ‘cluster A’ summarised alongside ‘church A’ of Yong 2020^[53]^ and ‘cluster C’ of Pung 2020;^[36]^ remaining clusters summarised separately) |
| Hamner 2020^[18]^ | **Source of infection**  Not reported (index case assumed to be choir member who attended practice on 3 Mar 2020 and developed symptoms on 7 Mar 2020)  **Date(s) of infection**  Before 7 Mar 2020 | **Setting**  Community (choir practice)  **Location**  Skagit County, Washington, USA  **Earliest exposure to index case**  3 Mar 2020 (or before)  **Latest follow up**  10 Apr 2020 | **Identification method**  SARS-CoV-2 RNA (RT-PCR) or clinical (symptoms suggesting infection)  **Contact tracing**  Limited (choir members only)  **People tested**  Index case and some other choir members (no further details reported) | **Exposure phase(s) relative to index case’s infection**  Presymptomatic (days since index case was infected not calculable)  Symptomatic  **Precautions/controls used**  Self-isolation/quarantine of some choir members from 17 Mar 2020 in response to email from choir director confirming infection in at least 1 member of the choir and emphasising importance of social distancing and awareness of symptoms; formal instruction to quarantine from 18 Mar 2020 for choir members who had not already developed symptoms | **Transmission demonstrated**  Uncertain (possibly presymptomatic)  **Attack rate (exposure via index case only)**  5% (1/21) among choir members who attended practice only on 3 Mar 2020; the single case in the numerator did not receive a laboratory test, but had symptoms suggesting infection (95% CI 0.1% to 24%; calculated by HIS team) | **Clear inclusion criteria:** yes  **Condition measured in standard, reliable way:** unclear  **Valid methods used to identify condition:** unclear  **Consecutive inclusion:** not applicable  **Complete inclusion:** yes  **Clear reporting of demographics:** unclear  **Clear reporting of clinical information:** yes  **Clear reporting of outcomes and follow up:** yes  **Clear reporting of presenting site(s)/clinic(s) demographic information:** not applicable  **Statistical analysis appropriate:** not applicable | Data extracted are for choir practice on 3 Mar 2020; article highlighted choir practice on 10 Mar 2020 as being more likely transmission event from symptomatic index case to other members of the choir (further analyses related to 10 Mar 2020 choir practice not extracted); exposure histories of index case and other choir members unclear (2/37 choir members attended neither the 3 nor 10 Mar 2020 practices, but developed symptoms suggesting infection) |
| Wei 2020^[46]^ | **Source of infection**  Travelling to Indonesia  **Date(s) of infection**  3 to 7 Mar 2020 (returned to Singapore on or after 7 Mar 2020) | **Setting**  Community (index case ‘met a woman’, no further details reported)  **Location**  Singapore  **Earliest exposure to index case**  8 Mar 2020  **Latest follow up**  12 Mar 2020 | **Identification method**  SARS-CoV-2 RNA (PCR, no further details reported)  **Contact tracing**  Extensive (active case finding and monitoring from onset of symptoms)  **People tested**  Suspected (symptomatic) cases; asymptomatic people tested only for groups at very high risk of infection (e.g. people on evacuation flights from Wuhan, Hubei province, China and households with high attack rates) | **Exposure phase(s) relative to index case’s infection**  Presymptomatic (< 7 days since index case was infected)  **Precautions/controls used**  Isolation of confirmed cases and daily monitoring of contacts | **Transmission demonstrated**  Uncertain (possibly presymptomatic)  **Attack rate (exposure via index case only)**  Not calculable (possibly at least 1 secondary infection resulting from presymptomatic exposure to index case) | **Clear inclusion criteria:** yes  **Condition measured in standard, reliable way:** yes  **Valid methods used to identify condition:** yes  **Consecutive inclusion:** not applicable  **Complete inclusion:** yes  **Clear reporting of demographics:** yes  **Clear reporting of clinical information:** yes  **Clear reporting of outcomes and follow up:** yes  **Clear reporting of presenting site(s)/clinic(s) demographic information:** not applicable  **Statistical analysis appropriate:** not applicable | Data extracted are for ‘cluster G’; primary aim was to summarise evidence from clusters most likely to demonstrate presymptomatic transmission based on systematic investigation of all confirmed cases in Singapore (7 clusters in total; ‘cluster A’ summarised alongside ‘church A’ of Yong 2020^[53]^ and ‘cluster C’ of Pung 2020;^[36]^ remaining clusters summarised separately) |
| Baettig 2020^[5]^ | **Source of infection**  Recreational visit to the Alps, Switzerland  **Date(s) of infection**  8 Mar 2020 | **Setting**  Community (military recruit school)  **Location**  Monte Ceneri, Ticino, Switzerland  **Earliest exposure to index case**  8 Mar 2020  **Latest follow up**  27 Mar 2020 | **Identification method**  SARS-CoV-2 RNA (PCR, no further details reported)  **Contact tracing**  Limited (contacts in military school only)  **People tested**  Index case and symptomatic contacts | **Exposure phase(s) relative to index case’s infection**  Presymptomatic (< 7 days since index case was infected)  **Precautions/controls used**  Isolation of index case after infection confirmed; quarantine of close contacts (< 2 metres for > 15 minutes in the 48 hours before index case developed symptoms) in separate  military barrack | **Transmission demonstrated**  Uncertain (possibly presymptomatic)  **Attack rate (exposure via index case only)**  2% (1/55); another 6 people with symptoms suggesting infection tested negative using PCR (95% CI 0.05% to 10%; calculated by HIS team) | **Clear inclusion criteria:** unclear  **Condition measured in standard, reliable way:** yes  **Valid methods used to identify condition:** yes  **Consecutive inclusion:** not applicable  **Complete inclusion:** yes  **Clear reporting of demographics:** yes  **Clear reporting of clinical information:** yes  **Clear reporting of outcomes and follow up:** yes  **Clear reporting of presenting site(s)/clinic(s) demographic information:** yes  **Statistical analysis appropriate:** not applicable | Possibility of symptomatic exposure of index case’s close contacts not reported (contact tracing based on index case’s presymptomatic phase) |
| Wei 2020^[46]^ | **Source of infection**  Travelling to Japan  **Date(s) of infection**  29 Feb to 8 Mar 2020 (returned to Singapore on or after 8 Mar 2020) | **Setting**  Community (household)  **Location**  Singapore  **Earliest exposure to index case**  9 Mar 2020  **Latest follow up**  11 Mar 2020 | **Identification method**  SARS-CoV-2 RNA (PCR, no further details reported)  **Contact tracing**  Extensive (active case finding and monitoring from onset of symptoms)  **People tested**  Suspected (symptomatic) cases; asymptomatic people tested only for groups at very high risk of infection (e.g. people on evacuation flights from Wuhan, Hubei province, China and households with high attack rates) | **Exposure phase(s) relative to index case’s infection**  Presymptomatic (days since index case was infected not calculable, but <= 9 days since index case was infected)  **Precautions/controls used**  Isolation of confirmed cases and daily monitoring of contacts | **Transmission demonstrated**  Uncertain (possibly presymptomatic)  **Attack rate (exposure via index case only)**  Not calculable (possibly at least 1 secondary infection resulting from presymptomatic exposure to index case) | **Clear inclusion criteria:** yes  **Condition measured in standard, reliable way:** yes  **Valid methods used to identify condition:** yes  **Consecutive inclusion:** not applicable  **Complete inclusion:** yes  **Clear reporting of demographics:** yes  **Clear reporting of clinical information:** yes  **Clear reporting of outcomes and follow up:** yes  **Clear reporting of presenting site(s)/clinic(s) demographic information:** not applicable  **Statistical analysis appropriate:** not applicable | Data extracted are for ‘cluster E’; primary aim was to summarise evidence from clusters most likely to demonstrate presymptomatic transmission based on systematic investigation of all confirmed cases in Singapore (7 clusters in total; ‘cluster A’ summarised alongside ‘church A’ of Yong 2020^[53]^ and ‘cluster C’ of Pung 2020;^[36]^ remaining clusters summarised separately) |
| Ravaioli 2020^[40]^ | **Source of infection**  Not reported  **Date(s) of infection**  Not reported | **Setting**  Nosocomial (transplant surgery department)  **Location**  Bologna, Emilia-Romagna, Italy  **Earliest exposure to index case**  Not reported (article received 6 Apr 2020, first published 5 May 2020)  **Latest follow up**  Not reported (article received 6 Apr 2020, first published 5 May 2020) | **Identification method**  SARS-CoV-2 RNA (RT-PCR)  **Contact tracing**  Limited (patients on whom surgeon had operated during presymptomatic phase and clinical colleagues)  **People tested**  Index case (surgeon) and contacts (patients and clinical colleagues) | **Exposure phase(s) relative to index case’s infection**  Presymptomatic (days since index case was infected not calculable)  **Precautions/controls used**  Home quarantine for surgeon once infection confirmed, PPE used while working in presymptomatic phase (hand hygiene, surgical mask and gloves for preoperative visits and standard surgical procedures); clinical colleagues used PPE (surgical masks when distance < 1 metre and gloves for any contact) | **Transmission demonstrated**  No (exposure to presymptomatic case with PPE in use)  **Attack rate (exposure via index case only)**  0% (0/28) among patients on whom surgery performed by index case and clinical colleagues, with PPE in use (95% CI 0% to 12%; calculated by HIS team) | **Clear inclusion criteria:** yes  **Condition measured in standard, reliable way:** yes  **Valid methods used to identify condition:** yes  **Consecutive inclusion:** yes  **Complete inclusion:** yes  **Clear reporting of demographics:** unclear  **Clear reporting of clinical information:** yes  **Clear reporting of outcomes and follow up:** unclear  **Clear reporting of presenting site(s)/clinic(s) demographic information:** unclear  **Statistical analysis appropriate:** not applicable | None |

Studies listed in chronological order; grey shading indicates consideration of evidence from outside mainland China; italics indicate consideration of asymptomatic transmission
CI confidence interval; COVID-19 coronavirus disease 2019; CT computerised tomography; ELISA enzyme-linked immunosorbent assay; HIS Healthcare Infection Society; PCR polymerase chain reaction; PPE personal protective equipment; RNA ribonucleic acid; RT-PCR reverse transcription PCR; SARS-CoV-2 severe acute respiratory syndrome coronavirus 2; VNT virus neutralisation test

^a^ Assessed using Joanna Briggs Institute critical appraisal checklist for case series (response options for each criterion are yes/no/unclear/not applicable)

**Table G.2** Comparative observational studies investigating relative transmissibility

| **Study identifier** | **Index case(s) and study design** | **Associated cluster/outbreak** | **Case identification and contact tracing** | **Exposure and precautions/controls** | **Transmission outcomes** | **Study quality^a^** | **Reviewer comments** |
| --- | --- | --- | --- | --- | --- | --- | --- |
| Chen 2020;^[11]^ He 2020a;^[19]^ Yin 2020^[52]^ | **Source of infection**  Not reported (but refers to undiagnosed imported cases before 14 Jan 2020; multiple index cases, including 1 super-spreader (resulting in at least 10 secondary infections), identified via active surveillance – see under study design below)  **Date(s) of infection**  Not reported  **Study design**  Observational (prospective index case identification with contact tracing via ‘continuous isolation medical observations of new coronavirus pneumonia cases and close contacts of asymptomatic infections’ (using contract tracing surveillance data; He 2020a))^[19]^  **Selection criteria**  All 161 confirmed (symptomatic; He 2020a)^[19]^ cases and 30 asymptomatic cases between 21 Jan and 6 Mar 2020  **Demographic characteristics**  Age: not reported  Gender: not reported  **Clinical characteristics**  Pre-existing conditions: not reported  COVID-19 severity: not reported  **Consent**  Not reported (but study granted ethical approval) | **Setting**  Community (households, family gatherings, religious gatherings, other social contacts (e.g. meeting friends), work, education, shopping, shared transport, etc) and nosocomial (via contact with healthcare settings)  **Location**  Ningbo, Zhejiang province, China  **Earliest exposure to index case**  Not reported (first case confirmed 21 Jan 2020; ‘earliest onset’ traced back to 14 Jan 2020)  **Latest follow up**  Not reported (identification of index cases ended 6 Mar 2020) | **Identification method**  SARS-CoV-2 RNA (nucleic acid, no further details reported)  **Contact tracing**  Extensive (‘close contacts with the case in accordance with clear contact time and the contact time is short, no other close contacts of exposure or exposure history and other relevant principles of judgment chosen in some cases to estimate the incubation period’); total 2147 close contacts  **People tested**  Not reported (but see under precautions/controls used to right)  **Contacts’ demographic characteristics**  Age: not reported  Gender: not reported  **Contacts’ clinical characteristics**  Pre-existing conditions: not reported | **Exposure phase(s) relative to index case’s infection**  Asymptomatic (days since index case was infected not calculable; asymptomatic not defined)  Symptomatic  **Precautions/controls used**  Centralised/home ‘isolation’ of close contacts, with medical observation; for super-spreaders, medical observation period extended to 21 days and testing frequency increased | **Transmission demonstrated**  Yes (asymptomatic)  **Attack rate**  Symptomatic exposure (close contacts of 161 confirmed/symptomatic cases), 6.30% (126/2001) versus asymptomatic exposure (close contacts of 30 asymptomatic cases), 4.11% (6/146)  Chi-squared = 1.28, p = 0.288  RR = 1.5 (95% CI 0.7 to 3.4); He 2020a^[19]^  Fisher’s exact test, p = 0.84 including secondary infections associated with super-spreader, or p = 0.37 excluding secondary infections associated with super-spreader (Yin 2020)^[52]^  OR = 1.568 (95% CI 0.679 to 3.620) including secondary infections associated with super-spreader, or OR = 1.212 (95% CI 0.522 to 2.815) excluding secondary infections associated with super-spreader (Yin 2020)^[52]^  Yin 2020^[52]^ also reported absolute differences in transmission rates (with 95% CIs)  **Reproduction number**  Overall: 0.78 (126/161) among symptomatic cases versus 0.20 (6/30) among asymptomatic cases, RR = 3.9 (95% CI 1.5 to 11.8); He 2020a^[19]^  Symptomatic secondary infections only: RR = 6.6 (95%  CI 2.0 to 34.7); reproduction numbers for symptomatic and asymptomatic infections not reported; He 2020a^[19]^  **Relative transmissibility according to symptom status**  Asymptomatic exposure (6/146 contacts tested positive, regardless of whether or not the contacts had symptoms) versus symptomatic exposure (126/2001 contacts tested positive, regardless of whether or not the contacts had symptoms, or 94/1904 if super-spreader and associated contacts excluded), OR = 0.64 (95% CI 0.28 to 1.47), or OR = 0.83 (95% CI 0.36 to 1.92) if super-spreader excluded; calculated by HIS team | **Validity**  Clearly focused issue: yes  Appropriate method used: yes  Cases recruited in acceptable way: yes  Controls selected in acceptable way: yes  Exposure measured accurately: can’t tell  Confounding factors accounted for in design/analysis: no  **Results**  Precise results/estimate of risk: yes  Believable results: yes  **Applicability**  Results applicable to local population: yes  Results fit with other available evidence: yes | Unless otherwise stated data extracted from Chen 2020^[11]^ (article published in Chinese and incomplete translation might account for some lack of clarity in reporting; estimated incubation period also reported, but not relevant to this review); He 2020a^[19]^ and Yin 2020^[52]^ reported independent re-analyses of the same data  He 2020a^[19]^ noted that symptomatic cases might be infectious for longer than asymptomatic cases and that this might explain the difference in results based on RRs estimated via attack rates and those estimated via reproduction numbers; He 2020a^[19]^ also noted that the greater number of contacts among symptomatic cases (compared to asymptomatic cases) might reflect a real difference in contact patterns (also noted by Chen 2020)^[11]^ or it might be an artefact of the contact tracing system, in which case the RR might be over-estimated; Yin 2020^[52]^ also noted the differences in numbers of contacts traced for symptomatic and asymptomatic cases |
| Cheng 2020^[12]^ | **Source of infection**  Not reported (multiple index cases identified via Taiwan National Notifiable Disease Surveillance System)  **Date(s) of infection**  Not reported  **Study design**  Observational (prospective index case identification with contact tracing)  **Selection criteria**  First 100 confirmed cases (15 Jan to 18 Mar 2020)  **Demographic characteristics**  Age: median 44 years (range 11 to 88 years)  Gender: 56 male, 44 female  **Clinical characteristics**  Pre-existing conditions: not reported  COVID-19 severity: classified according to World Health Organization interim guidance  **Consent**  Informed consent waived as part of  public health response/surveillance | **Setting**  Community (household, family gatherings, other social contacts (e.g. meeting friends), shared transport, etc) and nosocomial (via contact with healthcare settings)  **Location**  Taiwan  **Earliest exposure to index case**  Not reported (first case confirmed 21 Jan 2020 and contacts up to 4 days before symptom onset investigated)  **Latest follow up**  2 Apr 2020 | **Identification method**  SARS-CoV-2 RNA (RT-PCR)  **Contact tracing**  Comprehensive (all close contacts, defined as people having face-to-face contact with a confirmed case during the investigation period for > 15 minutes without appropriate PPE, including household/family members and hospital staff/patients where relevant (no minimum contact time for hospital staff/patients); contacts more than 4 days before symptom onset (or confirmed infection for asymptomatic index cases) excluded; total 2761 close contacts followed up until 14 days after the last exposure to the index case  **People tested**  People meeting COVID-19 notification criteria, including index cases, their household/hospital contacts, and other contacts with suspected (symptomatic) infection  **Contacts’ demographic characteristics**  Age: mean/median not reported (range 0 to 96 years)  Gender: 1155 male, 1437 female, 169 unknown  **Contacts’ clinical characteristics**  Pre-existing conditions: not reported | **Exposure phase(s) relative to index case’s infection**  Presymptomatic (days since index case was infected not calculable)  Asymptomatic (days since index case was infected not calculable; asymptomatic not defined)  Symptomatic  **Precautions/controls used**  Hospital isolation of confirmed cases and home quarantine of close contacts; appropriate PPE determined by exposure setting and procedures performed (e.g. N95 respirators required when performing aerosol-generating procedures) | **Transmission demonstrated**  Yes (presymptomatic)  No (asymptomatic)  **Attack rate**  Presymptomatic exposure only: 0.7% (2/299), 95% CI 0.2% to 2.4%, number of index cases not reported; relative transmissibility compared to any symptomatic exposure, RR = 0.99 (95% CI 0.23 to 4.29); attack rate defined as ratio of symptomatic confirmed cases to close contacts (i.e. excludes asymptomatic secondary infections)  Asymptomatic exposure only: 0% (0/91) among close contacts of 9 asymptomatic cases  **Relative transmissibility according to symptom status**  Presymptomatic exposure (2/299 contacts tested positive, regardless of whether or not the contacts had symptoms) versus symptomatic exposure (20/2371 contacts tested positive, regardless of whether or not the contacts had symptoms), OR = 0.79 (95% CI 0.18 to 3.40); calculated by HIS team  Asymptomatic exposure (0/91 contacts tested positive, regardless of whether or not the contacts had symptoms) versus symptomatic exposure (20/2371 contacts tested positive, regardless of whether or not the contacts had symptoms), OR = 0.63 (95% CI 0.04 to 10.44); calculated by HIS team | **Validity**  Clearly focused issue: yes  Appropriate method used: yes  Cases recruited in acceptable way: yes  Controls selected in acceptable way: yes  Exposure measured accurately: can’t tell  Confounding factors accounted for in design/analysis: no  **Results**  Precise results/estimate of risk: yes  Believable results: yes  **Applicability**  Results applicable to local population: yes  Results fit with other available evidence: yes | Risk of transmission stratified by exposure setting and interval between index case’s symptom onset and contacts’ first day of exposure (e.g. to compare household transmissibility with other exposure settings based on any presymptomatic exposure) also reported, but cannot rule out some symptomatic exposure and so data not extracted; estimated serial interval and incubation period also reported, but not relevant to this review; cannot rule out overlap between study population and individual clusters/outbreaks in Taiwan summarised in Table G.1 |
| Zhang 2020b^[56]^ | **Source of infection**  Not reported (multiple index cases identified via contact tracing surveillance)  **Date(s) of infection**  Not reported  **Study design**  Observational (analysis of contact tracing surveillance data, but whether prospective or retrospective not reported)  **Selection criteria**  All 83 confirmed cases without symptoms at diagnosis between 28 Jan and 15 Mar 2020  **Demographic characteristics**  Age: not reported  Gender: reported only for index cases associated with confirmed secondary cases (6 male, 6 female)  **Clinical characteristics**  Pre-existing conditions: not reported  COVID-19 severity: reported only for index cases associated with confirmed secondary cases; classification criteria (for mild, moderate and severe symptoms) not reported  **Consent**  Informed consent waived (contact tracing surveillance data used), although authors noted under ‘management process’ that written/verbal information (including study rationale, duration, legal basis, precautions, disease-related medical observation during quarantine and contact information for responsible authorities) was provided to contacts | **Setting**  Community (household, family gatherings, meeting friends, other social/recreational events, shared transport, etc) and nosocomial (through contact with healthcare settings)  **Location**  Guangzhou, Guangdong province, China  **Earliest exposure to index case**  Not reported (contact tracing surveillance started 28 Jan 2020)  **Latest follow up**  Not reported (contact tracing surveillance ended 15 Mar 2020) | **Identification method**  SARS-CoV-2 RNA (nucleic acid, no further details reported)  **Contact tracing**  Extensive (close contacts of presymptomatic cases identified through active surveillance, including household members and extended family, carers, healthcare workers (including those involved in aerosol-generating procedures), work colleagues, people who shared small spaces such as entertainment venues or transport); close contact defined as having contact with an index case up to 2 days before the index case was tested and without using ‘proper protection’ (usually meaning wearing a surgical mask consistently while in contact with the index case); close contacts of contacts with confirmed/suspected infection traced recursively; close contacts exposed to > 2 confirmed cases excluded; total 369 close contacts  **People tested**  All close contacts (tested at the beginning and end of medical observation; additional testing for those who developed symptoms during quarantine)  **Contacts’ demographic characteristics**  Age: median 35 years (range 0 to 93 years)  Gender: reported only for confirmed cases (5 male, 7 female)  **Contacts’ clinical characteristics**  Pre-existing conditions: not reported | **Exposure phase(s) relative to index case’s infection**  Presymptomatic (days since index case was infected not calculable)  Asymptomatic (no clinically identifiable signs of infection throughout 14-day quarantine; days since index case was infected not calculable)  **Precautions/controls used**  Centralised (e.g. hotel) quarantine of close contacts; transfer to ‘medical institution’ for confirmed/suspected cases | **Transmission demonstrated**  Yes (presymptomatic and asymptomatic)  **Attack rate**  0.8% (1 contact tested positive, denominator (total number of contacts) not reported) among close contacts of asymptomatic cases, compared with 3.5% (5 contacts tested positive, denominator not reported) among close contacts of presymptomatic cases who later developed mild symptoms, 5.7% (5 contacts tested positive, denominator not reported) among close contacts of  presymptomatic cases who later developed moderate symptoms, and 4.5% (1 contact tested positive, denominator not reported) among close contacts of presymptomatic cases who later developed severe symptoms; attack rate refers to any contact who tested positive, regardless of whether or not they had symptoms  **Relative transmissibility according to symptom status**  Asymptomatic exposure (1/125 contacts tested positive, regardless of whether or not the contacts had symptoms) versus presymptomatic exposure (11/244 contacts tested positive, regardless of whether or not the contacts had symptoms), OR = 0.17 (95% CI 0.02 to 1.34); calculated by HIS team (attack rates according to symptom status imputed by HIS team) | **Validity**  Clearly focused issue: yes  Appropriate method used: yes  Cases recruited in acceptable way: yes  Controls selected in acceptable way: yes  Exposure measured accurately: yes  Confounding factors accounted for in design/analysis: no  **Results**  Precise results/estimate of risk: can’t tell  Believable results: yes  **Applicability**  Results applicable to local population: yes  Results fit with other available evidence: yes | Cannot rule out overlap between study population and individual clusters/outbreaks in Guangzhou, Guangdong province, China summarised in Table G.1 |
| Park 2020^[35]^ | **Source of infection**  Unknown  **Date(s) of infection**  Unknown (but before first case developed symptoms on 22 Feb 2020)  **Study design**  Observational (retrospective investigation of a single cluster/outbreak with contact tracing)  **Selection criteria**  Not applicable (but all 1145 people working/living in or visiting building where cluster/outbreak identified between 21 Feb and 8 Mar 2020 were investigated)  **Demographic characteristics**  See under contact tracing to right  **Clinical characteristics**  See under contact tracing to right  **Consent**  Informed consent waived as part of  public health response/surveillance | **Setting**  Community (call centre in commercial/residential building and associated households)  **Location**  Seoul, South Korea  **Earliest exposure to index case**  21 Feb 2020 (or before)  **Latest follow up**  3 Apr 2020 (or after) | **Identification method**  SARS-CoV-2 RNA (RT-PCR)  **Contact tracing**  Comprehensive (all call centre workers, other people living/working in the building, visitors and household contacts of confirmed cases); total number of contacts not reported  **People tested**  1143 people associated with building containing call centre (922 employees, 201 residents and 20 visitors; another 2 residents not tested); also 955 household contacts of confirmed cases regardless of whether contacts symptomatic  **Contacts’ demographic characteristics**  Age: mean 38 years (range 30 to 80 years (employees, residents and visitors only)  Gender: 237 male, 620 female, 286 unknown (employees, residents and visitors only)  **Contacts’ clinical characteristics**  Pre-existing conditions: not reported | **Exposure phase(s) relative to index case’s infection**  Presymptomatic (defined as having no symptoms at the time of positive test result, but developed symptoms during the 14 days of monitoring; days since index case was infected not calculable)  Asymptomatic (defined as having no symptoms at the time of positive test result nor during the 14 days of monitoring); days since index case was infected not calculable)  Symptomatic (defined as having symptoms at the time of positive test result)  **Precautions/controls used**  Building closed on 9 Mar 2020 (immediately after Korea Centers for Disease Control and Prevention notified of cluster/outbreak) with testing offered to all people working/living there and visitors; isolation of confirmed cases, quarantine of test-negative contacts; text messages sent to people who had been near the building for > 5 minutes (identified via mobile phone tracking; total 16 628 messages) with instructions to avoid contact with other people and seek testing; investigation, testing and monitoring of household contacts of all confirmed cases  during 14-day quarantine | **Transmission demonstrated**  No (presymptomatic/asymptomatic; authors noted that isolation/quarantine might have interrupted transmission, and so presymptomatic/asymptomatic transmission could not be ruled out)  **Attack rate**  0% (0/11) among household members of 4 presymptomatic cases, 0% (0/4; reported as 0/17 in the authors’ discussion, but this is inconsistent with overall number of contacts investigated) among household members of 4 asymptomatic cases, and 16.2% (34/210) among household members of all 89 symptomatic cases (95% CI 11.6% to 22.0%)  **Relative transmissibility according to symptom status**  Presymptomatic exposure (0/11 contacts tested positive, regardless of whether or not the contacts had symptoms) versus symptomatic exposure (34/210 contacts tested positive, regardless of whether or not the contacts had symptoms), OR = 0.22 (95% CI 0.01 to 3.86); calculated by HIS team  Asymptomatic exposure (0/4 contacts tested positive, regardless of whether or not the contacts had symptoms) versus symptomatic exposure (34/210 contacts tested positive, regardless of whether or not the contacts had symptoms), OR = 0.57 (95% CI 0.03 to 10.80); calculated by HIS team | **Validity**  Clearly focused issue: yes  Appropriate method used: yes  Cases recruited in acceptable way: yes  Controls selected in acceptable way: yes  Exposure measured accurately: yes  Confounding factors accounted for in design/analysis: no  **Results**  Precise results/estimate of risk: can’t tell  Believable results: yes  **Applicability**  Results applicable to local population: yes  Results fit with other available evidence: yes | Authors noted that mass testing might have prevented asymptomatic transmission because asymptomatic cases received information about their possible infection and therefore might have self-isolated from their household members |

Studies listed in chronological order; grey shading indicates consideration of evidence from outside mainland China
CI confidence interval; COVID-19 coronavirus disease 2019; HIS Healthcare Infection Society; OR odds ratio; PCR polymerase chain reaction; PPE personal protective equipment; RNA ribonucleic acid; RR relative risk; RT-PCR reverse transcription PCR; SARS-CoV-2 severe acute respiratory syndrome coronavirus 2

^a^ Assessed using Critical Appraisal Skills Programme case–control study checklist (response options for each criterion are yes/no/can’t tell); the studies in this table were treated as nested case–control studies for the purpose of estimating relative transmissibility

**Table G.3** Mathematical modelling studies investigating relative/absolute transmissibility

| **Study identifier** | **Model overview** | **Model structure** | **Assumptions and inputs** | **Model fitting and validation** | **Transmission risk estimates** | **Study quality** | **Reviewer comments** |
| --- | --- | --- | --- | --- | --- | --- | --- |
| Li 2020c^[29]^ | **Context**  Spatial and temporal modelling of SARS-CoV-2 transmission in China  **Approach**  Adaptation of SEIR  compartmental model based on a network of Chinese cities,  allowing for spatial mobility of the population  **Purpose**  Estimation of parameters of epidemic spread, such as the reproduction number, under various containment strategies applied in China in response to the epidemic, and exploration of the impact on transmission of undocumented infections  **Time period modelled**  10 Jan to 8 Feb 2020 | **Model states**  Susceptible, exposed, documented  infected (symptoms severe  enough to be confirmed/observed), and undocumented infected (lacking symptoms severe enough to be confirmed/observed)  **Model specification**  Ordinary differential equations  **Parameterisation in relation to symptom status**  Documented infected transmission rate, and (via a scaling factor) undocumented infection transmission rate  **Containment strategies**  Travel restrictions between Wuhan, Hubei province, China and other major cities in China from 23 Jan 2020, plus self-quarantine, contact precautions and more rapid access to testing for confirmation of infection  **Time-varying components**  Population mobility (estimated from mobile phone tracking during 2018 spring festival, adjusted to allow for reduced mobility after introduction of travel restrictions on 23 Jan 2020), and reporting delay (transition from exposed to infected states) reflecting increased awareness of infection risk and care-seeking behaviour as outbreak progressed | **Key assumptions**  Mobility patterns during 2020 spring festival mirror those during 2018 festival; reporting delay constant from person to person within specified time periods  **Data inputs**  Chinese national COVID-19 data (daily numbers of confirmed cases for 375 Chinese cities), Chinese human mobility data | **Estimation method**  Stochastic integration (using Runge-Kutta method) and iterated ensemble-adjustment Kalman filter  with Bayesian computation (to obtain maximum likelihood estimates)  **Uncertainty**  Incorporated via epidemiological data (see under data inputs to left), different initial values for mean of reporting delay distribution (6 to 10 days) and seeding of the epidemic (maximum number of initial infections, 1500 to 2500), and prior distributions for parameters estimated during model fitting  **Sensitivity analysis**  Simulations using additional reporting delay (2 days added to each simulation generated using the reporting delay distribution), larger maximum seeding (3000), and using normal (instead of uniform) distributions for priors  **Validation**  Synthetic testing of model-inference framework (using simulations to reproduce specified parameter values); repeated simulations for each combination of reporting delay and seeding then 2000 outbreaks simulated using mean parameter estimates and results compared with observed numbers and spatial distribution of confirmed cases; external validation using infection rates among people evacuated from China to other countries during the epidemic | **Absolute risks of transmission in relation to symptom status**  Reported only for documented infected state, transmission rate 1.12 (95% CrI 1.07 to 1.17) during 10 to 23 Jan 2020, 0.51 (95% CrI 0.37 to 0.68) during 24 Jan to 3 Feb 2020, and 0.35 (95% CrI 0.30 to 0.52) during 24 Jan to 8 Feb 2020  **Relative transmissibility according to symptom status**  Relative transmission rate for undocumented infections (compared to documented infections), 0.55 (95% CrI 0.49 to 0.60) during 10 to 23 Jan 2020, 0.47 (95% CrI 0.36 to 0.64) during 24 Jan to 3 Feb 2020, and 0.42 (95% CrI 0.34 to 0.61) during 24 Jan to 8 Feb 2020 | Not assessed | Corrected estimates extracted from supplementary material (corrections as of 25 Mar 2020); undocumented infections include mild, limited and asymptomatic infections; article focused on estimation under assumed conditions |
| Wan 2020^[45]^ | **Context**  Temporal modelling of SARS-CoV-2 transmission in China, excluding Hubei province  **Approach**  Adaptation of SEIR compartmental model based on national data for mainland China, excluding Hubei province  **Purpose**  Evaluation of effectiveness of containment strategies, estimation of the impact of partial lifting of control measures, and prediction of the epidemic spread in mainland China, excluding Hubei province  **Time period modelled**  20 Jan to 3 Mar 2020 | **Model states**  Susceptible, exposed, infectious with symptoms, infectious but asymptomatic, isolated susceptible, quarantined infected awaiting confirmation, hospitalised, recovered (including recovered after being hospitalised), and dead  **Model specification**  Ordinary differential equations  **Parameterisation in relation to symptom status**  Infectious with symptoms transmission rate, and (via a scaling factor) asymptomatic infectious transmission rate  **Containment strategies**  Integrated restrictions and self-protection measures, including travel restrictions, quarantine on arrival, contact tracing followed by quarantine/isolation, and reduced contact (e.g. wearing a mask)  **Time-varying components**  Contact rate (contacts decrease as total number of reported cases increases) | **Key assumptions**  Recovered state confers immunity throughout epidemic period, no births, no deaths due to non-epidemic causes, initial number of people in susceptible state equal to population of mainland China, excluding Hubei province  **Data inputs**  Chinese national COVID-19 data, excluding Hubei province (cumulative confirmed cases, cumulative deaths, newly confirmed cases, and cumulative recovered cases) | **Estimation method**  MCMC (convergence assessed using Geweke diagnostic criterion  **Uncertainty**  Via MCMC (prior distributions not reported)  **Sensitivity analysis**  Not reported  **Validation**  Data from 20 Jan to 24 Feb 2020 used to fit model, and data from 25 Feb to 3 Mar 2020 used to compare model predictions and observed data (visual comparisons) | **Absolute risks of transmission in relation to symptom status**  Reported only for infected symptomatic state, transmission rate 0.05 (95% CrI not reported)  **Relative transmissibility according to symptom status**  Relative transmission rate for infectious but asymptomatic (compared to infectious with symptoms), 0.81 (95% CrI not reported) | Not assessed | Authors noted that a time-varying recovery rate might provide a better fit to the data, and that age-related mortality and recovery rates might be more realistic; article combined estimation under assumed conditions and prediction of impact on epidemic spread by controlling contact rates |
| Gatto 2020^[16]^ | **Context**  Spatial and temporal modelling of SARS-CoV-2 transmission in Italy  **Approach**  Adaptation of SEIR  compartmental model based on a network of Italian provinces,  allowing for spatial mobility of the population  **Purpose**  Evaluation of the impact on transmission of specific containment strategies to support emergency planning in Italy (e.g. estimation of hospital cases or deaths averted)  **Time period modelled**  21 Feb to 25 Mar 2020 | **Model states**  Susceptible, exposed, presymptomatic, infected with severe symptoms, asymptomatic or with mild symptoms, hospitalised, in home quarantine, recovered, and dead  **Model specification**  Ordinary differential equations  **Parameterisation in relation to symptom status**  Presymptomatic transmission rate, asymptomatic transmission rate, and symptomatic transmission rate  **Containment strategies**  Successive restrictions (including strict local/national lockdown or social distancing without lockdown) implemented in Italy from 21 Feb to 25 Mar 2020  **Time-varying components**  Presymptomatic transmission rate, reflecting impact of containment measures and increased awareness of infection risk as outbreak progressed | **Key assumptions**  Exposed state is not infectious; presymptomatic, infected with severe symptoms, asymptomatic or with mild symptoms, are infectious states; communities interact in accordance with mobility data (see under data inputs below)  **Data inputs**  Italian national data on COVID-19 hospital admissions, deaths, etc; Italian human mobility data (estimated from historical census data and updated to reflect mobility patterns at the start of 2020); estimated parameters of epidemic spread (durations of latent and infectious periods, serial interval, etc) from published sources, either as point estimates (e.g. proportion of symptomatic cases quarantined averaged across Italy during observed period) or as means and associated CIs | **Estimation method**  MCMC (method of assessing convergence not reported)  **Uncertainty**  Incorporated via epidemiological estimates (see under data inputs to left) and prior distributions for parameters to be estimated via model fitting  **Sensitivity analysis**  Not reported  **Validation**  Graphical comparisons of estimated and observed daily hospitalisations and deaths in Italian regions and nationwide | **Absolute risks of transmission in relation to symptom status**  Not reported  **Relative transmissibility according to symptom status**  Relative transmission rate for presymptomatic infections with containment measures (initial lockdown in parts of northern Italy) introduced on 22 Feb 2020 (compared to presymptomatic infections before 22 Feb 2020), 0.82 (95% CrI 0.77 to 0.86)  Relative transmission rate for presymptomatic infections with additional containment measures (more extensive lockdown in northern Italy and social distancing in other parts of Italy) introduced on 8 Mar 2020 (compared to presymptomatic infections before 22 Feb 2020), 0.66 (95% CrI 0.64 to 0.70)  Relative transmission rate for asymptomatic or with mild symptoms (compared to presymptomatic infections), 0.033 (95% CrI 0.027 to 0.036; article reports upper limit as 0.0036, but extra zero assumed to be a typographical error)  Relative transmission rate for infected with severe symptoms (compared to asymptomatic or with mild symptoms), 1.03 (95% CrI 0.79 to 1.38) | Not assessed | Authors noted that consideration of population age structure in relation to  mobility, social mixing, susceptibility to infection, case fatality ratio, etc could be explored in future extensions of the model; asymptomatic infections and infection with mild symptoms grouped together; article combined estimation under assumed conditions and prediction of impact on epidemic spread of containment measures (in terms of e.g. hospital cases or deaths averted; data not extracted because not relevant to this review) |
| Ferretti 2020^[13]^ | **Context**  Temporal modelling of SARS-CoV-2 transmission in a generic setting (not focused on a specific country)  **Approach**  Renewal equation formulation incorporating authors’ estimated distribution of the generation interval (time between successive infections being acquired) and estimates of other parameters of epidemic spread from published sources  **Purpose**  Estimation of the contribution of different transmission routes to the epidemic spread and determination of speed/scale of case identification and contact tracing needed to control the epidemic  **Time period modelled**  Not applicable (but e.g. estimate of exponential growth rate from which doubling time estimated based on data from China, 25 Jan to 4 Feb 2020) | **Model states**  Not strictly applicable, but modelling allows for presymptomatic, asymptomatic, symptomatic, and environmental transmission (asymptomatic defined as never developing noticeable symptoms, environmental transmission defined as occurring through contamination not accounted for by identifying close contacts)  **Model specification**  Renewal equation (recursive integral equation), weighted by infectivity as a function of time elapsed since previous cases acquired the infection  **Parameterisation in relation to symptom status**  Presymptomatic/symptomatic transmission rate and (via a scaling factor) asymptomatic transmission rate  **Containment strategies**  Identification and isolation of symptomatic cases combined with tracing and quarantining of contacts  **Time-varying components**  Average infectivity (rate of infecting others) is a function of  time since acquiring infection | **Key assumptions**  Infectivity of presymptomatic cases equal to that of symptomatic cases who acquired infection at the same time; infectivity of asymptomatic cases proportional to that of symptomatic cases who acquired infection at the same time; infectivity as a function of time since acquiring infection constant throughout epidemic; susceptible population not depleted through acquired immunity, changing contact patterns, etc during time period modelled; transmission pair data used to fit generation interval distribution representative of presymptomatic/ symptomatic exposure during epidemic; delay of 72 hours between developing symptoms and quarantine of contacts using manual contact tracing  **Data inputs**  Worldwide reports of transmission pairs used to fit generation interval distribution; other key parameters of epidemic spread from published sources, either as point estimates (epidemic doubling time, with associated CI calculated by the authors from source data) or as distributional location/shape parameters, including those used to define priors for parameters estimated during transmission modelling (incubation period and relative infectivity of asymptomatic cases) | **Estimation method**  Maximum composite likelihood for generation interval distribution, using Akaike’s information criterion to compare models with different underlying statistical distributions (Weibull, gamma, and lognormal); sequential Bayesian estimation for transmission modelling (e.g. fitting shape of presymptomatic/symptomatic contributions to overall transmission using estimated generation interval distribution, then calculating relative scaling constant of environmental contribution, and finally calculating overall scaling constant to reproduce observed exponential growth rate); further modelling examined impact of case isolation, contact tracing and quarantine (allowing for reporting delays of up to 72 hours depending on the type of contact tracing used; data not extracted as not relevant to this review)  **Uncertainty**  Incorporated via epidemiological data (see under data inputs to left) and repeated simulations from prior distributions for parameters estimated during model fitting  **Sensitivity analysis**  Not reported (but  different statistical distributions compared for estimation of generation interval (see under estimation method above)  **Validation**  Fitted distributions for generation interval compared graphically with 2 previously  published serial interval distributions and assumed incubation period distribution | **Absolute risks of transmission in relation to symptom status**  Presymptomatic reproduction number, point estimate 0.9, uncertainty median 0.7 (95% CrI 0.2 to 1.1)  Asymptomatic reproduction number, point estimate 0.1, uncertainty median 0.2 (95% CrI 0.0 to 1.2)  Symptomatic reproduction number, point estimate 0.8, uncertainty median 0.6 (95% CrI 0.2 to 1.1)  **Relative transmissibility according to symptom status**  Presymptomatic reproduction number as proportion of total reproduction number, point estimate 0.47, uncertainty median 0.35 (95% CrI 0.11 to 0.58)  Asymptomatic reproduction number as proportion of total reproduction number, point estimate 0.06, uncertainty median 0.09 (95% CrI 0.00 to 0.57)  Symptomatic reproduction number as proportion of total reproduction number, point estimate 0.38, uncertainty median 0.28 (95% CrI 0.09 to 0.49) | Not assessed | Authors noted that real-world identification of symptom status would depend on perceptions of infected people and their contacts (e.g. what is noticeable might differ between parties), that the renewal equation formulation does not allow for recursion across the contact network (i.e. contacts of contacts being traced), and that the model was calibrated using the epidemic growth rate observed in China early in the epidemic, whereas the spread of the epidemic outside China appeared to be faster (perhaps reflecting regional differences in underlying epidemic parameters); point estimate and uncertainty median assumed to be posterior mean and median, respectively; environmental transmission equal to proportion of total reproduction number not accounted for by presymptomatic, asymptomatic or symptomatic transmission (data not extracted because not relevant to this review); article combined estimation under assumed conditions and prediction of impact on epidemic spread of various approaches to contact tracing, including digital contact tracing |
| Buonanno 2020^[8]^ | **Context**  Modelling airborne transmission of SARS-CoV-2 in commercial indoor environments in Italy  **Approach**  Estimation of viral load emitted by an infected person given viral load in the mouth and combinations of respiratory and physical activity, and incorporation of estimates into infection risk model for transmission in indoor commercial environments based on human activity patterns and engineering factors, particularly ventilation characteristics  **Purpose**  Estimation of quanta emission rates (quantum defined as ‘dose of airborne droplet nuclei required to cause infection in 63% of susceptible persons’), and demonstration/prediction of impact of asymptomatic transmission in commercial indoor environments in Italy before/after introduction of containment measures  **Time period modelled**  Not applicable (but cites e.g. containment measures implemented in Italy, 21 Feb to 11 Mar 2020) | **Model states**  Not strictly applicable, but modelling allows for viral load emitted by symptomatic/asymptomatic cases, and transmission risks in indoor commercial environments in the case of asymptomatic cases; quanta emission rates calculated for combinations of 4 expiratory activities (voiced counting, whispered counting, ‘unmodulated vocalization’, and breathing) and 5 physical activity levels (resting, standing, light exercise, moderate exercise, and heavy exercise) across 5 commercial indoor environments (pharmacy, supermarket, restaurant, post office, and bank); inhalation rates of exposed people also taken into account  **Model specification**  Differential equations (modelling quanta emission rates) and integral equation (modelling transmission in indoor environments)  **Parameterisation in relation to symptom status**  Asymptomatic transmission rate  **Containment strategies**  Lockdown restrictions and voluntary measures (e.g. fewer staff on duty, customers queueing outside, and ventilation increased by keeping external doors open) implemented in Italy from 21 Feb to 11 Mar 2020  **Time-varying components**  Numbers of staff and customers, aspects of ventilation, etc varied with containment measures (see under containment strategies above); exposure periods dependent on type of indoor environment (maximum 3.5 hours’ exposure modelled per environment) | **Key assumptions**  Droplets emitted by infected people have same viral load as sputum; SARS-CoV-2 viable in air for up to 3 hours after being aerosolised (half-life 1.1 hours); quanta emission rate constant during exposure period; viral incubation period longer than exposure period in modelled scenarios; droplets instantaneously and evenly distributed throughout the modelled environment; quanta emission rates for asymptomatic cases as for symptomatic cases, but expected activity levels greater (light exercise rather than resting); activity levels for exposed people equivalent to standing or light exercise; model based on ‘worst-case’ scenario for asymptomatic transmission  **Data inputs**  Estimated SARS-CoV-2 viral load in sputum, SARS-CoV-2 inactivation rate (half-life), and generic physical parameters (e.g. particle size distributions for different respiratory activities, and average inhalation rate for each physical activity level) from published sources; corrective coefficient of infectivity (ratio of 1 infectious quantum to infectious dose expressed in viral RNA copies) extrapolated from SARS-CoV data | **Estimation method**  Analytical expression (summation) for quanta emission rates; numerical integration of Wells-Riley equation for modelling asymptomatic transmission in indoor environments (no further details reported)  **Uncertainty**  Not reported (possibly deterministic solution of Wells-Riley equation)  **Sensitivity analysis**  Not reported  **Validation**  Not reported (but credibility of asymptomatic reproduction numbers assessed qualitatively by comparing results for different scenarios) | **Absolute risks of transmission in relation to symptom status**  Asymptomatic reproduction number before lockdown and with natural ventilation, 3.70 in pharmacy, 2.19 in supermarket, 47.3 in restaurant, 3.64 in post office, and 3.52 in bank (95% CIs not reported)  Asymptomatic reproduction number before lockdown and with mechanical ventilation, 5.35 in restaurant (other environments not reported in detail, but ranged from 1.16 to 1.30; 95% CIs not reported)  Asymptomatic reproduction number after lockdown and with natural ventilation, 0.49 in pharmacy, 0.17 in supermarket, 0.41 in post office, and 0.81 in bank (restaurant not modelled as lockdown implied restaurant closure; 95% CIs not reported)  Asymptomatic reproduction number after lockdown and with mechanical ventilation, 0.22 in pharmacy, 0.12 in supermarket, 0.17 in post office, and 0.34 in bank (restaurant not modelled as lockdown implied restaurant closure; 95% CIs not reported)  **Relative transmissibility according to symptom status**  Not reported | Not assessed | Authors noted that estimated asymptomatic reproduction number in restaurant before lockdown and with natural ventilation reflected high occupancy (80 customers and 4 waiting staff) and long exposure period (1.5 hours per sitting); asymptomatic viral load estimates incorporated in modelling (1×10^9^  copies per mL) might be more representative of presymptomatic/symptomatic viral load, and asymptomatic and symptomatic infections differed only in terms of respiratory and physical activity levels modelled; article combined estimation under assumed conditions and prediction of impact on epidemic spread of asymptomatic transmission in various indoor commercial environments |

Studies listed in chronological order; grey shading indicates consideration of evidence from outside mainland China
CI confidence interval; COVID-19 coronavirus disease 2019; CrI credible interval; MCMC Markov chain Monte Carlo; SARS-CoV severe acute respiratory syndrome coronavirus; SARS-CoV-2 severe acute respiratory syndrome coronavirus 2; SEIR susceptible–exposed–infected–recovered

## **Appendix H: GRADE tables**

**Table H.1** GRADE profile for absolute transmissibility of presymptomatic infections – cluster/outbreak studies

| **Quality assessment** | | | | | | | **Transmission demonstrated** | **Attack rate^b^** | **Quality** |
| --- | --- | --- | --- | --- | --- | --- | --- | --- | --- |
| **Number of studies** | **Design** | **Risk of bias^a^** | **Inconsistency** | **Indirectness** | **Imprecision** | **Other considerations** |  |  |  |
| **Contacts’ exposure period relative to index case acquiring infection: < 7 days** | | | | | | | | | |
| 1 (Chan 2020)^[10]^ | Observational studies | Serious^c^ | No serious inconsistency | No serious indirectness | Very serious^d^ | None | Uncertain | Not calculable | Very low |
| 1 (Kang 2020)^[25]^ | Observational studies | Very serious^e^ | No serious inconsistency | No serious indirectness | Very serious^d^ | None | Uncertain | Not calculable | Very low |
| 1 (Zhang 2020c)^[57]^ | Observational studies | Serious^c^ | No serious inconsistency | Serious^f^ | Very serious^d^ | None | Uncertain | Not calculable | Very low |
| 1 (Li 2020a)^[27]^ | Observational studies | Very serious^g^ | No serious inconsistency | No serious indirectness | Very serious^d^ | None | Uncertain | Not calculable | Very low |
| 1 (Xiao 2020)^[49]^ | Observational studies | Very serious^h^ | No serious inconsistency | No serious indirectness | Very serious^d^ | Yes^i^ | Uncertain | Not calculable | Very low |
| 1 (Song 2020)^[43]^ | Observational studies | Serious^c^ | No serious inconsistency | No serious indirectness | Very serious^d^ | None | Uncertain | Not calculable | Very low |
| 1 (Gao 2020b)^[15]^ | Observational studies | Very serious^j^ | No serious inconsistency | No serious indirectness | Very serious^d^ | None | Uncertain | Not calculable | Very low |
| 1 (Rothe 2020;^[41]^ Kupferschmidt 2020;^[26]^ Bohmer 2020)^[7]^ | Observational studies | Serious^c^ | No serious inconsistency | Serious^f^ | Very serious^d^ | None | Uncertain | Not calculable | Very low |
| 1 (Li 2020b)^[28]^ | Observational studies | Serious^c^ | No serious inconsistency | No serious indirectness | Very serious^d^ | None | Uncertain | Not calculable | Very low |
| 1 (Hijnen 2020)^[21]^ | Observational studies | No serious risk of bias | No serious inconsistency | No serious indirectness | Very serious^k^ | None | Yes | At least 85% (11/13; 95% CI 55% to 98%) | Very low |
| 1 (Wei 2020^[46]^ [‘cluster C’]) | Observational studies | No serious risk of bias | No serious inconsistency | No serious indirectness | Very serious^d^ | None | Uncertain | Not calculable | Very low |
| 1 (Wei 2020^[46]^ [‘cluster F’]) | Observational studies | No serious risk of bias | No serious inconsistency | No serious indirectness | Very serious^d^ | None | Uncertain | Not calculable | Very low |
| 1 (Wei 2020^[46]^ [‘cluster G’]) | Observational studies | No serious risk of bias | No serious inconsistency | No serious indirectness | Very serious^d^ | None | Uncertain | Not calculable | Very low |
| 1 (Baettig 2020)^[5]^ | Observational studies | Serious^l^ | No serious inconsistency | No serious indirectness | Serious^m^ | None | Uncertain | 2% (1/55; 95% CI 0.05% to 10%) | Very low |
| **Contacts’ exposure period relative to index case acquiring infection: 7 to 10 days** | | | | | | | | | |
| 1 (Wu 2020;^[47]^ Yang 2020;^[50]^ Liu 2020c)^[32]^ | Observational studies | Very serious^n^ | No serious inconsistency | No serious indirectness | Very serious^d^ | Yes^i^ | Uncertain | Not calculable | Very low |
| 1 (Wei 2020^[46]^ [‘cluster B’]) | Observational studies | No serious risk of bias | No serious inconsistency | No serious indirectness | Very serious^d^ | None | Uncertain | Not calculable | Very low |
| **Contacts’ exposure period relative to index case acquiring infection: not calculable, but < 10 days** | | | | | | | | | |
| 1 (Wei 2020^[46]^ [‘cluster E’]) | Observational studies | No serious risk of bias | No serious inconsistency | No serious indirectness | Very serious^d^ | None | Uncertain | Not calculable | Very low |
| **Contacts’ exposure period relative to index case acquiring infection: not calculable, but < 13 days** | | | | | | | | | |
| 1 (Huang 2020a)^[23]^ | Observational studies | No serious risk of bias | No serious inconsistency | No serious indirectness | Serious^m^ | None | Yes | 40% (7/22; 95% CI 14% to 55%) | Very low |
| **Contacts’ exposure period relative to index case acquiring infection: not calculable, but < 14 days** | | | | | | | | | |
| 1 (Wei 2020^[46]^ [‘cluster D’]) | Observational studies | No serious risk of bias | No serious inconsistency | No serious indirectness | Very serious^d^ | None | Uncertain | Not calculable | Very low |
| **Contacts’ exposure period relative to index case acquiring infection: not calculable** | | | | | | | | | |
| 1 (Cai 2020)^[9]^ | Observational studies | Serious^c^ | No serious inconsistency | No serious indirectness | Very serious^d^ | None | Uncertain | Not calculable | Very low |
| 1 (He 2020b)^[20]^ | Observational studies | Very serious^o^ | No serious inconsistency | No serious indirectness | Very serious^d^ | None | Uncertain | Not calculable | Very low |
| 1 (Li 2020d)^[30]^ | Observational studies | Very serious^e^ | No serious inconsistency | No serious indirectness | Very serious^d^ | None | Uncertain | Not calculable | Very low |
| 1 (Tong 2020)^[44]^ | Observational studies | Serious^c^ | No serious inconsistency | No serious indirectness | Very serious^d^ | None | Uncertain | Not calculable | Very low |
| 1 (Xia 2020)^[48]^ | Observational studies | Very serious^o^ | No serious inconsistency | No serious indirectness | Very serious^d^ | None | Uncertain | Not calculable | Very low |
| 1 (Yu 2020)^[54]^ | Observational studies | Serious^c^ | No serious inconsistency | No serious indirectness | Very serious^d^ | None | Yes | Not calculable | Very low |
| 1 (Guan 2020)^[17]^ | Observational studies | Serious^c^ | No serious inconsistency | No serious indirectness | Very serious^d^ | None | Uncertain | Not calculable | Very low |
| 1 (Yong 2020;^[53]^ Pung 2020^[36]^ [‘cluster C’]; Wei 2020^[46]^ [‘cluster A’]) | Observational studies | No serious risk of bias | No serious inconsistency | No serious indirectness | Very serious^d^ | None | Yes | Not calculable | Very low |
| 1 (Zhao 2020)^[58]^ | Observational studies | Very serious^p^ | No serious inconsistency | No serious indirectness | Very serious^d^ | Yes^i^ | Uncertain | Not calculable | Very low |
| 1 (Huang 2020b)^[24]^ | Observational studies | Very serious^q^ | No serious inconsistency | No serious indirectness | Very serious^d^ | None | Uncertain | Not calculable | Very low |
| 1 (Bae 2020)^[4]^ | Observational studies | Very serious^r^ | No serious inconsistency | No serious indirectness | Very serious^k^ | None | No | 0% (0/11; 95% CI 0% to 28%) | Very low |
| 1 (Liu 2020b)^[31]^ | Observational studies | Serious^c^ | No serious inconsistency | No serious indirectness | Very serious^k^ | None | Yes | 100% (1/1; 95% CI 3% to 100%) | Very low |
| 1 (Qiu 2020b)^[39]^ | Observational studies | Very serious^s^ | No serious inconsistency | No serious indirectness | Very serious^d^ | Yes^i^ | Uncertain | Not calculable | Very low |
| 1 (Ye 2020)^[51]^ | Observational studies | Very serious^j^ | No serious inconsistency | No serious indirectness | Very serious^d^ | None | Yes | Not calculable | Very low |
| 1 (Lu 2020a)^[33]^ | Observational studies | Serious^t^ | No serious inconsistency | No serious indirectness | Very serious^d^ | None | Yes | Not calculable | Very low |
| 1 (Hamner 2020)^[18]^ | Observational studies | Very serious^r^ | No serious inconsistency | No serious indirectness | Very serious^m^ | None | Uncertain | 5% (1/21; 95% CI 0.1% to 24%) | Very low |
| 1 (Ravaioli 2020)^[40]^ | Observational studies | Very serious^u^ | No serious inconsistency | No serious indirectness | Very serious^m^ | Yes^v^ | No | 0% (0/28; 95% CI 0% to 12%) | Very low |

CI confidence interval; HIS Healthcare Infection Society; PPE personal protective equipment

^a^ Risk of bias refers to limitations in design, analysis and reporting of studies not covered by other quality domains
^b^ Attack rates and CIs calculated by HIS team
^c^ Quality of evidence downgraded by 1 level because of lack of clarity regarding complete inclusion
^d^ Quality of evidence downgraded by 2 levels because no CIs or other measures of precision reported
^e^ Quality of evidence downgraded by 2 levels because of lack of clarity regarding consecutive inclusion and complete inclusion
^f^ Quality of evidence downgraded by 1 level because exposure was possibly pauci-symptomatic rather than presymptomatic
^g^ Quality of evidence downgraded by 2 levels because of lack of clarity regarding complete inclusion and reporting of presenting site(s)/clinic(s) demographic information
^h^ Quality of evidence downgraded by 2 levels because of lack of clarity regarding complete inclusion, reporting of demographics, and reporting of outcomes and follow up
^i^ One or more articles published in Chinese and incomplete translation might account for some lack of clarity in reporting
^j^ Quality of evidence downgraded by 2 levels because of lack of clarity regarding measurement in standard, reliable way, and complete inclusion
^k^ Quality of evidence downgraded by 2 levels because number of contacts investigated is below threshold of 18 needed to ensure 95% CI is narrower than 50 percentage points whatever the true attack rate
^l^ Quality of evidence downgraded by 1 level because of lack of clarity regarding inclusion criteria
^m^ Quality of evidence downgraded by 1 level because number of contacts investigated is below threshold of 68 needed to ensure 95% CI is narrower than 25 percentage points whatever the true attack rate
^n^ Quality of evidence downgraded by 2 levels because of lack of clarity regarding inclusion criteria, complete inclusion, reporting of demographics, and reporting of outcomes and follow up
^o^ Quality of evidence downgraded by 2 levels because of lack of clarity regarding measurement in standard, reliable way, valid methods used to identify condition, consecutive inclusion, and complete inclusion
^p^ Quality of evidence downgraded by 2 levels because of lack of clarity regarding measurement in standard, reliable way, complete inclusion, and reporting of demographics
^q^ Quality of evidence downgraded by 2 levels because of lack of clarity regarding complete inclusion and reporting of demographics
^r^ Quality of evidence downgraded by 2 levels because of lack of clarity regarding measurement in standard, reliable way, valid methods used to identify condition, and reporting of demographics
^s^ Quality of evidence downgraded by 2 levels because of lack of clarity regarding inclusion criteria, measurement in standard, reliable way, and complete inclusion
^t^ Quality of evidence downgraded by 1 level because of lack of clarity regarding reporting of demographics
^u^ Quality of evidence downgraded by 2 levels because of lack of clarity regarding reporting of demographics, reporting of outcomes and follow up, and reporting of presenting site(s)/clinic(s) demographic information
^v^ PPE in use

**Table H.2** GRADE profile for absolute transmissibility of asymptomatic infections – cluster/outbreak studies

| **Quality assessment** | | | | | | | **Transmission demonstrated** | **Attack rate^b^** | **Quality** |
| --- | --- | --- | --- | --- | --- | --- | --- | --- | --- |
| **Number of studies** | **Design** | **Risk of bias^a^** | **Inconsistency** | **Indirectness** | **Imprecision** | **Other considerations** |  |  |  |
| **Contacts’ exposure period relative to index case acquiring infection: < 7 days** | | | | | | | | | |
| 1 (Hu 2020)^[22]^ | Observational studies | No serious risk of bias | No serious inconsistency | Serious^c^ | Very serious^d^ | None | Yes | 100% (3/3; 95% CI 29% to 100%) | Very low |
| **Contacts’ exposure period relative to index case acquiring infection: 11 to 14 days** | | | | | | | | | |
| 1 (Scott 2020)^[42]^ | Observational studies | Very serious^e^ | No serious inconsistency | Serious^f^ | Very serious^d^ | Yes^g^ | No | 0% (0/10; 95% CI 0% to 31%) | Very low |
| **Contacts’ exposure period relative to index case acquiring infection: not calculable** | | | | | | | | | |
| 1 (Bai 2020b)^[6]^ | Observational studies | Serious^h^ | No serious inconsistency | No serious indirectness | Very serious^i^ | None | Uncertain | Not calculable | Very low |
| 1 (Gao 2020a)^[14]^ | Observational studies | No serious risk of bias | No serious inconsistency | Serious^j^ | No serious imprecision | Yes^k^ | No | 0% (0/455; 95% CI 0% to 0.8%) | Very low |
| 1 (Zhang 2020a)^[55]^ | Observational studies | Serious^h^ | No serious inconsistency | Serious^l^ | Very serious^i^ | None | Uncertain | Not calculable | Very low |
| 1 (Lu 2020b)^[34]^ | Observational studies | Very serious^m^ | No serious inconsistency | Serious^l^ | Very serious^i^ | None | Uncertain | Not calculable | Very low |
| 1 (Qiu 2020a)^[38]^ | Observational studies | Very serious^n^ | No serious inconsistency | No serious indirectness | Very serious^i^ | None | Uncertain | Not calculable | Very low |

CI confidence interval; COVID-19 coronavirus disease 2019; CT computerised tomography; HIS Healthcare Infection Society; PPE personal protective equipment

^a^ Risk of bias refers to limitations in design, analysis and reporting of studies not covered by other quality domains
^b^ Attack rates and CIs calculated by HIS team
^c^ Quality of evidence downgraded by 1 level because ‘asymptomatic’ index case had typical signs of viral infection on CT scan of the chest
^d^ Quality of evidence downgraded by 2 levels because number of contacts investigated is below threshold of 18 needed to ensure 95% CI is narrower than 50 percentage points whatever the true attack rate
^e^ Quality of evidence downgraded by 2 levels because of lack of clarity regarding complete inclusion and reporting of demographics
^f^ Quality of evidence downgraded by 1 level because ‘asymptomatic’ index case had a pre-existing cough
^g^ Some PPE in use
^h^ Quality of evidence downgraded by 1 level because of lack of clarity regarding complete inclusion
^i^ Quality of evidence downgraded by 2 levels because no CIs or other measures of precision reported
^j^ Quality of evidence downgraded by 1 level because ‘asymptomatic’ index case had shortness of breath associated with underlying health condition (and non-COVID-19 abnormalities on CT scan of the chest)
^k^ PPE in use
^l^ Quality of evidence downgraded by 1 level because ‘asymptomatic’ index case had ground-glass appearance on CT scan of the chest
^m^ Quality of evidence downgraded by 2 levels because of lack of clarity regarding inclusion criteria, measurement in standard, reliable way, valid methods used to identify condition, complete inclusion, and reporting of demographics
^n^ Quality of evidence downgraded by 2 levels because of lack of clarity regarding measurement in standard, reliable way, reporting of demographics, reporting of clinical information (e.g. uncertain whether CT scan of the chest performed), reporting of outcomes and follow up, and reporting of presenting site(s)/clinic(s) demographic information

**Table H.3** GRADE profile for absolute transmissibility of indeterminate (presymptomatic or asymptomatic) infections – cluster/outbreak studies

| **Quality assessment** | | | | | | | **Transmission demonstrated** | **Attack rate^b^** | **Quality** |
| --- | --- | --- | --- | --- | --- | --- | --- | --- | --- |
| **Number of studies** | **Design** | **Risk of bias^a^** | **Inconsistency** | **Indirectness** | **Imprecision** | **Other considerations** |  |  |  |
| **Contacts’ exposure period relative to index case acquiring infection: < 7 days** | | | | | | | | | |
| 1 (Qian 2020)^[37]^ | Observational studies | Serious^c^ | No serious inconsistency | No serious indirectness | Very serious^d^ | Yes^e^ | Uncertain | 75% (3/4; 95% CI 19% to 99%), or 80% (4/5; 95% CI 28% to 99%) if second index case counted as exposed | Very low |
| **Contacts’ exposure period relative to index case acquiring infection: not calculable** | | | | | | | | | |
| 1 (Pung 2020^[36]^ [‘cluster B’]) | Observational studies | Serious^f^ | No serious inconsistency | No serious indirectness | Very serious^g^ | Yes^h^ | Uncertain | Not calculable | Very low |

CI confidence interval; HIS Healthcare Infection Society

^a^ Risk of bias refers to limitations in design, analysis and reporting of studies not covered by other quality domains
^b^ Attack rates and CIs calculated by HIS team
^c^ Quality of evidence downgraded by 1 level because of lack of clarity regarding complete inclusion
^d^ Quality of evidence downgraded by 2 levels because number of contacts investigated is below threshold of 18 needed to ensure 95% CI is narrower than 50 percentage points whatever the true attack rate
^e^ Two index cases with common exposure, but only 1 developed symptoms; potentially presymptomatic or asymptomatic transmission
^f^ Quality of evidence downgraded by 1 level because of lack of clarity regarding reporting of demographics
^d^ Quality of evidence downgraded by 2 levels because no CIs or other measures of precision reported ^h^ Index case assumed to be unidentified conference attendee; potentially presymptomatic or asymptomatic transmission

**Table H.4** GRADE profile for relative transmissibility according to symptom status (fewer symptoms versus more symptoms) – nested case–control studies^a^

| **Quality assessment** | | | | | | | **Number of people** | | **Effect** | | **Quality** |
| --- | --- | --- | --- | --- | --- | --- | --- | --- | --- | --- | --- |
| **Number of studies** | **Design** | **Risk of bias^b^** | **Inconsistency** | **Indirectness** | **Imprecision** | **Other considerations** | **Fewer symptoms** | **More symptoms** | **Relative^c^** | **Absolute** |  |
| **Presymptomatic exposure versus symptomatic exposure** | | | | | | | | | | | |
| 1 (Cheng 2020)^[12]^ | Observational studies | Very serious^d^ | No serious inconsistency | No serious indirectness | Very serious^e^ | None | Cases: 2 Controls: 297 | Cases:20 Controls: 2351 | OR 0.79 (95% CI 0.18 to 3.40) | Not applicable | Very low |
| 1 (Park 2020)^[35]^ | Observational studies | Serious^f^ | No serious inconsistency | No serious indirectness | Very serious^e^ | None | Cases: 0 Controls: 11 | Cases: 34 Controls: 176 | OR 0.22 (95% CI 0.01 to 3.86) | Not applicable | Very low |
| **Asymptomatic exposure versus symptomatic exposure, including super-spreader** | | | | | | | | | | | |
| 1 (Chen 2020;^[11]^ He 2020a;^[19]^ Yin 2020)^[52]^ | Observational studies | Very serious^d^ | No serious inconsistency | No serious indirectness | Very serious^e^ | None^g^ | Cases: 6 Controls: 140 | Cases: 126 Controls: 1875 | OR 0.64 (95% CI 0.28 to 1.47) | Not applicable | Very low |
| **Asymptomatic exposure versus symptomatic exposure, excluding super-spreader** | | | | | | | | | | | |
| 1 (Chen 2020;^[11]^ He 2020a;^[19]^ Yin 2020)^[52]^ | Observational studies | Very serious^d^ | No serious inconsistency | No serious indirectness | Very serious^e^ | None^g^ | Cases: 6 Controls: 140 | Cases: 94 Controls: 1810 | OR 0.83 (95% CI 0.36 to 1.92) | Not applicable | Very low |
| **Asymptomatic exposure versus symptomatic exposure** | | | | | | | | | | | |
| 1 (Cheng 2020)^[12]^ | Observational studies | Very serious^d^ | No serious inconsistency | No serious indirectness | Very serious^e^ | None | Cases: 0 Controls: 91 | Cases: 20 Controls: 2351 | OR 0.63 (95% CI 0.04 to 10.44) | Not applicable | Very low |
| 1 (Park 2020)^[35]^ | Observational studies | Serious^f^ | No serious inconsistency | No serious indirectness | Very serious^e^ | None | Cases: 0 Controls: 4 | Cases: 34 Controls: 176 | OR 0.57 (95% CI 0.03 to 10.80) | Not applicable | Very low |
| **Asymptomatic exposure versus presymptomatic exposure** | | | | | | | | | | | |
| 1 (Zhang 2020b)^[56]^ | Observational studies | Serious^f^ | No serious inconsistency | No serious indirectness | Very serious^e^ | None | Cases: 1 Controls: 124 | Cases: 11 Controls: 233 | OR 0.17 (95% CI 0.02 to 1.34) | Not applicable | Very low |

CI confidence interval; HIS Healthcare Infection Society; OR odds ratio; SARS-CoV-2 severe acute respiratory syndrome coronavirus 2

^a^ Fewer/more symptoms refers to symptom status in source individuals during exposure of contacts; cases/controls refers to contacts who tested positive/negative, respectively, for SARS-CoV-2
^b^ Risk of bias refers to limitations in design, analysis and reporting of studies not covered by other quality domains
^c^ ORs and CIs calculated by HIS team
^d^ Quality of evidence downgraded by 2 levels because not possible to tell whether exposure was measured accurately, and confounding factors not accounted for in design/analysis of study
^e^ Quality of evidence downgraded by 2 levels because the 95% CI crosses both lower (0.8) and upper (1.25) default thresholds for imprecision of dichotomous outcomes
^f^ Quality of evidence downgraded by 1 level because confounding factors not accounted for in design/analysis of study
^g^ One or more articles published in Chinese and incomplete translation might account for some lack of clarity in reporting

**Table H.5** GRADE profile for relative transmissibility according to symptom status (fewer symptoms versus more symptoms) – mathematical modelling studies^a^

| **Quality assessment** | | | | | | | **Number of people** | | **Effect** | | **Quality** |
| --- | --- | --- | --- | --- | --- | --- | --- | --- | --- | --- | --- |
| **Number of studies** | **Design** | **Risk of bias** | **Inconsistency** | **Indirectness** | **Imprecision** | **Other considerations** | **Fewer symptoms** | **More symptoms** | **Relative** | **Absolute** |  |
| **Infectious but asymptomatic versus infectious with symptoms** | | | | | | | | | | | |
| 1 (Wan 2020)^[45]^ | Modelling studies | Not assessed | No serious inconsistency | Serious^b^ | Very serious | None | Not applicable | Not applicable | RR 0.81 (95% CrI not reported) | Not applicable | Not applicable |
| **Asymptomatic or with mild symptoms versus infected with severe symptoms** | | | | | | | | | | | |
| 1 (Gatto 2020)^[16]^ | Modelling studies | Not assessed | No serious inconsistency | Very serious^c^ | Very serious | None | Not applicable | Not applicable | Not calculable^d^ | Not applicable | Not applicable |
| **Asymptomatic or with mild symptoms versus presymptomatic** | | | | | | | | | | | |
| 1 (Gatto 2020)^[16]^ | Modelling studies | Not assessed | No serious inconsistency | Very serious^c^ | No serious imprecision | None | Not applicable | Not applicable | RR 0.033 (95% CrI 0.027 to 0.036) | Not applicable | Not applicable |
| **Undocumented infections versus documented infections without containment measures (10 Jan to 23 Jan 2020)** | | | | | | | | | | | |
| 1 (Li 2020c)^[29]^ | Modelling studies | Not assessed | No serious inconsistency | Very serious^e^ | No serious imprecision | None | Not applicable | Not applicable | RR 0.55 (95% CrI 0.49 to 0.60) | Not applicable | Not applicable |
| **Undocumented infections versus documented infections with containment measures (24 Jan to 3 Feb 2020)** | | | | | | | | | | | |
| 1 (Li 2020c)^[29]^ | Modelling studies | Not assessed | No serious inconsistency | Very serious^e^ | No serious imprecision | None | Not applicable | Not applicable | RR 0.47 (95% CrI 0.36 to 0.64) | Not applicable | Not applicable |
| **Undocumented infections versus documented infections with containment measures (24 Jan to 8 Feb 2020)** | | | | | | | | | | | |
| 1 (Li 2020c)^[29]^ | Modelling studies | Not assessed | No serious inconsistency | Very serious^e^ | No serious imprecision | None | Not applicable | Not applicable | RR 0.42 (95% CrI 0.34 to 0.61) | Not applicable | Not applicable |
| **Presymptomatic reproduction number as percentage of total reproduction number** | | | | | | | | | | | |
| 1 (Ferretti 2020)^[13]^ | Modelling studies | Not assessed | No serious inconsistency | No serious indirectness | Not assessed | None | Not applicable | Not applicable | 47% (95% CrI 11% to 58%)^f^ | Not applicable | Not applicable |
| **Asymptomatic reproduction number as percentage of total reproduction number** | | | | | | | | | | | |
| 1 (Ferretti 2020)^[13]^ | Modelling studies | Not assessed | No serious inconsistency | No serious indirectness | Not assessed | None | Not applicable | Not applicable | 6% (95% CrI 0% to 57%)^f^ | Not applicable | Not applicable |
| **Symptomatic reproduction number as percentage of total reproduction number** | | | | | | | | | | | |
| 1 (Ferretti 2020)^[13]^ | Modelling studies | Not assessed | No serious inconsistency | No serious indirectness | Not assessed | None | Not applicable | Not applicable | 28% (95% CrI 9% to 49%)^f^ | Not applicable | Not applicable |

CrI credible interval; RR relative risk

^a^ Fewer/more symptoms refers to symptom status in source individuals during exposure of contacts
^b^ Quality of evidence downgraded by 1 level because mathematical modelling studies provide indirect estimates of transmission risks compared to epidemiological studies
^c^ Quality of evidence downgraded by 2 levels because mathematical modelling studies provide indirect estimates of transmission risks compared to epidemiological studies, and asymptomatic infection grouped together with infection with mild symptoms
^d^ Study reports relative effect for more symptoms versus fewer symptoms, i.e. infected with severe symptoms versus asymptomatic or with mild symptoms, RR 1.03 (95% CrI 0.79 to 1.38)
^e^ Quality of evidence downgraded by 2 levels because mathematical modelling studies provide indirect estimates of transmission risks compared to epidemiological studies, and symptom statuses investigated not wholly aligned with those of interest (presymptomatic or asymptomatic); undocumented infected defined as lacking symptoms severe enough to be confirmed/observed; documented infected defined as symptoms severe enough to be confirmed/observed
^f^ Percentages for presymptomatic, asymptomatic and symptomatic transmission do not add to 100%; remainder corresponds to environmental transmission (defined as occurring through contamination not accounted for by identifying close contacts)

**Table H.6** GRADE profile for relative transmissibility of asymptomatic infections according to ventilation characteristics in indoor commercial environments – mathematical modelling studies

| **Quality assessment** | | | | | | | **Number of people** | | **Effect** | | **Quality** |
| --- | --- | --- | --- | --- | --- | --- | --- | --- | --- | --- | --- |
| **Number of studies** | **Design** | **Risk of bias** | **Inconsistency** | **Indirectness** | **Imprecision** | **Other considerations** | **Mechanical ventilation** | **Natural ventilation** | **Relative** | **Absolute** |  |
| **Asymptomatic reproduction number before lockdown, mechanical ventilation versus natural ventilation** | | | | | | | | | | | |
| 1 (Buonanno 2020)^[8]^ | Modelling studies | Not assessed | No serious inconsistency | Very serious^a^ | Very serious^b^ | None | Not applicable | Not applicable | Not reported^c^ | Not applicable | Not applicable |
| **Asymptomatic reproduction number during lockdown, mechanical ventilation versus natural ventilation** | | | | | | | | | | | |
| 1 (Buonanno 2020)^[8]^ | Modelling studies | Not assessed | No serious inconsistency | Very serious^a^ | Very serious^b^ | None | Not applicable | Not applicable | Not reported^d^ | Not applicable | Not applicable |

CI confidence interval

^a^ Quality of evidence downgraded by 2 levels because mathematical modelling studies provide indirect estimates of transmission risks compared to epidemiological studies, asymptomatic viral load estimates incorporated in modelling (1×10^9^ copies per mL) might be more representative of presymptomatic/symptomatic viral load, and asymptomatic/symptomatic infections differed only in terms of respiratory/physical activity levels modelled
^b^ Quality of evidence downgraded by 2 levels because no CIs or other measures of precision reported
^c^ Study reports reproduction numbers for asymptomatic transmission before lockdown and with mechanical/natural ventilation in 5 indoor commercial environments: supermarket, 2.19 with natural ventilation; bank, 3.52 with natural ventilation; post office, 3.64 with natural ventilation; pharmacy, 3.70 with natural ventilation; restaurant, 5.35 with mechanical ventilation versus 47.3 with natural ventilation; reproduction numbers with mechanical ventilation reported only for restaurant, other environments ranged from 1.16 to 1.30; no CIs or other measures of precision reported
^d^ Study reports reproduction numbers for asymptomatic transmission during lockdown and with mechanical/natural ventilation in 4 indoor commercial environments: supermarket, 0.12 with mechanical ventilation versus 0.17 with natural ventilation; post office, 0.17 with mechanical ventilation versus 0.41 with natural ventilation; pharmacy, 0.22 with mechanical ventilation versus 0.49 with natural ventilation; bank, 0.34 with mechanical ventilation versus 0.81 with natural ventilation; restaurants required to close during lockdown and additional voluntary measures included fewer staff on duty, customers queueing outside, and ventilation increased by keeping external doors open; no CIs or other measures of precision reported

## **Appendix I: Consultation**

The stakeholder consultation on the draft guideline was active from 4 to 18 May 2021.

**Table I.1** Consultation comments and responses

| **Stakeholder** | **Section** | **Comment** | **Working Party’s response** |
| --- | --- | --- | --- |
| Anonymised at the stakeholder’s request | 1 | *Be aware that the future importance of transmission of SARS-CoV-2 by people carrying the virus without symptoms might depend on the:*   - *nature of further waves or outbreaks of COVID-19* - *emergence and circulation of SARS-CoV-2 variants of concern*   Does it need to state explicitly that new variants of concern may be more or less transmissible, thereby changing implications of previous recommendations | The recommendation has been rephrased to state that the future transmissibility of SARS-CoV-2 from people carrying the virus without symptoms might depend on the factors listed in the bullets, including the emergence and circulation of variants of concern as noted in the comment |
| Anonymised at the stakeholder’s request | 7 | Would be helpful to have a section on effect of seasonality on transmission | The Working Party recognises that this is an area of interest, but the suggestion does not fit with the scope of the current guidance, which focuses on transmission according to the symptom status of people who have acquired SARS-CoV-2 and the evidence identified in relation to this. The Working Party has added a note to the rationale for the research recommendations stating that this might be explored as part of future research |
| Anonymised at the stakeholder’s request | 7 | Would be helpful to briefly discuss the difference between droplet and airborne transmission | The Working Party has examined routes of transmission (including droplet and airborne transmission) in the first article in the pair of guidance documents.^[3]^ These topics are, therefore, outside the scope of this second guidance article |
| Anonymised at the stakeholder’s request | 7 | Numerical studies of the transport and distribution of particles or droplets formed during normal breathing, sneezing or coughing in a room lead to the following conclusions: in a normal breathing process, particles or droplets can only be transported over short distances; when sneezing or coughing, particles are transported over long distances.  The social distance recommended by WHO of 2 m is performed for simple breathing for all cases (without ventilation and with ventilation). However, when coughing or sneezing, this distance is clearly not enough and it needs at least 5 m social distance in order not to get into the zone of exposure to these particles.  Issakhov, A., Zhandaulet, Y., Omarova, P. et al. A numerical assessment of social distancing of preventing airborne transmission of COVID-19 during different breathing and coughing processes. Sci Rep 11, 9412 (2021). https://doi.org/10.1038/s41598-021-88645-2 | The Working Party has examined routes of transmission (including droplet and airborne transmission) in the first article in the pair of guidance documents.^[3]^ These topics are, therefore, outside the scope of this second guidance article |
| Anonymised at the stakeholder’s request | 7 | This paper reports that the outdoor risk of transmission is orders of magnitude less than the indoor risk and that it can become comparable only for extremely specific meteorological and topographical situations  Rowe BR, Canosa A, Drouffe JM, Mitchell JBA. Simple quantitative assessment of the outdoor versus indoor airborne transmission of viruses and COVID-19. Environ Res. 2021 Apr 16;198:111189. doi: 10.1016/j.envres.2021.111189. Epub ahead of print. PMID: 33872644; PMCID: PMC8051020. | The Working Party recognises that this is an area of interest, but the suggestion does not fit with the scope of the current guidance, which focuses on transmission according to the symptom status of people who have acquired SARS-CoV-2 and the evidence identified in relation to this. The Working Party has added a note to the rationale for the research recommendations stating that this might be explored as part of future research |
| Anonymised at the stakeholder’s request | 7 | Reference demonstrating that aerosols may, be responsible for so-called super-spreader events (SSE's).Indirect evidence points to a correlation between ventilation and the transmission and spread of SARS-Cov-2, supporting ventilation as an important factor in preventing airborne transmission.  van der Valk JPM, In 't Veen JCCM. SARS-Cov-2: The relevance and Prevention of Aerosol Transmission. J Occup Environ Med. 2021 Mar 16. doi: 10.1097/JOM.0000000000002193. | The Working Party has examined routes of transmission (including droplet and airborne transmission) in the first article in the pair of guidance documents.^[3]^ These topics are, therefore, outside the scope of this second guidance article |
| Anonymised at the stakeholder’s request | 7 | Some key references on transmission missing  Lewis D. Superspreading drives the COVID pandemic—and could help to tame it. Nature 2021; 590: 544–46.  Bulfone TC, Malekinejad M, Rutherford GW, Razani N. Outdoor transmission of SARS-CoV-2 and other respiratory viruses: a systematic review. J Infect Dis 2021; 223: 550–61  Van Doremalen N, Bushmaker T, Morris DH, et al. Aerosol and surface stability of SARS-CoV-2 as compared with SARS-CoV-1. New Engl J Med 2020; 382: 1564–67.  Lednicky JA, Lauzard M, Fan ZH, et al. Viable SARS-CoV-2 in the air of a hospital room with COVID-19 patients. Int J Infect Dis 2020; 100: 476–82. | Thank you for sharing the references in this and other comments. Several of the articles were published after the literature searches for this guidance article were conducted and the Working Party has emphasised in the guidance article that it was not feasible to undertake a systematic update using the original search terms because of the unexpectedly large volume of evidence that has been published during the pandemic. Some of the articles published in 2020 (for example, Van Doremalen 2020 and Lednicky 2020) were included in the evidence reviewed for the first article in the pair of guidance documents, which examined routes of transmission (including droplet and airborne transmission).^[3]^ |
| Anonymised at the stakeholder’s request |  | Risk of transmission due to airborne SARS-CoV-2 particles from breathing, speaking, singing, coughing, and sneezing in indoor environments.  Schijven J, Vermeulen LC, Swart A, Meijer A, Duizer E, de Roda Husman AM. Quantitative Microbial Risk Assessment for Airborne Transmission of SARS-CoV-2 via Breathing, Speaking, Singing, Coughing, and Sneezing. Environ Health Perspect. 2021 Apr;129(4):47002. doi: 10.1289/EHP7886. Epub 2021 Apr 1. PMID: 33793301; PMCID: PMC8016178. | The Working Party has examined routes of transmission (including droplet and airborne transmission) in the first article in the pair of guidance documents.^[3]^ These topics are, therefore, outside the scope of this second guidance article |
| Anonymised at the stakeholder’s request |  | High temperature and high relative humidity reduce COVID-19 transmission and support the mechanical model of the effect of moisture in the COVID-19 airborne transmission.  Arias FJ, De Las Heras S. The mechanical effect of moisturization on airborne COVID-19 transmission and its potential use as control technique. Environ Res. 2021 Mar 13;197:110940. doi: 10.1016/j.envres.2021.110940. Epub ahead of print. PMID: 33726993; PMCID: PMC7955575. | The Working Party has examined routes of transmission (including droplet and airborne transmission) in the first article in the pair of guidance documents.^[3]^ These topics are, therefore, outside the scope of this second guidance article |

**References**

1. JHU. Coronavirus Resource Center: Johns Hopkins University & Medicine (JHU); 2020 [Available from: https://coronavirus.jhu.edu/; accessed 30 November 2021].

2. NICE. Developing NICE guidelines: the manual: National Institute for Health and Care Excellence (NICE); 2014 [Available from: https://www.nice.org.uk/process/pmg20/; accessed 30 November 2021].

3. Bak A, Mugglestone MA, Ratnaraja NV, Wilson JA, Loveday HP, Rivett L, et al. SARS-CoV-2 routes of transmission and recommendations for preventing acquisition: joint British Infection Association (BIA), Healthcare Infection Society (HIS), Infection Prevention Society (IPS) and Royal College of Pathologists (RCPath) guidance. Journal of Hospital Infection. 2021;114:79-103.

4. Bae JM. A Chinese case of coronavirus disease 2019 (COVID-19) did not show infectivity during the incubation period: Based on an epidemiological survey. Journal of Preventive Medicine and Public Health. 2020;53(2):67-9.

5. Baettig SJ, Parini A, Cardona I, Morand GB. Case series of coronavirus (SARS-CoV-2) in a military recruit school: clinical, sanitary and logistical implications. BMJ Mil Health. 2020;16:16.

6. Bai Y, Yao L, Wei T, Tian F, Jin DY, Chen L, et al. Presumed Asymptomatic Carrier Transmission of COVID-19. Jama. 2020;21:21.

7. Bohmer MM, Buchholz U, Corman VM, Hoch M, Katz K, Marosevic DV, et al. Investigation of a COVID-19 outbreak in Germany resulting from a single travel-associated primary case: a case series. The Lancet Infectious Diseases. 2020;15:15.

8. Buonanno G, Stabile L, Morawska L. Estimation of airborne viral emission: Quanta emission rate of SARS-CoV-2 for infection risk assessment. Environ Int. 2020;141:105794.

9. Cai J, Sun W, Huang J, Gamber M, Wu J, He G. Indirect Virus Transmission in Cluster of COVID-19 Cases, Wenzhou, China, 2020. Emerg Infect Dis. 2020;26(6):1343-5.

10. Chan JF, Yuan S, Kok KH, To KK, Chu H, Yang J, et al. A familial cluster of pneumonia associated with the 2019 novel coronavirus indicating person-to-person transmission: a study of a family cluster. Lancet. 2020;395(10223):514-23.

11. Chen Y, Wang AH, Yi B, Ding KQ, Wang HB, Wang JM, et al. [Epidemiological characteristics of infection in COVID-19 close contacts in Ningbo city]. Chung Hua Liu Hsing Ping Hsueh Tsa Chih. 2020;41(5):667-71.

12. Cheng HY, Jian SW, Liu DP, Ng TC, Huang WT, Lin HH, et al. Contact Tracing Assessment of COVID-19 Transmission Dynamics in Taiwan and Risk at Different Exposure Periods Before and After Symptom Onset. JAMA Intern Med. 2020;01:01.

13. Ferretti L, Wymant C, Kendall M, Zhao L, Nurtay A, Abeler-Dorner L, et al. Quantifying SARS-CoV-2 transmission suggests epidemic control with digital contact tracing. Science. 2020;368(6491):08.

14. Gao M, Yang L, Chen X, Deng Y, Yang S, Xu H, et al. A study on infectivity of asymptomatic SARS-CoV-2 carriers. Respir Med. 2020;169:106026.

15. Gao Y, Shi C, Chen Y, Shi P, Liu J, Xiao Y, et al. A cluster of the Corona Virus Disease 2019 caused by incubation period transmission in Wuxi, China. Journal of Infection. 2020;80(6):666-70.

16. Gatto M, Bertuzzo E, Mari L, Miccoli S, Carraro L, Casagrandi R, et al. Spread and dynamics of the COVID-19 epidemic in Italy: Effects of emergency containment measures. Proceedings of the National Academy of Sciences of the United States of America. 2020;117(19):10484-91.

17. Guan Q, Liu M, Zhuang YJ, Yuan Y, Wang SS, Li J, et al. [Epidemiological investigation of a family clustering of COVID-19]. Chung Hua Liu Hsing Ping Hsueh Tsa Chih. 2020;41(5):629-33.

18. Hamner L, Dubbel P, Capron I, Ross A, Jordan A, Lee J, et al. High SARS-CoV-2 Attack Rate Following Exposure at a Choir Practice - Skagit County, Washington, March 2020. MMWR Morb Mortal Wkly Rep. 2020;69(19):606-10.

19. He D, Zhao S, Lin Q, Zhuang Z, Cao P, Wang MH, et al. The relative transmissibility of asymptomatic COVID-19 infections among close contacts. International Journal of Infectious Diseases. 2020;94:145-7.

20. He X, Lau EHY, Wu P, Deng X, Wang J, Hao X, et al. Temporal dynamics in viral shedding and transmissibility of COVID-19. Nature Medicine. 2020;26(5):672-5.

21. Hijnen D, Marzano AV, Eyerich K, GeurtsvanKessel C, Gimenez-Arnau AM, Joly P, et al. SARS-CoV-2 Transmission from Presymptomatic Meeting Attendee, Germany. Emerg Infect Dis. 2020;26(8):11.

22. Hu Z, Song C, Xu C, Jin G, Chen Y, Xu X, et al. Clinical characteristics of 24 asymptomatic infections with COVID-19 screened among close contacts in Nanjing, China. Sci China Life Sci. 2020;63(5):706-11.

23. Huang L, Zhang X, Zhang X, Wei Z, Zhang L, Xu J, et al. Rapid asymptomatic transmission of COVID-19 during the incubation period demonstrating strong infectivity in a cluster of youngsters aged 16-23 years outside Wuhan and characteristics of young patients with COVID-19: A prospective contact-tracing study. Journal of Infection. 2020;80(6):e1-e13.

24. Huang R, Xia J, Chen Y, Shan C, Wu C. A family cluster of SARS-CoV-2 infection involving 11 patients in Nanjing, China. The Lancet Infectious Diseases. 2020;20(5):534-5.

25. Kang M, Jie Wu J, Ma W, He J, Lu J, Liu T, et al. Evidence and characteristics of human-to-human transmission of SARS-CoV-2. medRxiv preprint. 2020.

26. Kupferschmidt K. Study claiming new coronavirus can be transmitted by people without symptoms was flawed. Science. 2020.

27. Li C, Ji F, Wang L, Wang L, Hao J, Dai M, et al. Asymptomatic and Human-to-Human Transmission of SARS-CoV-2 in a 2-Family Cluster, Xuzhou, China. Emerg Infect Dis. 2020;26(7):31.

28. Li P, Fu JB, Li KF, Chen Y, Wang HL, Liu LJ, et al. Transmission of COVID-19 in the terminal stage of incubation period: a familial cluster. International Journal of Infectious Diseases. 2020;16:16.

29. Li R, Pei S, Chen B, Song Y, Zhang T, Yang W, et al. Substantial undocumented infection facilitates the rapid dissemination of novel coronavirus (SARS-CoV-2). Science. 2020;368(6490):489-93.

30. Li YK, Peng S, Li LQ, Wang Q, Ping W, Zhang N, et al. Clinical and Transmission Characteristics of Covid-19 - A Retrospective Study of 25 Cases from a Single Thoracic Surgery Department. Curr Med Sci. 2020;40(2):295-300.

31. Liu YC, Liao CH, Chang CF, Chou CC, Lin YR. A locally transmitted case of SARS-CoV-2 infection in Taiwan. New England Journal of Medicine. 2020;382 (11) (no pagination)(NEJMc2001573).

32. Liu YF, Li JM, Zhou PH, Liu J, Dong XC, Lyu J, et al. [Analysis on cluster cases of COVID-19 in Tianjin]. Chung Hua Liu Hsing Ping Hsueh Tsa Chih. 2020;41(5):653-6.

33. Lu J, Gu J, Li K, Xu C, Su W, Lai Z, et al. COVID-19 Outbreak Associated with Air Conditioning in Restaurant, Guangzhou, China, 2020. Emerg Infect Dis. 2020;26(7):1628-31.

34. Lu S, Lin J, Zhang Z, Xiao L, Jiang Z, Chen J, et al. Alert for non-respiratory symptoms of Coronavirus Disease 2019 (COVID-19) patients in epidemic period: A case report of familial cluster with three asymptomatic COVID-19 patients. Journal of Medical Virology. 2020.

35. Park SY, Kim YM, Yi S, Lee S, Na BJ, Kim CB, et al. Coronavirus Disease Outbreak in Call Center, South Korea. Emerg Infect Dis. 2020;26(8):23.

36. Pung R, Chiew CJ, Young BE, Chin S, Chen MIC, Clapham HE, et al. Investigation of three clusters of COVID-19 in Singapore: implications for surveillance and response measures. The Lancet. 2020;395(10229):1039-46.

37. Qian G, Yang N, Ma AHY, Wang L, Li G, Chen X, et al. A COVID-19 Transmission within a family cluster by presymptomatic infectors in China. Clin Infect Dis. 2020;23:23.

38. Qiu C, Deng Z, Xiao Q, Shu Y, Deng Y, Wang H, et al. Transmission and clinical characteristics of coronavirus disease 2019 in 104 outside-Wuhan patients, China. Journal of Medical Virology. 2020;05:05.

39. Qiu YY, Wang SQ, Wang XL, Lu WX, Qiao D, Li JB, et al. [Epidemiological analysis on a family cluster of COVID-19]. Chung Hua Liu Hsing Ping Hsueh Tsa Chih. 2020;41(4):494-7.

40. Ravaioli M, Comai G, Germinario G, Maroni L, La Manna G. Kidney transplantation with pre-symptomatic COVID-19 positive surgeon. American Journal of Transplantation. 2020;05:05.

41. Rothe C, Schunk M, Sothmann P, Bretzel G, Froeschl G, Wallrauch C, et al. Transmission of 2019-nCoV Infection from an Asymptomatic Contact in Germany. New England Journal of Medicine. 2020;382(10):970-1.

42. Scott SE, Zabel K, Collins J, Hobbs KC, Kretschmer MJ, Lach M, et al. First Mildly Ill, Non-Hospitalized Case of Coronavirus Disease 2019 (COVID-19) Without Viral Transmission in the United States - Maricopa County, Arizona, 2020. Clin Infect Dis. 2020;02:02.

43. Song R, Han B, Song M, Wang L, Conlon CP, Dong T, et al. Clinical and epidemiological features of COVID-19 family clusters in Beijing, China. Journal of Infection. 2020;23:23.

44. Tong ZD, Tang A, Li KF, Li P, Wang HL, Yi JP, et al. Potential Presymptomatic Transmission of SARS-CoV-2, Zhejiang Province, China, 2020. Emerg Infect Dis. 2020;26(5):1052-4.

45. Wan H, J-A. C, G-J. Y. Risk estimation and prediction by modeling the transmission of the novel coronavirus (COVID-19) in mainland China excluding Hubei province. medRxiv preprint. 2020.

46. Wei WE, Li Z, Chiew CJ, Yong SE, Toh MP, Lee VJ. Presymptomatic Transmission of SARS-CoV-2 - Singapore, January 23-March 16, 2020. MMWR Morb Mortal Wkly Rep. 2020;69(14):411-5.

47. Wu WS, Li YG, Wei ZF, Zhou PH, Lyu LK, Zhang GP, et al. [Investigation and analysis on characteristics of a cluster of COVID-19 associated with exposure in a department store in Tianjin]. Chung Hua Liu Hsing Ping Hsueh Tsa Chih. 2020;41(4):489-93.

48. Xia W, Liao J, Li C, Li Y, Qian X, Sun X, et al. Transmission of corona virus disease 2019 during the incubation period may lead to a quarantine loophole. medRxiv preprint. 2020.

49. Xiao WJ, Gao Q, Jin K, Gong XH, Han RB, Jiang CY, et al. [Investigation of an epidemic cluster caused by COVID-19 cases in incubation period in Shanghai]. Chung Hua Liu Hsing Ping Hsueh Tsa Chih. 2020;41(0):E033.

50. Yang HY, Xu J, Li Y, Liang X, Jin YF, Chen SY, et al. [The preliminary analysis on the characteristics of the cluster for the COVID-19]. Chung Hua Liu Hsing Ping Hsueh Tsa Chih. 2020;41(5):623-8.

51. Ye F, Xu S, Rong Z, Xu R, Liu X, Deng P, et al. Delivery of infection from asymptomatic carriers of COVID-19 in a familial cluster. International Journal of Infectious Diseases. 2020;94:133-8.

52. Yin G, Jin H. Comparison of Transmissibility of Coronavirus Between Symptomatic and Asymptomatic Patients: Reanalysis of the Ningbo COVID-19 Data. JMIR Public Health Surveill. 2020;6(2):e19464.

53. Yong SEF, Anderson DE, Wei WE, Pang J, Chia WN, Tan CW, et al. Connecting clusters of COVID-19: an epidemiological and serological investigation. The Lancet Infectious Diseases. 2020;20(7):809-15.

54. Yu P, Zhu J, Zhang Z, Han Y. A Familial Cluster of Infection Associated With the 2019 Novel Coronavirus Indicating Possible Person-to-Person Transmission During the Incubation Period. J Infect Dis. 2020;221(11):1757-61.

55. Zhang J, Tian S, Lou J, Chen Y. Familial cluster of COVID-19 infection from an asymptomatic. Crit Care. 2020;24(1):119.

56. Zhang W, Cheng W, Luo L, Ma Y, Xu C, Qin P, et al. Secondary Transmission of Coronavirus Disease from Presymptomatic Persons, China. Emerg Infect Dis. 2020;26(8):26.

57. Zhang XA, Fan H, Qi RZ, Zheng W, Zheng K, Gong JH, et al. Importing coronavirus disease 2019 (COVID-19) into China after international air travel. Travel Medicine and Infectious Disease. 2020;35 (no pagination)(101620).

58. Zhao H, Li BS, Xia Y, Zhou HL, Li TR, Zeng Y, et al. [Investigation of transmission chain of a cluster COVID-19 cases]. Chung Hua Liu Hsing Ping Hsueh Tsa Chih. 2020;41(0):E064.

59. Rivett L, Sridhar S, Sparkes D, Routledge M, Jones NK, Forrest S, et al. Screening of healthcare workers for SARS-CoV-2 highlights the role of asymptomatic carriage in COVID-19 transmission. elife. 2020;9:11.

60. To KKW, Hung IFN, Ip JD, Chu AWH, Chan WM, Tam AR, et al. COVID-19 re-infection by a phylogenetically distinct SARS-coronavirus-2 strain confirmed by whole genome sequencing. Clinical infectious diseases : an official publication of the Infectious Diseases Society of America. 2020.

61. Buitrago-Garcia D, Egli-Gany D, Counotte MJ, Hossmann S, Imeri H, Ipekci AM, et al. Occurrence and transmission potential of asymptomatic and presymptomatic SARSCoV-2 infections: A living systematic review and meta-analysis. PLoS Medicine. 2020;17(9):e1003346.

62. Byambasuren O, Cardona M, Clark J, Glasziou P, Bell K, McLaws M-L. Estimating the extent of asymptomatic COVID-19 and its potential for community transmission: Systematic review and meta-analysis. JAMMI. 2020;5(4):223-34.

63. Jefferson T, Onakpoya IJ, Spencer EA, Pluddemann A, Heneghan CJ, Brassey J, et al. Transmission of Severe Acute Respiratory Syndrome Coronavirus-2 (SARS-CoV-2) from pre and asymptomatic infected individuals: a systematic review. Clinical Microbiology and Infection. 2021.

64. Koh WC, Naing L, Chaw L, Rosledzana MA, Alikhan MF, Jamaludin SA, et al. What do we know about SARS-CoV-2 transmission? A systematic review and meta-analysis of the secondary attack rate and associated risk factors. PLoS ONE. 2020;15(10):e0240205.

65. Madewell ZJ, Yang Y, Longini IM, Jr., Halloran ME, Dean NE. Household transmission of SARS-CoV-2: a systematic review and meta-analysis of secondary attack rate. medRxiv : the preprint server for health sciences. 2020.

66. Madewell ZJ, Yang Y, Longini IM, Dean NE, Halloran ME. Household Transmission of SARS-CoV-2: A Systematic Review and Meta-analysis. JAMA netw. 2020:e2031756.

67. Qiu X, Nergiz AI, Maraolo AE, Bogoch II, Low N, Cevik M. The role of asymptomatic and pre-symptomatic infection in SARS-CoV-2 transmission-a living systematic review. Clinical Microbiology and Infection. 2021;27(4):511-9.

68. Savvides C, Siegel R. Asymptomatic and presymptomatic transmission of SARS-CoV-2: A systematic review. medRxiv : the preprint server for health sciences. 2020.

69. Yanes-Lane M, Fregonese F, Bastos M, Perlman-Arrow S, Winters N, Campbell JR, et al. Proportion of asymptomatic infection among COVID-19 positive persons and their transmission potential: A systematic review and meta-analysis. PLoS ONE. 2020;15(11 November):e0241536.

70. Hellewell J, Abbott S, Gimma A, Bosse NI, Jarvis CI, Russell TW, et al. Feasibility of controlling COVID-19 outbreaks by isolation of cases and contacts. Lancet Glob Health. 2020;8(4):e488-e96.

71. Koo JR, Cook AR, Park M, Sun Y, Sun H, Lim JT, et al. Interventions to mitigate early spread of SARS-CoV-2 in Singapore: a modelling study. The Lancet Infectious Diseases. 2020;23:23.

72. Wang L, Duan Y, Zhang W, Liang J, Xu J, Zhang Y, et al. Epidemiologic and Clinical Characteristics of 26 Cases of COVID-19 Arising from Patient-to-Patient Transmission in Liaocheng, China. Clinical Epidemiology. 2020;12:387-91.

73. Kakimoto K, Kamiya H, Yamagishi T, Matsui T, Suzuki M, Wakita T. Initial Investigation of Transmission of COVID-19 Among Crew Members During Quarantine of a Cruise Ship - Yokohama, Japan, February 2020. Mmwr. 2020;Morbidity and mortality weekly report. 69(11):312-3.

74. Kimball A, Hatfield KM, Arons M, James A, Taylor J, Spicer K, et al. Asymptomatic and Presymptomatic SARS-CoV-2 Infections in Residents of a Long-Term Care Skilled Nursing Facility - King County, Washington, March 2020. MMWR Morb Mortal Wkly Rep. 2020;69(13):377-81.

75. Bai SL, Wang JY, Zhou YQ, Yu DS, Gao XM, Li LL, et al. Analysis of the first cluster of cases in a family of novel coronavirus pneumonia in Gansu Province. [Chinese]. Zhonghua yu fang yi xue za zhi [Chinese journal of preventive medicine]. 2020;54:E005.

76. Ferguson NM, Laydon D, Nedjati-Gilani G, Imai N, Ainslie K, Baguelin M, et al. Report 9: Impact of non-pharmaceutical interventions (NPIs) to reduce COVID-19 mortality and healthcare demand. Imperial College London (16-03-2020). 2020.

77. Arons MM, Hatfield KM, Reddy SC, Kimball A, James A, Jacobs JR, et al. Presymptomatic SARS-CoV-2 Infections and Transmission in a Skilled Nursing Facility. New England Journal of Medicine. 2020;382(22):2081-90.

78. Liu T, Hu J, Xiao J, He G, Kang M, Rong Z, et al. Time-varying transmission dynamics of Novel Coronavirus Pneumonia in China. bioRxiv. 2020.

79. Tobolowsky FA, Gonzales E, Self JL, Rao CY, Keating R, Marx GE, et al. COVID-19 Outbreak Among Three Affiliated Homeless Service Sites - King County, Washington, 2020. MMWR Morb Mortal Wkly Rep. 2020;69(17):523-6.

80. Roxby AC, Greninger AL, Hatfield KM, Lynch JB, Dellit TH, James A, et al. Detection of SARS-CoV-2 Among Residents and Staff Members of an Independent and Assisted Living Community for Older Adults - Seattle, Washington, 2020. MMWR Morb Mortal Wkly Rep. 2020;69(14):416-8.

81. Roxby AC, Greninger AL, Hatfield KM, Lynch JB, Dellit TH, James A, et al. Outbreak Investigation of COVID-19 Among Residents and Staff of an Independent and Assisted Living Community for Older Adults in Seattle, Washington. JAMA Intern Med. 2020;21:21.

82. Mosites E, Parker EM, Clarke KEN, Gaeta JM, Baggett TP, Imbert E, et al. Assessment of SARS-CoV-2 Infection Prevalence in Homeless Shelters - Four U.S. Cities, March 27-April 15, 2020. MMWR Morb Mortal Wkly Rep. 2020;69(17):521-2.
